# Nutrition-Sensitive Agriculture: A Systematic Review of Impact Pathways to Nutrition Outcomes

**DOI:** 10.1093/advances/nmaa103

**Published:** 2020-09-24

**Authors:** Indu K Sharma, Sabina Di Prima, Dirk Essink, Jacqueline E W Broerse

**Affiliations:** Athena Institute, Faculty of Science, Vrije Universiteit Amsterdam, Amsterdam, Netherlands; Athena Institute, Faculty of Science, Vrije Universiteit Amsterdam, Amsterdam, Netherlands; Athena Institute, Faculty of Science, Vrije Universiteit Amsterdam, Amsterdam, Netherlands; Athena Institute, Faculty of Science, Vrije Universiteit Amsterdam, Amsterdam, Netherlands

**Keywords:** nutrition education, food, systematic review, impact, pathway, low- and middle-income countries, diet, undernutrition, multisector nutrition, nutrition-sensitive interventions

## Abstract

The role of agriculture in reducing undernutrition is widely recognized, yet there is also consensus on the need to make the sector nutrition-sensitive. Evidence on the impact pathways from nutrition-sensitive agriculture (NSA) interventions, agricultural interventions with specific nutrition objectives, and actions detailing each temporal stage to reach nutrition outcomes is limited, however. We thus synthesized study results regarding impact of NSA interventions on nutrition outcomes relating to undernutrition, and constructed an impact pathway by mapping the evidence on each temporal stage from interventions to nutrition outcomes. We used Preferred Reporting Items for Systematic Reviews and Meta-Analyses to conduct and report our systematic review of studies on NSA interventions implemented in low- and lower-middle-income countries. Forty-three studies that met the inclusion criteria were extracted and synthesized across impact and pathways analyses. We carried out a thematic analysis of the effect of NSA interventions using evidence-based indicators and constructed the pathways by adopting a published framework on agriculture to nutrition pathways. Our findings reveal that NSA interventions can significantly improve dietary practices, and have the potential to enhance care practices and reduce occurrence of diseases, indicating their effectiveness in simultaneously addressing multiple determinants of undernutrition. However, NSA interventions have a lesser impact on nutritional status. NSA interventions lead to nutrition outcomes through 5 key pathways: food production, nutrition-related knowledge, agricultural income, women's empowerment, and strengthening of local institutions. We emphasize the need to carefully design, implement, and evaluate interventions with consideration for factors affecting impact pathways. Future research should focus on the effect of interventions combining multisector components, and pathways through non-food-production-related income, women's empowerment, strengthening of local institutions, food prices at intervention level, and expenditure on health care.

## Teaser

This review synthesizes the impact of NSA interventions on nutrition outcomes and constructs pathways by mapping the evidence on each temporal stage, from the interventions to the nutrition outcomes.

## Introduction

Undernutrition is a major global health problem, contributing to 45% of deaths among children under the age of 5 y (1) and 21% of disability-adjusted life-years (2). The effects of undernutrition are multifaceted and go beyond health, and include reduced educational attainment and loss of productivity (3). Children and women are most often adversely affected by undernutrition, with 1 in 5 children under the age of 5 y having a low height for their age, and 7.5% of children exhibiting wasting (4). Likewise, anemia among women remains globally high, with 32.8% anemic in 2016, which has slightly increased from 31.6% in 2000 (4). In addition, progress in addressing undernutrition is extremely slow, and countries are off-track in achieving the 2030 Sustainable Development Goals targets for reducing anemia, childhood stunting, and wasting (4). More effective approaches are therefore needed to address the problem of undernutrition.

The agriculture sector can potentially play a crucial role in responding to the problem of undernutrition by directly addressing inadequate access to nutrient-rich food, which is a key underlying determinant of malnutrition (5–7). Food- and agriculture-related factors contribute to one-third of stunting (8), and the majority of the population in low- and lower-middle-income countries (LMICs) depends on agriculture for their livelihoods (6, 9), which increases the role of the agriculture sector to address the problem. The EAT–*Lancet* Commission also highlighted agriculture and the food system as a current major contributor to poor health, and urged the transformation of food production and diets (10). Past studies highlight the contribution of agriculture to nutrition outcomes through 4 key pathways: *1*) agricultural production improving availability of and access to nutrient-rich foods (6, 8, 11–14), *2*) agricultural income increasing purchasing power (6, 8, 11–14), *3*) agricultural policies affecting food price (6, 11, 13), and *4*) agricultural interventions empowering women to improve their nutrition outcomes and those of their children (12, 14).

There is a broad realization that agricultural interventions will have to become nutrition-sensitive to improve food access and attain global nutrition targets. One approach has been the concept of nutrition-sensitive agriculture (NSA). NSA interventions go beyond the conventional idea of increasing food production by incorporating specific nutrition objectives and actions in the design and implementation of agriculture interventions (15, 16). A review highlighted the potential of NSA interventions to address all underlying causes of undernutrition (17) identified in the framework of UNICEF: household food insecurity, inadequate care practices, lack of access to health services, and unhealthy household environments (7). Thus, as a part of the broader multisectoral response to nutrition, NSA can shape not only dietary practices but also underlying health- and environment-related causes that go beyond access to food (17).

Although several studies published in the last 15 y have focused on agriculture interventions (6, 13, 14, 16, 18–25) and their impact on nutrition-related outcomes (6, 17–27), reviews have rarely explicated the impact pathways from agricultural interventions with specific nutrition objectives and actions detailing each temporal stage to reach nutrition outcomes. Five of these studies reviewed the impact and pathways of agriculture interventions to nutrition outcomes, irrespective of whether they included specific nutrition actions and objectives, and also have not detailed each stage from interventions to impact (13, 14, 19, 23, 27). Some of these studies focused either on specific regions in South Asia (14, 19) or India (13), on a single pathway through women's empowerment (23), or on a single intervention on input subsidy (27). Two reviews that looked exclusively at the interventions regarding specific nutrition objectives did not investigate the temporal stage of pathways from interventions to nutrition outcomes (16, 22), and 1 of the 2 did not have a focus on LMICs (22). Thus, there is limited evidence on the pathways depicting the effect on each temporal stage from NSA interventions to nutrition outcomes: for example, from interventions to food production to income to food expenditure to dietary practices to nutritional status, or another pathway from interventions to women's empowerment to food expenditure to dietary practices to nutritional status. A few studies have also stressed the need for assessments of interventions that focus not only on their impact on nutritional status, but also on a full spectrum of underlying determinants, such as food access, dietary practices, food security, women's empowerment, health environment, and health status (17), and the pathways through which these interventions affect agriculture, nutrition-related practices, and nutrition outcomes (17, 25). To address these gaps, we systematically reviewed the evidence on agriculture interventions with specific nutrition objectives and actions to *1*) synthesize the impact on nutrition outcomes relating to undernutrition, and *2*) construct pathways by mapping the evidence on each temporal stage, from the interventions to the nutrition outcomes. Such evidence will help maximize the role agriculture can play in achieving long-term nutrition outcomes. Enhanced understanding of the impact pathways and their temporal progression can facilitate early identification of potential bottlenecks that may inhibit agriculture's full potential, and stimulate adaptive actions during implementation of interventions. Furthermore, our evidence can prompt agricultural programs to pursue a broader spectrum of specific nutrition objectives beyond food production, thus simultaneously addressing multiple underlying causes of undernutrition.

## Methods

### Protocol and registration

We conducted this review using the guideline from Preferred Reporting Items for Systematic Reviews and Meta-Analyses (28), and registered the protocol in the International Prospective Register of Ongoing Systematic Reviews as CRD42018108308 (29).

### Search strategy

We systematically searched published studies in the electronic bibliographic databases PubMed, Embase, Web of Science, and Scopus in 3 steps. First, we identified 5 search concepts: agriculture, nutrition outcome, multiple sectors, nutrition-sensitive interventions, and LMICs (29). We then explored subconcepts, e.g., homestead food production (HFP) (6, 14, 16, 18, 22), nutrition indicators, health sector, and countries within LMICs (30). This resulted in the following general search syntax: [(agriculture general topic, value chains/value crops, (bio-) fortification, homestead production, livestock and dairy, water management, aquaculture, and agricultural extension) AND (food OR diet OR nutritional status) OR nutrition-sensitive interventions AND (multisector general topic, health, education, water, sanitation and hygiene, social protection and natural resource management) AND LMICs]. Third, we scanned the reference lists of key articles, such as Ruel et al. (16), verified their inclusion in the search results, and expanded the syntax. Although this syntax may look complex, it allowed us to uncover a number of relevant and insightful articles not included in previous reviews, thus contributing to the body of evidence on this topic. The search strategy was piloted in PubMed in March 2019 and replicated in the other databases, as **Supplemental Table 1** presents.

### Eligibility criteria

As **Table 1** shows, we used 8 criteria to determine eligibility for inclusion: peer-reviewed, empirical studies published after 2000 in the English language, based on lower- and middle-income countries, with specific participants, interventions, comparison, and outcomes (28). The participants were women, children, or household members in general. Adapting from past reviews (6, 16), NSA interventions were defined as agriculture interventions with an objective to improve nutrition-outcomes and incorporate specific nutrition actions to achieve the objective. We further limited the review to studies that used either a comparison between intervention and control, differences within a single population before and after interventions, or a cross-sectional comparison between beneficiaries and nonbeneficiaries. The final inclusion criterion for studies was reporting on ≥1 of the 3 levels of outcomes concerning undernutrition (17), namely nutritional status, using biochemical or anthropometric measurements; diet, health status/disease, or food consumption; or food access, care practices, and health environment.

**TABLE 1:** Inclusion and exclusion criteria for the selection of studies, NSA impact pathways to nutrition outcomes^1^

Criterion	Included	Excluded	Screening phase applied	Rationale
Topic related to NSA	Topic relevant to NSA, such as nutrition, agriculture, or nutrition-sensitive interventions.	Irrelevant topics, such as disease conditions, plant nutrients, or arsenic contamination.	Title screening	Relevance
Study characteristics				
Participants	Children and women of reproductive age or household-level impact in general, as reported by individual studies.	Populations with specific health conditions such as diseases, elderly populations, and hospitalized patients.	Abstract and full text	Children and women in LMICs are most often affected by undernutrition (2).
Interventions	Agriculture interventions that incorporate specific nutrition objectives and actions and address ≥1 of the following domains: health, environment (including WASH or natural resource management), education, and social and financial protection.	Agriculture interventions alone, without nutrition-specific objectives and actions, and that do not go beyond the agriculture sector.	Abstract and full text	Definition of NSA interventions: incorporate specific nutrition objectives and actions (6, 15), and address health, environment, education, social, and financial dimensions (15).
Comparators	Studies comparing outcomes between different groups; or preintervention; or pre- and postintervention within single group.	One-time cross-sectional studies not comparing the effects between different groups; or same group before and after intervention.	Abstract and full text	
Outcome	Impact on undernutrition: nutritional status using biochemical or anthropometric measurements; diet, health status, or food consumption; or food access, care practices, or health environment, based on evidence-based indicators (17).	Overnutrition status, such as obesity.	Abstract and full text	Presents evidence-based data on impact of agriculture on nutrition outcomes; evidence-based indicators (17).
Report characteristics				
Publication type	Peer-reviewed empirical studies	Meta-analyses and/or reviews, gray literature, study protocols, opinion papers, discussion papers, commentaries, letters and editorials, conference abstracts.	Title and abstract	Peer-reviewed articles are stronger in quality of content and methodology and therefore studies without peer review will be excluded. Further, meta-analyses or reviews do not provide detailed insights on the empirical articles and therefore were excluded.
Publication years	2000 and after		Abstract and full text	The Millennium Development Goals commitment was endorsed in 2000. The declaration is a key milestone in nutrition.
Language	English		Title screening	
Location	LMICs, based on the World Bank's classification (30).	Upper-middle-income and high-income countries	Title, abstract, and full text	LMICs are mostly affected by undernutrition.
Inclusion for impact and pathways analysis^2^				
Information on impact	The impact analysis included outcomes measured using correlation, chi-square, ORs (association), difference-in-difference estimates, and treatment or intervention effects. We used *P* values when the effect size was not available.	Studies presenting proportion only, without studying significant effect and/or difference between groups.	Full text	Descriptive analysis presenting proportions only allows making summations about the change and may reflect bias.
Information on pathways	Studies reporting entry points of pathways from NSA to nutrition, such as food production, agricultural income, food price, women's empowerment (6, 11–14), and knowledge on nutrition, WASH, and/or health.	Studies reporting on impact only without pathways.	Full text	Impacts alone do not give insights into the pathways.

1 LMICs, low- and lower-middle-income countries; NSA, nutrition-sensitive agriculture; WASH, water, sanitation, and hygiene.

2 This criterion emerged during analysis, because not all studies reporting on impact detailed the elements of the pathways, for which we cannot say with a high level of certainty how and through which entry points (e.g., food production or knowledge or income or women's empowerment) these interventions contributed to the nutrition outcomes.

### Study selection

We exported search results to EndNote X8 software (Clarivate Analytics), where we removed duplicates, screened the titles and abstracts for eligibility, and read the remaining full texts for inclusion. The selection process trialed by the first and second authors during a preliminary search yielded 25 articles and revealed a high interrater agreement. The preliminary search syntax included the effect on nutritional status and 1 other outcome, such as dietary practices. We subsequently refined the syntax, based on an insight from a past review that NSA interventions should measure impact on intermediate outcomes, such as dietary practices, in addition to the nutritional status (17). The selection that used the refined syntax was carried out by the first author and generated 43 articles for inclusion, including the original 25. We further grouped the studies into impact and pathways analyses, because not all studies reporting on impact detailed the elements of the pathways. That means we cannot say with a high level of certainty how and through which entry points (e.g., food production, agricultural income) these interventions contributed to the nutrition outcomes (see Table 1). The impact analysis group included studies with a quantitative design that measured effects on nutrition outcomes, regardless of the information about the entry points to the pathways. The pathways analysis, on the other hand, only included studies reporting 2 types of findings: *1*) nutrition outcomes using either a quantitative or qualitative design, and *2*) ≥1 entry point to pathways that led to nutrition outcomes, such as food production, agricultural income, food price, and women's empowerment (6, 11, 13, 14, 16), or as emerged during data synthesis.

### Data collection process

Data collection included the following information: publication details, study setting, study design, interventions, data collection method, study population, data analysis, nutrition outcome, and pathways. The first 2 authors independently extracted data from 30% of the studies, with continuation by the first author in the remaining studies and review by the second author.

### Risk of bias assessment

We selected 2 tools to assess the risk of bias in the reviewed studies. We used the tool developed by the Effective Public Health Practice Project for quantitative studies, because it uses a generic scale that is comparable across a range of study designs (31, 32). We labeled the high-quality studies as having a low risk of bias, medium quality as medium risk of bias, and low quality as high risk of bias. For qualitative studies, we applied the Critical Appraisal Skills Programme scale to rate the risk of bias as 9–10 (low), 6–8 (medium), and <6 (high) (33).

### Synthesis of results

We synthesized the impact and pathways analyses using 2 strategies. The impact analysis included the outcomes measured using correlation, chi-square, ORs (association), difference-in-difference estimates, and treatment or intervention effects. We used *P* values when the effect size was not available. The pathways analysis involved a 3-step process: *1*) construction of pathways as reported in each study, *2*) grouping individual pathways across similar intervention categories, and *3*) merging interventions-based pathways into a consolidated framework. We constructed the pathways by adapting the framework of Kadiyala et al. (13) and mapped each element of the pathways across temporal stages. This framework recognizes 6 pathways: food production; agricultural income; food prices; women in agriculture, intrahousehold decision-making, and resource allocation; maternal employment in agriculture, childcare, and feeding; and women in agriculture and maternal nutrition and health status (13). We repackaged the 3 gender-related pathways into 1 “women's empowerment” pathway because the studies reviewed focused on empowerment of women and lacked explicit information on agriculture–gender linkages. We mapped the pathways constructed across each temporal stage of outputs; short-, medium-, and long-term outcomes; and the impact using the logic model of the Strong Through Every Mile program (34). We hypothesized that NSA interventions deliver outputs on food production (13, 35) and knowledge on nutrition; water, sanitation, and hygiene (WASH); or health (35), resulting in short-term outcomes on agricultural income/selling, food price, food preservation, processing and storage, nutrition-related attitude, and women's empowerment. The short-term outcomes precede the medium-term outcomes concerning the underlying determinants of malnutrition, namely care practices, household food security, household living environment, and services (7). The medium-term outcomes contribute to long-term outcomes representing immediate causes of undernutrition and include dietary practices and diseases, eventually resulting in the impact on nutritional status (7). For the elements not listed in the above categories, we classified them as they emerged from the studies we reviewed.

## Results

### Study selection

**FIGURE 1:**
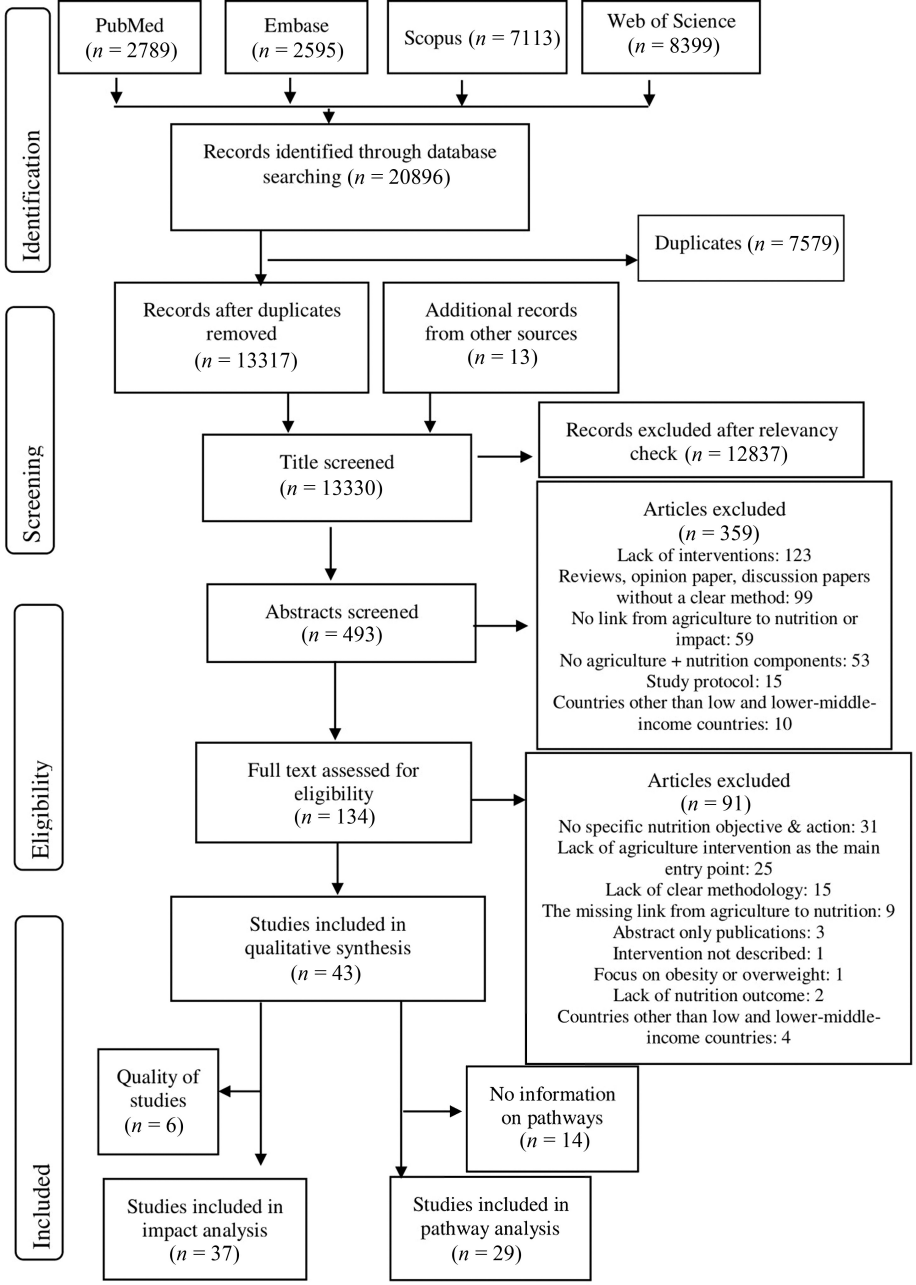
Flow diagram of the study selection process, nutrition-sensitive agriculture impact pathways to nutrition outcomes.

The search of bibliographic databases retrieved 20,896 studies, resulting in the final inclusion of 43 articles that reported on the impact of NSA interventions (*n* = 37) and their pathways to nutrition outcomes ( *n* = 29) (see **Figure 1**).

### Study characteristics

**FIGURE 2:**
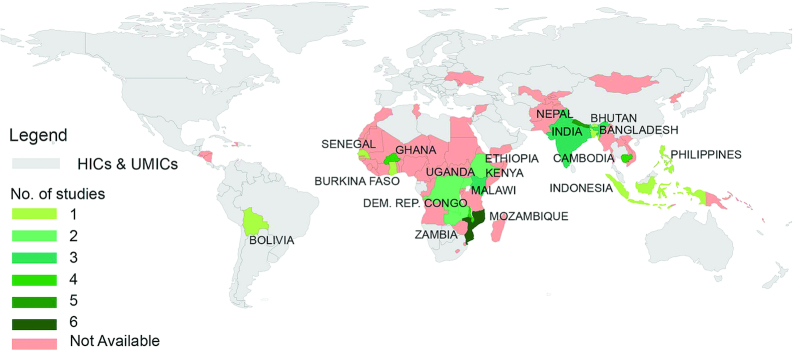
Distribution of studies reviewed across LMICs, nutrition-sensitive agriculture impact pathways to nutrition outcomes (*n* = 43). HICs, high-income countries; LMICs, low- and lower-middle-income countries; UMICs, upper-middle-income countries.

Most studies used a quantitative design (*n* = 39) and were published in 2017 (*n* = 9). They represented 18 countries (see   **Figure 2**), but were mostly from Mozambique (*n* = 6). The quantitative studies included randomized controlled trials/experiments (*n* = 22), quasi-experiments (*n* = 6), repeated cross-sectional designs (*n* = 4), longitudinal/cohort studies (*n* = 3), a 1-time cross-sectional study comparing beneficiaries and controls (*n* = 2), nonrandomized interventions (*n* = 1), and a pair-matched design (*n* = 1). The studies reported 13 types of interventions and 3 categories of outcomes among children, women, men, adults, elderly, and sick persons. Thirty-six studies compared outcomes between intervention and control groups, or between beneficiaries and nonbeneficiaries, whereas 7 compared the effects within the same group before and after an intervention. Most studies using a quantitative design had a medium risk of bias (*n* = 21), followed by high (*n* = 15) and low ( *n* = 3) risk. All studies applying a qualitative design had a medium risk of bias (*n* = 3), and those employing mixed-methods designs had a high risk of bias. **Table 2** shows the study characteristics and findings, with details presented in **Supplemental Table 2**.

**TABLE 2:** Characteristics and findings of individual studies, NSA impact pathways to nutrition outcomes^1^

Source; country	Study design	Interventions	Results on pathways and impact	Impact analysis (*n* = 37)	Pathway analysis (*n* = 29)	Risk of bias
Berti and Cossio (59); Bolivia	Nonrandomized intervention	Poultry: inputs and trainingNutrition education	↑ Egg production (established in 80% of intervention HHs)↑ Egg consumption, no change in the consumption of dairy, fruits, legumes, meat; iron: ↑ intake (mg/d), no change in mg/1000 kcal; no impact on intakes of energy, protein, calcium, zinc, thiamin, riboflavin, or niacin	X	X	High
Birdi and Shah (60); India	Longitudinal study (2-y follow-up)	Kitchen garden input IEC on nutrition	↑ Kitchen garden practice (fruits and vegetables) in all seasons↑ GLV variety, no change in quantity↑ Consumption of fruits and eggs, no change in variety of cereals and pulses No impact on using intervention plants in weaning		X	High
Bushamuka et al. (40); Bangladesh	One time cross-sectional	Homestead gardening, nutrition education sessions, and gender interventions	↑ Production and diversification of vegetables and fruits;↑ Income and ↑spending on food, health, education, clothing, productive assets, housing, and social activities**↑**WE: participation in group meetings, determining daily workload, visiting stores/markets, economic contribution to HH and decision making;↑ Consumption of vegetables and fruits at household	X	X	High
Diana et al. (47); Indonesia	Quasi- experimental study	*1*) Intervention: HG inputs and nutrition extension*2*) Control: no interventions	No impact on size of home garden↑ Nutrition knowledge in intervention group↓ Food expenditure due to increased production No impact on vegetable consumption, or vitamin A intake at household	X	X	High
Doocy et al. (61); DR Congo	Quasi-experimental study	*1*) FFS: intensive agriculture production; *2*) F2F: less resource activities; *3*) PM2A: nutrition BCC, HG, and health; *4*) WEG: literacy, business, and income generation	Pathways not reported↑Children DDS: PM2A and FFS, no impact on others↑ Mean Meal Frequency in children: PM2A, FFS and WEG ↑ Children achieving MMF or MAD in PM2A onlyNo impact on stunting or underweight in children	X		Medium
Doocy et al. (62); DR Congo	Quasi-experimental study	*1*) FFS: intensive agriculture production; *2*) F2F: less resource activities; *3*) PM2A: nutrition BCC, HG, and health; *4*) WEG: literacy, business, and income generation	Pathways not reported↑ Mean HDDS: in FFS PM2A and WEG, not on F2F↑ food security: in PM2A , WEG, FFS, and F2F	X		Medium
Boedecker et al. (63); Kenya	Pair-matched study	Workshops and training on KG, poultry, and nutrition; door-to-door nutrition education	Pathways not reported↑ Consumption of legumes and nuts and flesh foods in children; no effect: eggs, fruits and vegetables↑ Child DDS and share of children reaching MDD, no impact on MAR No impact on food group consumption in women other than dairy, No impact on DDS, MDD, MAR in women	X		High
Dulal et al. (64); Nepal	Repeated cross-sectional (ben. and nonben.)	Training and inputs on HG and poultry; essential nutrition and hygiene actions	Pathways not reported↑ CDD in Terai region in winter no impact in mountain region or rainy season↑ Maternal dietary diversity in winter; no impact in rainy season	X		High
Heckert et al. (54); Burkina Faso	RCT	*1*) Control*2*) EHFP, WE, BCC led by OWL*3*) EHFP, WE, BCC led by HC members	↑ WE score reduced wasting owing to increased spousal communication, purchasing decision, health care decision, and family planning decision No significant associations between the WE and change in Hb in children↑ Purchasing decision and spousal communication; no effect on health care or FP decisions Impact: no effect on Hb, marginal effect on wasting	X	X	Medium
Jones et al. (48); Nepal	One-time cross-sectional survey (interventions and control)	KG (inputs, training, and extension) Nutrition, food preservation, and WASH education (training and household visit)	↑ Production for consumption↑ Buying vegetables for consumption by controls↑ Nutrition knowledge (vitamin A, iron-rich foods, anemia, IYCF)↑ Food preservation;↑Consumption variety and frequency of vegetables and mango; no difference on consumption of ASFsNo impact on BF: colostrum, length of EBF↑ Complementary feeding;↑ Handwashing practice of caregivers and childrenNo impact on access to sanitation facility	X	X	High
Marquis et al. (65); Ghana	Cluster RCT	*1*) Intervention: inputs and training on HG and poultry; nutrition and health education*2*) Control	Pathways not reported↑ MDD; no change in consumption of egg↑ LAZ/HAZ score ↑ WAZ score; no difference in WLZ/WHZ score	X		Low
Murty et al. (45); India	Repeated cross-sectional design	Training and inputs on HG, crop diversification, and backyard poultryHealth and nutrition messaging	↑Vegetables and fruits garden and poultry rearing ↑ Knowledge on eating more food by pregnant women and cause of diseases ↑ Income from vegetables, selling of fertilized eggs ↑ Frequency of GLVs cooked and HH cooking GLVs >3 times↑ Egg consumption frequency and quantity↑ Practices on IYCF (EBF, complementary feeding, and ↓ prelacteal feeding)↑ Mother's handwashing with soap before child feeding No impact on access to bathroom and latrine**↓** Underweight, no impact on birth weight	X	X	High
Olney et al. (41); Cambodia	Repeated cross-sectional design	Training and inputs on homestead production (vegetable, poultry/duck)Nutrition education	↑ Vegetable production, no impact on animal production or ownership Household: ↑ DDS, ↑ consumption of dark GLVs, no impact on consumption of non–dark leafy vegetables or ASFs Children: No impact on DDS, ↑ consumption of egg; Children: No impact on Hb, anemia, or diarrhea, ↓ prevalence of fever No impact on stunting, wasting, or underweight, or HAZ, WHZ, and WAZ; Women: no impact on DDS or consumption of micronutrient-rich foods, No impact on Hb, diarrhea, weight, or BMI	X	X	High
Olney et al. (35); Cambodia	Qualitative design	Training and inputs on homestead production (vegetable and poultry/duck)Health and nutrition education	↑ Production of vegetables, fruits, and poultry↑ Knowledge on BF, food to add in porridge, safe food preparation but low knowledge on complementary feeding, need of more fluids for sick children,↑ Knowledge on micronutrients, limited knowledge on anemia;↑ Income from vegetables, less income from fruits and poultry↑ Consumption of vegetables and poultry meat; less consumption of fruit and eggs from market Children: ↑ consumption of rich-porridge, marginal ↑ handwashing practices		X	Medium
Olney et al. (42); Burkina Faso	Cluster RCT	*1*) Control: without interventions*2*) EHFP including poultry; BCC-WE led by OWL *3*) EHFP including poultry; BCC-WE led by HC members	↑ Production of vitamin A–rich fruits and vegetables and other fruits↑ Knowledge on need of liquid and semisolid food at 6 mo↑ Knowledge on washing hands before feeding child, no change in other attributes of handwashing knowledge; Marginal ↑ HDDS in group 3Marginal ↑ MDD (6–12.9 mo) in group 3, no impact on iron-rich food intake↑ Hb for 3- to 5.9-mo-old child and marginal ↑ Hb for 3- to 12.9-mo-old child in group 3;↓ Anemia in group 3 (3- to 5.9-mo-old child) no impact in others; ↓ Diarrhea for 3- to 12.9-mo-old child in group 3; marginal ↓ in wasting in group 3 (3–12.9 mo old); no impact on stunting or underweight	X	X	Medium
Olney et al. (55); Burkina Faso	Cluster RCT	*1*) Control: without interventions* 2*) EHFP including poultry, BCC led by OWL and WE *3*) EHFP including poultry, BCC led by HC members and WE Analysis: intervention 1 and 2 vs. control	WE: ↑ Total WE score, meeting with other women, and purchasing decision; marginal ↑ health care decision; no impact on spousal communication, social support, family planning decision, or decision on IYCF Household: food expenditure, no impact on HDDS; ↑ consumption of fish and seafoods, fruits; marginal ↑ consumption of meat and poultry; no impact on consumption of vegetables, milk and dairy, legumes, nuts and pulses, oils and fats;	X	X	Medium
			Mothers: Marginal ↑ DDS; ↑ consumption of fruit, cereals, grain marginal↑ Intake of meat/poultry; no impact on consumption of vegetables, milk/dairy, eggs, fish and seafoods, oil, fats, legumes, nuts and seeds, roots and tubers; Mothers: ↓ underweight, ↑ BMI for women underweight at baseline			
Osei et al. (66); Nepal	Prospective cluster RCT	*1*) EHFP including poultry and MNP supplementation*2*) EHFP including poultry*3*) Control: no interventions	Pathways not reportedNo impact on fever, no significant ↓ on Hb or anemia in children;↓ Diarrhea in children for EHFP; no impact for EHFP + MNP No impact on fever, stunting, wasting, or underweight in children	X		Low
Osei et al. (43); Nepal	RCT	Intervention: training and inputs on HG and poultry; nutrition education by FCHVs; public health services participationControl: no interventions	↑ EHFP on vegetables and/or poultry; ↓Number food-insecure HHs; Children: ↑ colostrum feeding and EBF, ↑ MDD, MMF, and MAD, ↓ anemia among children 0–23 mo old No impact on stunting, wasting, or underweight Women:↓ anemia and underweight	X	X	Medium
Michaux et al. (67); Cambodia	RCT	*1*) EHFP: HG training and inputs; BCC on nutrition, hygiene; WE, marketing*2*) EHFP and fish training and inputs, pond establish/rehabilitate*3*) Control	Pathways not reportedChildren: marginal ↑ Hb in groups 2 and 1; ↓ anemia in group 1; no impact on stunting, wasting, or underweight;Women: no impact on Hb or anemia; ↑ RBP concentration in group 2; no impact on ferritin, serum zinc, AGP, or CRP No impact on women's underweight based on BMI	X		Medium
Kuchenbecker et al. (56); Malawi	RCT	Control: agriculture onlyIntervention: agriculture (inputs and training for crops and livestock); nutrition (IYCF and education sessions)	No change in production; Children: ↑ consumption of eggs and groundnuts; no impact on consumption of flesh food, fruits and vegetables, dairy products, legumes, or other nuts Children: ↑ MDD and MAD; no impact on MMF; ↑ access to improved sanitation; no change in access to drinking water source Children: no impact on HAZ, WHZ, or WAZ Mothers: no impact on BMI	X	X	Medium
Kumar et al. (52); Zambia	Cluster RCT	*1*) Intervention: agriculture–gender*2*) Intervention: agriculture–gender–BCCAgriculture: inputs and training on HG, and livestockBCC: IYCF, hygiene, health-seeking behaviorGender: gender equity and WE*3*) Control: standard government services	WE (for combined interventions vs. control): ↑ social capital, asset access, financial empowerment, and agriculture empowerment; no impact on decision making, spousal relationship, gender equality, asset selling; decrease buying power and time for domestic work and childcare; ↑ agricultural time Knowledge on BF varying, no difference in practice (score);↑ MMF: group 2 vs. 1; no impact on MDD, MAD, iron-rich food consumption↓cough/cold, diarrhea for group 2 vs. 1, no differential impact on fever;↑ WHZ score: group 1 vs. 3; no impact on HAZ score, wasting, or stunting	X	X	High
Rosenberg et al. (39); Zambia	RCT	Pool all treatments survey* 1*) Intervention: agriculture–gender* 2*) Intervention: agriculture–gender–BCC Agriculture: inputs and training on HG, and livestock BCC: IYCF, hygiene, health-seeking behavior *3*) Control: standard government services	↑ Agricultural production diversity and production of groundnuts, rape, tomatoes; no increase in meat and fish production;↑ Cottonseed production (not targeted by project)Income/selling from nonfood cotton and food-based agriculture from selling targeted crops; HH: ↑ Total food groups consumption, including pulses, legumes and nuts, meat, and fish; no effect on egg or vegetables/fruits No impact on hunger scale and food insecurity; Children: ↑ consumption of pulses, legumes, and nuts; no effect on egg, vegetables or fruits, or total food groups consumption, or dietary diversity Women: no impact on total food groups consumption, or dietary diversity	X	X	High
Reinbott et al. (57); Cambodia	RCT	*1*) Intervention: agriculture and nutritionAgriculture: inputs and trainingNutrition: IYCF promotion and nutrition education sessions*2*) Control: agriculture only	No impact: Ownership of animals, no impact on access to fruit or HG; No impact on HDDS Children: ↑ DDS and MAD, no impact on MMF; Children: ↑ consumption of vitamin A–rich foods and other fruits and vegetables; no impact on the consumption of ASFs; no difference on introduction of semisolid foods at 6 mo, or access to sanitation facility or drinking water source at endline No difference on fever, diarrhea, or ARI at endline, in children No difference on HAZ, WHZ, or WAZ scores at endline, in children	X	X	Medium
de Brauw et al. (68); Mozambique	RCT	Interventions: agricultural component—OFSP vine distribution, extension, and agronomics training, nutrition education Groups: *1*) intensive; and *2*) less intensive treatments; and 3)Control	Pathways not reported;↑ vitamin A density in diet and dietary diversity, higher diversity for more intense participation in nutrition and agriculture,↑ MMDA, ↑ MMDA for HHs and women with nutrition promoters, ↑ Dietary diversity score-higher for ones with nutrition promoter; No impact on wasting/WHZ score	X		Medium
de Brauw et al. (37); Mozambique and Uganda	Randomized field experiments trial	*1*) OFSP vine distribution and nutrition training for 2 y*2*) OFSP vine distribution for 2 y, nutrition training in year 1 only*3*) Control	↑ OFSP adoption, total area for OFSP↑ Knowledge on vitamin A and OFSP as a source of vitamin A Knowledge has no significant effect on adoption; Children: ↑ dietary intake of vitamin A	X	X	Medium
Girard et al. (44); Kenya	Longitudinal cohort study	*1*) Intervention: clinic-based nutrition counselling linked to OFSP and crops *2*) Control group: clinic-based nutrition counselling	↑ Knowledge on nutrition, health, and vitamin A; no impact on IYCF knowledge; Women: ↑ consumption of vitamin A–rich fruits and vegetables, ↑ intake of β-carotene and vitamin A; no impact on energy intake or WDDS Women: ↑ vitamin A adequacy; ↓ odds of low RBP; no impact on mean RBP;↓ Odds of anemia in late pregnancy only, no impact for the overall period; no impact on Hb or mean MUAC	X	X	Medium
Hotz et al. (69); Mozambique	Randomized controlled effectiveness study	Intervention: OFSP vine, distribution, nutrition education, and marketing*1*) Low-intensity intervention: 1 y training* 2*) High-intensity intervention: 3 y training* 3*) Control	Pathways not reported; Impact in children and women↑ OFSP consumption in both, ↑ vitamin A intake from OFSP in interventions; lower change in Niacin intake in group 2 among children aged 3–5.5 y; No impact on children's intake of protein, lipid, calcium, iron, zinc, vitamin C, thiamin, riboflavin, or vitamin B-12	X		Medium
			↓ Prevalence of inadequate vitamin A intake in children (aged 12–35 mo) and women			
Hotz et al. (70); Uganda	Randomized controlled effectiveness study	*1*) Intervention 1: OFSP vines plus agriculture, nutrition, and health BCC for 2 y (IP)* 2*) Intervention 2: OFSP vines for 2 y, nutrition and health BCC in year 1 only (RP)*3*) Control	Pathways not reported↑ OFSP and vitamin A intake in IP and RP↓ Prevalence of inadequate vitamin A for children 6–35 mo old; No impact on prevalence of infection based on elevated CRP for children 3–5 y old or women (IP vs. control); ↓ prevalence of low serum retinol for children in IP; no impact on serum retinol for women; ↓ prevalence of inadequate vitamin A for women, no difference in treatment groups	X		Medium
Jones and de Brauw (71); Mozambique	Cluster RCT	*1*) Intervention: OFSP inputs (vines) and training; demand creation on OFSP, general health messages; and marketing* 2*) Control	Pathways not reported; Impact in children↓ Prevalence of diarrhea among children; children eating OFSP were less likely to experience diarrhea;↓ Duration of diarrhea among children aged <3 y	X		Medium
Low et al. (36); Mozambique	Quasi-experimental prospective longitudinal	*1*) Interventions OFSP production, storage, and commercialization, Nutrition and hygiene education, *2*) Control	↑ Production and selling of sweet potato↑ Knowledge on nutrition, more for intervention women and menImpact in children:↑ Consumption of OFSP, and intakes of vitamin A, energy, protein, β-carotene, thiamin, riboflavin, vitamin B-6, vitamin C, folate, iron; no change in zinc, calcium intake↑ Serum retinol ↓ prevalence of low serum retinol; no impact on CRP ↓Prevalence of wasting and underweight; no impact on stunting	X	X	Low
Low et al. (46); Mozambique	Quasi-experimental intervention design	*1*) Interventions OFSP production, storage, and commercializationNutrition and hygiene education, *2*) Control	↑ OFSP production, preservation, storage, and processing↑ OFSP selling, ↑ OFSP purchase, mixed impact on expenditure;↑ Job opportunities, highest net returns to labor↑ Nutritional knowledge on vitamin A↑ Consumption of OFSP, fruits, dark GLVs, groundnuts, beans in children; no change in consumption of chicken, fresh fish;↑ Intakes of vitamin A, energy, β-carotene, riboflavin, vitamin B-6, vitamin C, thiamin, niacin, folate, and iron in children; no impact on intakes of vitamin B-12, calcium, and zinc in children↑ Dietary diversity in children; diet diversification was limited by difficult agroecological conditions and low purchasing power	X	X	High
Kassa et al. (72); Ethiopia	Cross-sectional surveys (2 times) and qualitative methods	Dairy goat husbandry training, revolving credit, *1*) crossbred goat; *2*) local goat; and *3*) controls, animal HW support, and nutrition education	↑ Ownership of goat; no impact on sheep or chicken Adults: ↑milk consumption, less milk available for children; no impact on consumption of vitamin A–rich ASFs↓ Stunting and underweight in children; no impact on wasting		X	High
Le Port et al. (50); Senegal	Cluster RCT	*1*) Intervention: iron fortification (yogurt value chain); ENA BCC, home visits, social mobilization*2*) Control: BCC	↑ Knowledge on health consequences of anemia and dietary sources of iron↑ Hb concentration in children, more in intervention group, greater impact for boys, compared to girls No impact on prevalence of anemia or severe anemia	X	X	Medium
Kerr et al. (73); Malawi	Quasi-experimental design	Interventions: legume intercrops, gender and nutrition education sessions; Control	Pathways not reported↑ WAZ score for longest-involved villages and most intensely involved villages	X		High
Kerr et al. (74); Malawi	Qualitative quasi-experimental design	Agricultural and nutrition education, legume intercrops, gender and nutrition education (participatory education)	↑ Knowledge on nutrition and health Women empowerment: ↑ resource access, gender norms;↑ Consumption of variety of foods in children		X	Medium
Angeles-Agdeppa et al. (49); Philippines	Cluster RCT	*1*) School 1. Iron-fortified rice supplementation*2*) School 2. Nonfortified rice supplementation; Both schools: gardening, health, hygiene and nutrition education, meal planning, preparation, and cooking	↑ Types and quantity of vegetables used in feeding; HHs purchase fortified foods;↑ Postexam lessons on nutrition, food fortification, hygiene, and health; no impact on the knowledge of foods needed by schoolchildren↑ Attitude on meal preparation and cooking vegetables Knowledge and practices of the mothers associated with children's weight↑ Mean Hb concentration in school 1; ↓ prevalence of anemia in school 1↓ Underweight (school 2) and stunting (school 1); no impact on wasting Mothers: ↑ Purchase of fortified foods, ↑ practice of washing vegetables before use	X	X	Medium
Erismann et al. (58); Burkina Faso	Cluster RCT	*1*) Intervention: agriculture inputs and training; WASH facilities; hygiene and nutrition training; treatment of anemia or intestinal infections*2*) Control: treatment of anemia or intestinal infections	Pathways not reported;↑ Safe handwashing practices before eating, and ↑ Use of latrines at school No impact on HH water quality parameter;↓ Intestinal parasitic infections but no impact on helminth infections No impact on anemia reduction, weight gain, or height gain	X		Medium
Schreinemachers et al. (51); Nepal	Cluster RCT	*1*) Intervention: school garden, curriculum on gardening, nutrition, and WASH, nutrition promotion and handwashing*2*) Control	↑ Knowledge on sustainable agriculture, food, nutrition and WASH↑ Preference of fruits and vegetablesNo impact on consumption of fruits and vegetables, or HAZ score	X	X	Medium
Schreinemachers et al. (53); Bhutan	RCT	*1*) Intervention schools: school garden; BCC/education on agriculture, nutrition, and WASH; promotional* 2*) Control	↑ Awareness on sustainable agriculture and percentage of fruits and vegetables correctly named; no impact on knowledge on food, nutrition, or WASH; ↑ preference of fruits and vegetablesSelling of garden produce to school meal programChildren: ↑ consumption of vegetables; no impact on consumption of fruit; ↑ consumption of variety of vegetables and fruit for children with home gardenNo impact on HAZ score	X	X	Medium
Gelli et al. (38); Malawi	RCT	*1*) Intervention: agriculture, nutrition, ECD Agriculture: foods production and diversification; CBCC gardens; food processing; seeds and chicks provisionNutrition: meal preparation and provision, feeding young children*2*) Control: ECD only	↑ Crop production variety/diversity, ↑production of OFSP, soya beans, pigeon peas, groundnuts; reduced production of brown beans,↑ Production of eggs and ownership of chickens No effects on household expenditures;↑ Caregiver's broad IYCF knowledge; ↑ food groups knowledge score↑ Number of days CBCC open and number of meal-providing days in CBCC; marginal ↑ CBCC enrolment; no impact on attendance;↑ Dietary intakes of food quantity, energy, protein, iron, zinc, vitamin C, vitamin B-6, vitamin B-12; no impact on dietary intake of vitamin A/RAE↑ Dietary diversity and food variety	X	X	Medium
			↓ Stunting among younger siblings aged 6–24 mo, no impact in preschools (36–72 mo); no impact on underweight, WAZ score, wasting, or WHZ score			
Roche et al. (75); Ethiopia	Qualitative design	Establish local grain bank: processing techniques, home fortification, complementary food-entry point for IYCN (mobilization of health workers and women's groups)	Production of processed grain bank flour, income, purchase of complementary food Mother's knowledge on complementary feeding, mother's perception on the benefit of grain bank food, and grandmother's perception on the importance of IYCF Time saving for women Flour consumption by pregnant and lactating women, elderly, and sick persons Complementary feeding practices Perceived decrease in prevalence of malnutrition		X	Medium
Kalavathi et al. (76); India	Repeated cross-sectional (before and after project)	Financial support (intercropping, nutrition gardening, nursery establishment, livestock rearing, azolla cultivation)Training on the production of high-value products (infant foods and other nutritional foods from locally available raw materials)	↑ Homestead backyard gardening, livestock/fish/poultry rearing↑ Income from intercrops, livestock, and processing, poverty reduction↑ Food processing, ↑ food security↑ Consumption of fish/ meat, vegetables, fruits, milk (products), fruits, Perceived ↑ nutrition security (not based on growth measurement) ↑ Egg consumption in children↑ Dietary diversity of children and adults		X	High
Fanzo et al. (77); Kenya	Cohort	Food and livelihood: livestock; fish; homegrown school meal; school garden; food processing, storage, and cooking; IYCF promotion; CMAM; school nutrition (e.g., deworming); growth monitoring; vitamin A, IFA, and zinc supplement; treatment of SAM; institutional delivery	Pathways not reported↓ Food insecurity↑ Daily consumption of vitamin A–rich animal product; no impact on vitamin A-rich plant products↑ Food variety score per week, number of daily meals, and mean DDSNo impact on vitamin A supplementation in children↓ VAD in children <5 y old↓ Stunting and underweight in children aged <2 y, no impact on wasting No impact on VAD in women aged 13–49 y	X		High

1 *n* = 43 individual studies. AGP, α-1-acid glycoprotein; ARI, acute respiratory infection; ASF, animal source food; BCC, behavior change communication; ben., beneficiary; BF, breastfeeding; CBCC, community-based childcare center; CDD, children's dietary diversity; CMAM, childhood management of acute malnutrition; CRP, C-reactive protein; DDS, dietary diversity score; DR, Democratic Republic; EBF, exclusive breastfeeding; ECD, early child development; EHFP, enhanced homestead food production; F2F, farmer to farmer training; FCHV, female community health volunteer; FFS, farmer field school; FP, family planning; GLV, green leafy vegetable; HAZ, height-for-age *z* score; Hb, hemoglobin; HC, health center; HDDS, household dietary diversity score; HFIAS, household food insecurity access scale; HFP, homestead food production; HG, home garden; HH, household; HW, health worker; IEC; information, education and communication; IFA, iron–folic acid; IP, intense participation; IYCF, infant and young child feeding; IYCN, infant and young child nutrition; KG, kitchen garden; LAZ, length-for-age *z* score; MAD, minimum acceptable diet; MAR, mean adequacy ratio; MDD, minimum dietary diversity; MMDA, minimum micronutrient density adequacy; MMF, minimum meal frequency; MNP, micronutrient powder; MUAC, midupper arm circumference; nonben., nonbeneficiary; NSA, nutrition-sensitive agriculture; OFSP, orange-fleshed sweet potato; OWL, old women leader; PM2A, preventing malnutrition in children under 2 approach; RAE, retinol activity equivalents; RBP, retinol-binding protein; RCT, randomized controlled trial; RP, reduced participation; SAM, severe acute malnutrition; VAD, vitamin A deficiency; WASH, water, sanitation, and hygiene; WAZ, weight-for-age *z* score; WDDS, women's dietary diversity score; WE, women's empowerment; WEG, women empowerment group; WHZ, weight-for-height *z* score; WLZ, weight-for-length *z* score; ↑, increase, positive difference; ↓, decrease, positive difference.

### Effects of NSA interventions on nutrition outcomes

The 37 studies included in the impact analysis reported 11 categories of interventions: HFP of vegetables and/or fruits and poultry ( *n* = 11); orange-fleshed sweet potato (OFSP) ( *n* = 8); HFP of vegetables and/or fruits, without poultry (*n* = 4); vegetable and livestock (*n* = 4); school garden (*n* = 4); livestock focusing on the dairy goat (*n* = 1); farm crop diversification (*n* = 1); HFP of fish and vegetables (*n* = 1); HFP of poultry (*n* = 1); food production using a community-based early child development (ECD) center ( *n* = 1); and mixed interventions on integrated food and livelihoods–based models, with nutrition-specific interventions and institutional delivery (*n* = 1). All interventions included education or behavior change communication (BCC) on nutrition, WASH, and/or health, with some including gender, health-service integration, and micronutrient supplementation.

The studies reported the effects of NSA interventions on outputs; their short-, medium-, and long-term outcomes; and impacts. The outputs were food production, knowledge of nutrition/health/WASH, and service delivery. These outputs contributed to the short-term outcomes on agricultural income, attitude/preference on nutrition, women's empowerment, and household living environment. The interventions increased nutrition-related expenditure, household food security, and care practices in the medium term, which resulted in long-term outcomes on dietary practices and diseases, eventually contributing to the impact on nutritional status. The effects follow, with further details provided in Table 2 and Supplemental Table 2.

### Outputs

The interventions reported on food production; knowledge on nutrition, WASH, and health; and increased service delivery through increased opening days of the local institution. Twelve of 14 studies reported improved production of ≥1 food item, but the effect varied across different food groups. The studies reported the increase in production of OFSP (3 of 3) (36–38), vegetables (5 of 8) (39–43), legumes, nuts, and pulses (2 of 3)(38, 39), fruits (2 of 4) (40, 42), and animal source foods (ASFs) (2 of 5) (38, 43). Thirteen of 14 studies reported improved knowledge of ≥1 topic related to nutrition, WASH, or health, with diverse effects. All studies reporting outcomes on knowledge of vitamin A (37, 44), maternal nutrition (45), general nutrition (36, 44, 46–49), and health and nutrition-related diseases (44, 45, 49, 50) reported improvements. At least half of the studies looking at children's knowledge of nutrition (1 of 2) (51), and WASH (3/4) (42, 49, 51) documented improvements. Knowledge of infant and young child feeding (IYCF) varied across specific topics, with 3 of 4 studies reporting improved knowledge on aspects of IYCF measured (38, 42, 52). Furthermore, an intervention delivered through a community-based childcare center increased the number of opening days of the center, with marginal improvement in the enrollment of children (38).

### Short-term outcomes

The interventions improved income, nutrition-related attitude and preferences, and women's empowerment. Three of 4 studies looking at the effect on income/selling showed improvements in food items, such as vegetables (2 of 3) (39, 40), OFSP (36), as well as a nonfood item (cottonseed) (39). Three studies reported changes in mothers’ attitudes on meal preparation, mothers’ ability to convince their children to eat vegetables (1 of 1) (49), and children's probability of consuming vegetables and fruits (2 of 2) (51, 53). Four studies also reported improvement in ≥1 domain of women's empowerment. Specifically, the interventions improved women's involvement in decision-making (3 of 4) (40, 54, 55) on purchasing (3 of 3) (40, 54, 55). However, interventions did not improve women's decision-making on health care (54, 55), family planning (54, 55), or IYCF (55), perception of gender equality, or control over selling (52). In addition, interventions improved women's agriculture empowerment score (1 of 1) (52), access to money or financial empowerment (1 of 1) (52), social status or social capital score (2 of 3) (40, 52), spousal communication and relationship (1 of 3) (54), and time allocation or self-determination of daily workload (1 of 1) (40). One study reported increased time allocation in agriculture, but decrease in time in domestic work and childcare practices, and buying power (52).

### Medium-term outcomes

In the medium term, the studies reported effects on household living environment, household food security, nutrition-related expenditure, and children's care practices. The impact on the household living environment was less strong, because only 1 of the 4 studies reported improved access to hygiene and sanitation facilities (56), with no impact on access to drinking water sources (56, 57) or water quality (58). The interventions improved household food security (3 of 5) (43, 62, 77), increased expenditure on food (3 of 6) (40, 49, 55) and health care (1 of 1) (40), and reduced expenses for vegetables owing to increased production (1 of 1) (47). The interventions further improved children's care practices and IYCF, especially breastfeeding and complementary feeding (2 of 5) (43, 45) and handwashing (2 of 2) (48, 58). However, studies lacked evidence regarding the effect on women's care practices, except for caregivers’ handwashing practices (1 of 1) (48).

### Long-term outcomes

NSA interventions had positive effects on long-term outcomes regarding dietary practices (food consumption, dietary diversity, and nutrient intake) and diseases, with less strong effects among women than among children. The interventions improved children's consumption of OFSP (4 of 4) (36, 46, 69, 70) and vegetables (3 of 7) (46, 53, 57), fruits (2 of 6) (46, 57), ASFs (4 of 8) (41, 56, 59, 63), and pulses, legumes, and nuts (3 of 4) (39, 46, 63). The studies reported improved household consumption of vegetables (3 of 7) (40, 41, 48), fruits (3 of 5) (40, 48, 55), ASFs (3 of 6) (45,55, 77) and pulses, legumes, and nuts (1 of 2) (39). The effect on consumption in women was reported for fruits (2 of 4) (44, 55), OFSP (2 of 2) (69, 70) vegetables (1 of 4) (44) or ASFs (2 of 4) (59, 63). The interventions also improved children's dietary diversity (9 of 13) (38, 43, 46, 56, 57, 61, 63, 65, 68), minimum acceptable diet (4 of 5) (43, 56, 57, 61), and minimum meal frequency (3 of 5) (43, 52, 61), followed by dietary diversity at the household level (3 of 6) (41, 62, 77), yet lacked a strong effect among women (0 of 5) (39, 41, 44, 55, 63). Likewise, the effect on nutrient intake was also stronger for children than for women. The interventions improved the nutrient adequacy ratio of children (1 of 2) (68), with one study reporting on women with no effect (63). In children, the interventions further increased intake of vitamin A (5 of 6) (36, 46, 68, 69, 70), iron (3 of 5) (36, 38, 46), vitamin B-6 (3 of 3) (36, 38, 46), zinc (1 of 5) (38), thiamin, and/or niacin (2 of 4) (36, 46), riboflavin (2 of 4) (36, 46), energy (3 of 4) (36, 38, 46), and protein (3 of 5) (36, 38, 46), with no change in calcium (36, 46, 59). Four studies reported nutrient intake for women, with improvements in vitamin A (3 of 3) (44, 69, 70) and β-carotene (1 of 1) (44), yet with no evidence of effect on the intake of energy (44, 59), iron, protein, calcium, zinc, thiamin, riboflavin, or niacin (59). In children, a few studies documented reductions in diarrhea (4 of 7) (42, 52, 66, 71), fever (1 of 4) (41), intestinal parasitic infections (58) or acute respiratory infections or colds/cough (1 of 2) (52). One study reported that children consuming OFSP were 15.9 percentage points less likely to experience diarrhea (71). Among women, one study reported no difference in the prevalence of diarrhea (41). Two studies reported mixed effects based on the combination of a third intervention component with agricultural production and nutrition-related education. An enhanced homestead food production (EHFP) intervention alone reduced diarrhea among children, whereas there was no effect after adding micronutrient powder (66). Further, a school-based intervention integrating installation of WASH facilities reduced intestinal parasitic infections but not the helminth infection rate (58).

### Impact

The impact of NSA interventions is less strong for nutritional status based on anthropometric measurements than for micronutrient status. Eight of 12 studies reported improvements in childhood micronutrient status (36, 42, 43, 49, 50, 67, 70, 77), by either increasing hemoglobin (3 of 8) (42, 49, 50) or reducing anemia (4 of 8) (42, 43, 49, 67), low serum retinol or vitamin A deficiency (3 of 3) (36, 70, 77). For anthropometric indexes among children, 7 of 21 studies reported improvements in nutritional status (36, 38, 45, 49, 65, 73, 77). The highest number of studies reported reductions in underweight/weight-for-age *z* scores (6 of 16) (36, 45, 49, 65, 73, 77), followed by stunting/ height-for-age *z*scores (4 of 17) (38, 49, 65, 77) and wasting/ weight-for-height *z* scores (1 of 15) (36). Among women, the interventions reduced anemia (1 of 3) (43) and inadequate vitamin A (4 of 5) (44, 67, 69, 70) but lacked effects on hemoglobin concentrations (41, 44, 67). Further, studies reported a reduction in underweight or improved BMI among women (2 of 5) (43, 55), but had no impact on their midupper arm circumference (44).

As with long-term outcomes, impact on nutritional status varied according to the integration of intervention components. For example, adding micronutrient powder to EHFP marginally reduced anemia, but EHFP alone did not bring the change (66). An agriculture–gender intervention significantly improved the weight-for-height *z* score among children, whereas adding a BCC component did not result in the same effect (52). Likewise, an EHFP intervention alone largely reduced anemia among children, but adding a fish component did not bring the same effect (67).

### Factors contributing to the effect of NSA interventions on nutrition outcomes

Studies indicated 11 factors that influence the effect of NSA interventions on nutrition outcomes. These factors are program participation intensity, program duration, nutritional status of the target population at baseline, age and sex of children, access to roads, seasonality, agroecology, purchasing power, wealth status, and maternal education. The most intense program participation contributed to improved weight-for-age *z* score (73) and higher coefficient of dietary diversity (68). Villages with the longest program duration reported improved weight-for-age *z* score of children (73). Populations that were undernourished at baseline had the significant reduction of undernutrition, particularly regarding BMI in women (55) and stunting and underweight in children (49). Furthermore, the interventions reduced undernutrition more among young children than among older children. For example, studies reported reductions in stunting among children aged 6–24 mo (38), and inadequate vitamin A among children aged between 12 and 35 mo (70) with no effects on stunting among children 36–72 mo of age (38), or inadequate vitamin A in 3- to 5-y-old children (70). Similarly, gender played a part in the outcomes reviewed, with 1 study reporting higher hemoglobin status for boys than for girls (50). Further, access to roads affected selling, because a greater mean quantity of sweet potato was sold in households closer to the main road (46). Seasonality also affected outcomes: for example, increased expenditure during harvest season (46) and increased women's and children's dietary diversity in winter (64). Agroecology also influenced the effect, because an intervention improved child dietary diversity in winter in the plains, but had no effect in mountain regions (64). Low purchasing power also adversely affected dietary diversity (46), whereas maternal education and better wealth status had a positive impact on consumption of nutritious foods (57).

### Impact pathways

**FIGURE 3:**
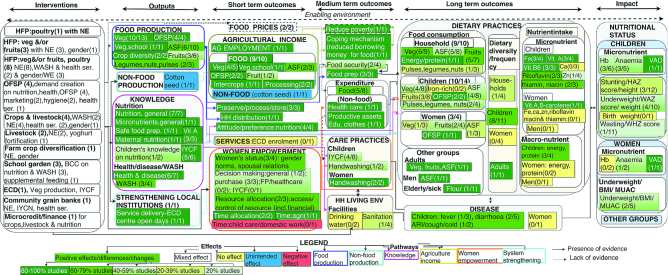
Impact pathways from NSA interventions to nutrition outcomes, as reported by studies included in pathways analysis, NSA impact pathways to nutrition outcomes (*n* = 29). ARI, acute respiratory infection; ASF, animal source food; BCC, behavior change communication; Ca, calcium; ECD, early child development; edu, education; Fe, iron; FP, family planning; HAZ, height-for-age *z* score; Hb, hemoglobin; HFP, homestead food production; HH, household; IYCF, infant and young child feeding; IYCN, infant and young child nutrition; MUAC, midupper arm circumference; NE, nutrition education; NSA, nutrition-sensitive agriculture; OFSP, orange-fleshed sweet potato; prep, preparation; ser, services; VAD, vitamin A deficiency; veg, vegetable; Vit., vitamin; WASH, water, sanitation, and hygiene; WAZ, weight-for-age *z* score, WHZ, weight-for-height *z* score; Zn, zinc.

The 29 studies included in the pathways analysis reported 5 pathways to nutrition outcomes from 11 categories of interventions. Most studies reported on HFP of vegetables and/or fruits and poultry (*n* = 8) followed by crops and livestock (*n* = 4), OFSP (*n* = 4), HFP of vegetables and/or fruits (*n* = 3), school garden (*n* = 3), livestock focused on goats (*n* = 2), farm crop diversification (*n* = 1), HFP of poultry (*n* = 1), food production using community-based ECD ( *n* = 1), community-based grain banks (*n* = 1), and microcredit/financial support (*n* = 1). The interventions integrated nutrition education (*n* = 29), WASH education (*n* = 9), linkages with health services (*n* = 4), gender components (*n* = 6), and micronutrient fortification/supplementation (*n* = 3). The studies reported 5 pathways from agricultural production (*n* = 21) ( 35–43, 45–49, 56, 57, 59, 60, 72, 75, 76); agricultural income (*n* = 9) ( 35, 36, 39, 40, 45, 46, 53, 75, 76); knowledge of nutrition, health, and/or WASH (*n* = 17) (35–38, 42, 44–53, 74, 75); women's empowerment (*n* = 6) (40, 52, 54, 55, 74, 75); and strengthening of local institutions (*n* = 1) (38). However, multiple combinations of these were often reported within a single study. Most studies reported on 2 entry points to the pathways (*n* = 11) (production and knowledge, production and income, knowledge and women's empowerment, and knowledge and income), followed by single (*n* = 11), 3 (*n* = 6), and 4 entry points (*n* = 1). **Figure 3** shows the pathways, with further details presented in Table 2, **Supplemental Figure 1**, and Supplemental Table 2.

#### Production pathway

Fifteen studies reporting improved food production also noted improved dietary practices through greater food consumption, dietary diversity, or nutrient intake (15 of 17) (35–41, 43, 45, 46, 48, 59, 60, 75, 76). Of these, 1 study reported an association between greater vegetable production and improved dietary diversity (41). Food production contributed to food consumption through preservation, processing, and storage (3 of 3) (46, 48, 76); household preparation of food (3 of 3) (45, 48, 49); and household distribution of food products (1 of 1) (53).

#### Income pathway

Five of 9 studies that looked at agricultural sales or income reported on expenditure (35, 40, 46, 48, 75). Of these, 4 increased food-related expenditure (35, 40, 48, 75) including purchases by nonintervention households (1 of 1) (48) and purchasing eggs from the market (1 of 1) (35). One study also reported increased expenditure on health care, education, clothes, and productive assets (40). Income also translated into a reduced need to borrow money for food, a common coping mechanism for food-insecure persons in LMICs (1 of 1) (76).

#### Knowledge pathway

Most of the interventions improving knowledge on nutrition, WASH, or health also contributed to improved dietary practices and, sometimes, improved care practices. Eleven of 15 studies reporting an increase of knowledge also recorded an improvement in ≥1 dietary practice (35–38, 44–46, 48, 52, 74, 75) and 4 reported improved children's care practices on IYCF (4 of 5) (35, 45, 48, 75) or handwashing (2 of 3) (48, 45). Five studies specifically looked at the contribution of a knowledge pathway on dietary practices (56, 52), diarrhea (52, 42), or nutritional status (49, 50). Of these, 3 studies reported that adding nutrition education/BCC improved children's dietary diversity (56) or minimum meal frequency (52), or reduced diarrhea among children (42, 52). One study reported improvement in hemoglobin, and reduction in diarrhea and anemia among children when BCC was delivered by health-center members (rather than older women leaders), highlighting the role of BCC provider (42). Two studies reported on the pathway to nutritional status. First, a study that compared BCC with the combination of micronutrient fortification and BCC in a dairy value chain program found improved micronutrient status in both groups, asserting that improved nutritional status may be the result of knowledge obtained from BCC (50). The second study revealed an association between nutrition-related knowledge among mothers and weight of their children (49).

#### Women's empowerment pathway

Six of 29 studies that reported on gender interventions also described elements of women's empowerment (40,52, 54, 55, 74, 75), of which 3 studies reported 2 subpathways. The first subpathway emerged from intrahousehold decision making and resource allocation. A study revealed a 1.9 of the overall 7.5 percentage points reduction in wasting attributable to women's empowerment owing to spousal communication as well as decision making on purchases, health care, and family planning (54). The second study, which revealed increased weight-for-height *z* score in the agriculture–gender group, also reported improved women's financial empowerment, access to assets, and agricultural empowerment (52) with no effect on decision making, sale of assets, or spousal relationship (52). The third study, using a qualitative approach, reported a case wherein a respondent (male) had knowledge about undernutrition and was involved in children's caring practices that led to greater variety of food consumption. However, the study did not make explicit whether the change was because of the change in knowledge or gender norms or both (74). The second subpathway was centered on women's time and appeared through a trade-off, wherein women spent more time in agriculture, leaving less time for domestic work and childcare practices (52). Although 4 studies reported an increase in self-determination among women in terms of prioritizing daily workload (40), financial empowerment (52) or decision-making on purchasing (40, 54, 55), only one assessed nutrition outcomes among women which reported reductions in underweight (55).

#### Strengthening existing institutions pathway

Out of the 10 studies that reported on interventions involving local institutions on health, agriculture, education, and ECD (35, 38, 42–44, 51–53, 56, 75), 3 hypothesized that strengthening service delivery would contribute toward nutrition outcomes (35, 38, 44). One study reported lack of adherence to program design and inadequate qualification and motivation of staff, resulting in weaknesses in service delivery (35). These studies suggested the improved delivery of NSA interventions through local institutions—e.g., ECD and health care service centers (38, 44)—and recommended strengthening program implementation and promoting higher participation to improve child nutrition outcomes (52). One study reported the effect on stages of the pathway (38) and showed increases in the number of opening days of a community-based childcare center and the number of meals (38) offered by it. The intervention also increased dietary diversity and reduced stunting among children aged 6–24 mo (38).

Findings on the pathways from dietary diversity to nutritional status are conflicting. A study integrating crops and livestock reported no significant association between children's dietary diversity and mean height-for-age *z* scores (56). An HFP intervention improved children's minimum dietary diversity and reduced anemia, but did not have impact on child growth (43). Likewise, only one of the 4 studies reporting improved child dietary diversity improved the anthropometric measurements (38). The evidence on women is even scarcer, because only 1 study measured both dietary diversity and undernutrition. This research revealed reductions in underweight among women despite marginal improvements in dietary diversity (55). As such, the link through which greater dietary diversity may consequently improve nutritional status is still unclear.

## Discussion

### Effects of NSA interventions on nutrition outcomes

The 37 studies on the impact of NSA interventions on nutrition outcomes concerning undernutrition indicate that these interventions have the potential to address multiple underlying determinants of undernutrition, yet have a weaker impact on nutritional status. In line with the recommendation from a previous review that indicated the potential role of NSA in addressing determinants of undernutrition beyond food access, we reviewed NSA interventions using a system approach, encompassing all underlying causes of malnutrition (78), namely *1*) household food insecurity, *2*) inadequate care practices, and *3*) unhealthy household environments and insufficient health services (7). The key effects of the interventions revolved around household food security, nutrition-related expenditure, nutrition-related knowledge, and women's empowerment, with a lesser but potential contribution on household living environment and children's care practices on IYCF and handwashing, and no evidence on women's care practices. These contributed to improved dietary practices and, to some extent, prevention of diseases among children but not among women. Disease prevention could be linked to improvement in handwashing and sanitation practices resulting from the integration of a WASH component, and one-fourth of stunting among children aged 2 y or younger is attributable to diarrhea (79). This indicates that NSA interventions have the potential to address multiple underlying causes of undernutrition. The effects of NSA interventions are strong for short-, medium-, and long-term outcomes, but with a disconnect between long-term outcomes and impacts on nutritional status as measured in terms of underweight, stunting, and wasting.

Our study confirms the weak impact of NSA interventions on nutritional status, with the lowest impact on stunting and wasting. Past studies on agriculture interventions also reported weak impact (19, 22, 25). This can be attributed to 3 possible reasons. First, underlying causes beyond food access are inadequately addressed. An earlier study highlighted the fact that agricultural programs that integrate multiple interventions can address a large number of immediate and underlying causes of child undernutrition (16) through coordination with multiple sectors including education, health, social safety nets, ECD, and schooling (6). Because only a few studies in this review considered underlying causes of undernutrition beyond food access, such as inadequate care practices and poor health status, the effect may not be enough to improve nutritional status. The second reason for lower impact on nutritional status could be that the short implementation period of interventions is insufficient to bring changes in stunting, despite visible effects on dietary practices (16). Among the studies included in this review, only 1 measured the effect of participation intensity and program duration on weight-for-age *z* score, which found a positive correlation, suggesting further validation research is needed. The third reason could be a lack of strong research methods, because designs with inadequate power might fail to detect changes in growth measurements (16, 17, 22).

The evidence reviewed regarding integrating intervention components beyond agricultural production and nutrition-related education is varied. Past reviews have suggested the need to make the agriculture sector nutrition-sensitive through a multisectoral approach (14, 16). Active engagement of multiple stakeholders and sectors (16) and attention to empowerment of women can improve nutrition outcomes, especially for women and children (14). The majority of studies reviewed measured the effect of agriculture combined with a nutrition-related education component, and found a positive effect on nutrition outcomes. Some studies also linked agricultural production to other sectors, such as ECD, nutrition-specific programs, financial support, and health services. However, the evidence on the effect of adding intervention components beyond food production and knowledge is heterogeneous, because the effect varies for different combinations. It could be the case that when there is a significant effect produced by 1 intervention component, the scope for improvements from other intervention components is reduced (80). Evidence on how to operationalize the right mix of intervention components in different contexts is overlooked, however, as also indicated by a past review (81). This calls for further research on which of the multisectoral components can be best combined within agriculture interventions and how to achieve optimal outcomes.

### Impact pathways

NSA interventions improve nutrition outcomes through 5 pathways: food production contributing to food access; knowledge on nutrition, WASH, and health improving dietary practices and health status; agricultural income for nutrition-related expenditure; women's empowerment contributing to nutrition outcomes in children; and strengthening of local institutions to enhance service delivery. The framework that we adapted does not explicitly mention the pathway of knowledge and strengthening local institutions (13) that emerged from our analysis of the studies reviewed. Past reviews have highlighted food price as a potential pathway (6, 12–14), but none of the studies reported on this, perhaps because food price has traditionally been considered at the policy rather than intervention level.

Although evidence on production is most dominant, the majority of studies reported on combinations of >1 entry point to the pathways to nutrition outcomes. Thus, the nutrition outcomes reported in this review should be considered to reflect the combined effects of multiple pathways that interact with each other to achieve nutrition outcomes (12). The evidence reviewed suggests that stimulating a combination of these impact pathways would result in the most significant effects on nutrition outcomes. Because there is now consensus that agriculture can contribute to addressing both forms of malnutrition (82), NSA can potentially contribute to addressing both forms of malnutrition as well. Nevertheless, this review explicitly focuses on undernutrition, and hence excludes the outcomes on obesity or overweight that some past studies have explored (83, 84).

Most studies reported on the production pathway, followed by knowledge. The production pathway, however, differs across food items, thus necessitating careful design and implementation across products. Many recent studies have begun to consider a knowledge pathway, as they tend to integrate nutrition education and BCC activities with agricultural production interventions. A previous review also highlighted integration of nutrition-related BCC as a key strategy to enhance the impact of agriculture on nutrition outcomes (16). Therefore, the pathway leading from the knowledge-based behavior change component should be considered an essential part of the design, implementation, and evaluation of NSA interventions.

Our review also confirms the pathways from agricultural income and women's empowerment, although these are less evident than the production and knowledge pathways. Interventions can contribute to food-related expenses through an income improved by selling food products. However, the evidence base lacks the role of income from nonfood production or agricultural wages, and the contribution to health care expenses. The studies reviewed highlighted 2 subpathways on women's empowerment contributing to nutrition outcomes: women's social status, decision-making, and resource allocation; and women's time in agriculture, of which the former is less evident. Trade-offs occur, because an increase in the time allocated to agriculture appears to mean less time for domestic work and childcare practices, and therefore calls for measures safeguarding women's time in agriculture (16) should ensure that such interventions do not contribute to an increased time and labor burden (12). However, as highlighted by other studies, this pathway is less evident in research of NSA interventions (13, 14): specifically, the contribution of women's empowerment to their own care practices and nutritional status is less evident. Most of the studies reporting gender in their interventions examined the effects on women's empowerment without describing how the interventions influenced the underpinning gender dynamics that empowered women and consequently led to nutrition outcomes. Some other studies on underlying gender dynamics and NSA were excluded from this review because they did not report on nutrition outcomes (85, 86).

One study we reviewed provided evidence on the temporal stages of the pathway to improved nutrition outcomes, through the strengthening of local institutions, and 2 other studies recommended mobilizing the institutions to improve implementation and service delivery (52, 44). Integrating nutrition into agriculture, however, requires the establishment and strengthening of an enabling institutional environment conducive to achieving nutrition objectives (87). This necessitates integrating nutrition into all elements of food systems, from food production to utilization (15, 16), but also requires an understanding of implementation quality to design pathways, and to measure implementation and service delivery (16). Future research could therefore apply a combination of impact assessment along with process evaluations investigating implementation quality in terms of capacity, resources, supportive environment, and potential for scaling up the interventions.

Addressing undernutrition through NSA interventions requires careful design, implementation, and evaluation considering several factors. The factors are types of food group, program participation intensity, program duration, nutritional status of the target population at baseline, children's age, children's gender, access to roads, seasonality, agroecology, purchasing power, wealth status, and maternal education. More efforts are required to address the confirmed factors, for example, undernutrition in children aged 3–5 y, seasonality, and mountainous areas. In addition, multisectoral interventions are required to improve purchasing power, wealth status, women's education, and access to roads. Factors with mixed results, such as program intensity and duration, should be further studied. There is also a need to study success and failure factors within NSA interventions, as well as external barriers and facilitators to achieving positive effects. We can thus say that NSA interventions to address undernutrition require a tailor-made approach to fit the specific context and the needs of the target population (23, 64).

### Strengths and limitations

Two aspects of this review that distinguish it from similar reviews and the studies included are an explicit focus on agricultural interventions with specific nutrition objectives and actions to achieve these objectives; and construction of temporal stages of their pathways to nutrition outcomes. Inclusion of studies reporting agriculture interventions with nutrition objectives and actions, however, does not imply that other interventions do not improve nutrition outcomes. Four limitations may have affected our findings. First, several studies reported on effects on outcomes without providing information on entry points to the pathways, such as food production, knowledge, or income. This limited the construction of pathways representing all studies included. To address this, we further selected and mapped a subset of studies reporting on both effects and the pathways. Nevertheless, a lack of information should be understood as a lack of evidence, and not the absence of pathways. The second limitation is the fragmentation of research findings regarding the same intervention across different articles: we identified studies reporting on the same interventions to the best of our ability. Third, the results should be carefully interpreted owing to the heterogeneity of study design, indicators used, and methodological quality. For this reason, we assessed the risk of bias to facilitate interpretation of findings. It should be noted that the risk ratings only indicate the methodological rigor through which the findings were produced, and are not meant to weigh the studies as a whole. Furthermore, the majority of these studies have a moderate or high risk of bias, which might be due to the nature of nutrition interventions being implemented in communities, where it is difficult to fully control the studies through randomization and blinding. Fourth, we did not search the gray literature, which could have provided additional relevant, unpublished articles on the same topic.

### Conclusions

Although current evidence suggests that NSA interventions can contribute to nutrition outcomes throughout the short-, medium-, and long-term temporal stages, there is a disconnect between long-term outcomes and impact on nutritional status based on anthropometric measurements. The increasing volume of publications on NSA interventions testifies to their potential to improve food access, but indicates that they can also address other underlying causes of undernutrition, namely unhealthy household environments and inadequate care practices. These outcomes are achieved through 5 main pathways: food production, agricultural income, nutrition-related knowledge, women's empowerment, and strengthening of local institutions. The impact pathways, however, vary across the type of food group consumed, agroecology, seasonality, access to roads, age and gender of children, wealth status, women's education, program intensity, program duration, and integration of multisectoral domains. Reconciling this complex mix of factors requires tailor-made interventions that are cognizant of barriers and facilitators to achieving their impacts. Further research is required to better describe the pathways through which women's empowerment can contribute to women's own nutritional outcomes, as well as the effect of income from nonfood production and agricultural work, food-price changes at intervention level, and strengthening of local institutions. Further research is also required on the impact of integrating other multisectoral intervention components within agriculture production and nutrition-, WASH-, or health-based education. In addition to targeting children, NSA intervention research should also focus on the impact on, and the pathways to, improved women's nutrition outcomes, to contribute toward addressing undernutrition in LMICs.

## Supplementary Material

nmaa103_Supplemental_Files

## Notes

This systematic review is part of the first and second authors’ PhD research; IKS is supported by the Erasmus Mundus Joint Doctorate Programme and SDP by the Netherlands Organization for Scientific Research.

Author disclosures: The authors report no conflicts of interest.

The funding agencies had no role in study design, execution, analysis, or preparation of the manuscript.

Supplemental Tables 1 and 2 and Supplemental Figure 1 are available from the “Supplementary data” link in the online posting of the article and from the same link in the online table of contents at https://academic.oup.com/advances/.

Abbreviations used: ASF, animal source food; BCC, behavior change communication; ECD, early child development; EHFP, enhanced homestead food production; HFP, homestead food production; IYCF, infant and young child feeding; LMICs, low- and lower-middle-income countries; NSA, nutrition-sensitive agriculture; OFSP, orange-fleshed sweet potato; WASH, water, sanitation, and hygiene.
