# Supplementary material for: Nutrition-Sensitive Agriculture: A Systematic Review of Impact Pathways to Nutrition Outcomes
Source: Adv Nutr. 2020 Sep 24;12(1):251–75. doi: 10.1093/advances/nmaa103 (PMC7850060; doi:10.1093/advances/nmaa103)
Supplement: nmaa103_Supplemental_Files [file nmaa103_supplemental_files.zip › Supplemental_Figure_1.Detailed_impact_pathways.pdf]

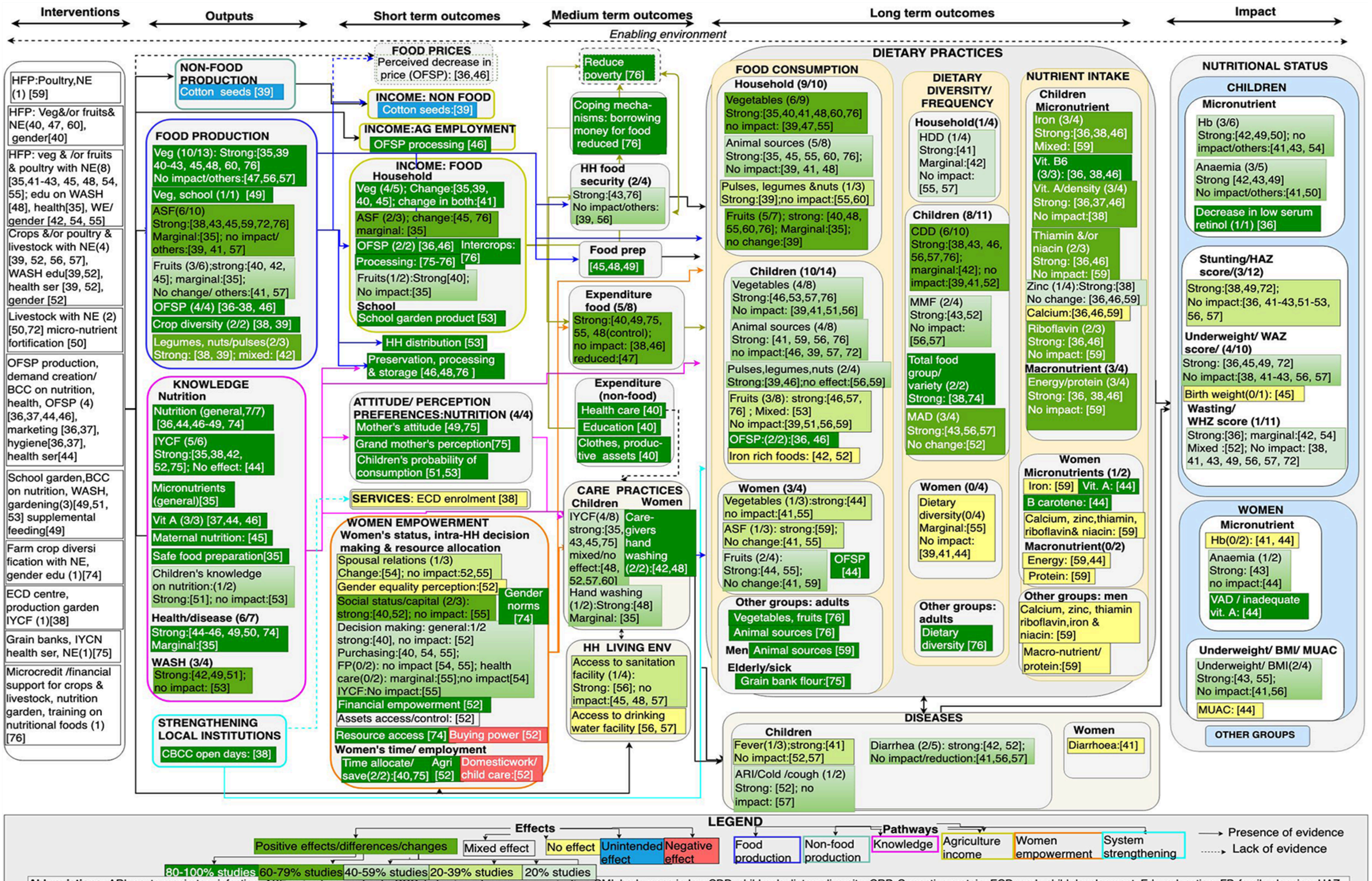

Abbreviations: ARI, acute respiratory infection; ASF, animal source foods; BCC, behaviour change communication; BMI, body mass index; CDD, children's dietary diversity; CRP, C-reactive protein; ECD, early child development; Edu, education; FP, family planning; HAZ, height-for-age z-score; Hb, haemoglobin; HDD, household dietary diversity; HFP, homestead food production; HH, household; IYCF, infant &young child feeding; IYCN, infant & young child nutrition; MAD, minimum acceptable diet; MMF, minimum meal frequency,MUAC, mid-upper arm circumference;NE, nutrition education; OFSP, orange-fleshed sweet potato; Prep, preparation; ser., services; VAD, vitamin A deficiency; Veg, vegetable; WASH, water, sanitation & hygiene; WAZ, weight -for-age z-score; WHZ, weight-for-height z-score
Note: the category "strong" includes statistically significant results (when available),quantitative effects reported with proportions and/or qualitative findings

***Supplemental Figure 1*. Detailed impact pathways from NSA interventions to nutrition outcomes (n=29)**
