# Supplementary material for: Nutrition-Sensitive Agriculture: A Systematic Review of Impact Pathways to Nutrition Outcomes
Source: Adv Nutr. 2020 Sep 24;12(1):251–75. doi: 10.1093/advances/nmaa103 (PMC7850060; doi:10.1093/advances/nmaa103)
Supplement: nmaa103_Supplemental_Files [file nmaa103_supplemental_files.zip › Supplemental_Table_1.Full_electronic_search_syntax.docx]

***Supplemental Table 1*. Full electronic search syntax applied in databases, NSA impact pathways to nutrition outcomes, with 20896 results**

Database 1. Full electronic search syntax applied in Embase on 25/03/2019, with 2595 results

| **Theme** | **Query No.** | **Query** | **Results** |
| --- | --- | --- | --- |
| Type of publication | #34 | #33 NOT (conference*:it OR letter*:it OR editorial*:it) | **2,595** |
| Publication year and English | #33 | #32 NOT ([animals]/lim NOT [humans]/lim) AND [2000-2018]/py AND [english]/lim | **3,426** |
| Agriculture AND Nutrition OR Nutrition Sensitive AND LMICs | #32 | #30 AND #31 | **4,406** |
| Agriculture AND Nutrition OR Nutrition Sensitive | #31 | #28 OR #29 | **24,799** |
| Low and lower middle-income countries  LMICs | #30 | 'focus countr*':ab,ti,kw OR 'low income':ab,ti,kw OR 'lower-middle income':ab,ti,kw OR 'developing countr*':ab,ti,kw OR 'south asia':ab,ti,kw OR 'south east asia':ab,ti,kw OR 'sub-saharan africa':ab,ti,kw OR 'subsaharan africa':ab,ti,kw OR oceania:ab,ti,kw OR pacific:ab,ti,kw OR afghanistan:ab,ti,kw OR guinea:ab,ti,kw OR rwanda:ab,ti,kw OR benin:ab,ti,kw OR 'guinea-bissau':ab,ti,kw OR senegal:ab,ti,kw OR 'burkina faso':ab,ti,kw OR haiti:ab,ti,kw OR 'sierra leone':ab,ti,kw OR burundi:ab,ti,kw OR korea:ab,ti,kw OR somalia:ab,ti,kw OR 'central african republic':ab,ti,kw OR liberia:ab,ti,kw OR 'south sudan':ab,ti,kw OR chad:ab,ti,kw OR madagascar:ab,ti,kw OR tanzania:ab,ti,kw OR comoros:ab,ti,kw OR malawi:ab,ti,kw OR togo:ab,ti,kw OR congo:ab,ti,kw OR mali:ab,ti,kw OR uganda:ab,ti,kw OR eritrea:ab,ti,kw OR mozambique:ab,ti,kw OR zimbabwe:ab,ti,kw OR ethiopia:ab,ti,kw OR nepal:ab,ti,kw OR gambia:ab,ti,kw OR niger:ab,ti,kw OR angola:ab,ti,kw OR indonesia:ab,ti,kw OR philippines:ab,ti,kw OR armenia:ab,ti,kw OR jordan:ab,ti,kw OR 'sao tome':ab,ti,kw OR bangladesh:ab,ti,kw OR kenya:ab,ti,kw OR 'solomon islands':ab,ti,kw OR bhutan:ab,ti,kw OR kiribati:ab,ti,kw OR 'sri lanka':ab,ti,kw OR bolivia:ab,ti,kw OR kosovo:ab,ti,kw OR sudan:ab,ti,kw OR 'cabo verde':ab,ti,kw OR kyrgyz*:ab,ti,kw OR swaziland:ab,ti,kw OR cambodia:ab,ti,kw OR lao:ab,ti,kw OR laos:ab,ti,kw OR syria*:ab,ti,kw OR cameroon:ab,ti,kw OR lesotho:ab,ti,kw OR tajikistan:ab,ti,kw OR mauritania:ab,ti,kw OR 'timor-leste':ab,ti,kw OR 'cote d ivoire':ab,ti,kw OR 'ivory coast':ab,ti,kw OR micronesia:ab,ti,kw OR tunisia:ab,ti,kw OR djibouti:ab,ti,kw OR moldova:ab,ti,kw OR ukraine:ab,ti,kw OR egypt:ab,ti,kw OR mongolia:ab,ti,kw OR uzbekistan:ab,ti,kw OR 'el salvador':ab,ti,kw OR morocco:ab,ti,kw OR vanuatu:ab,ti,kw OR georgia:ab,ti,kw OR myanmar:ab,ti,kw OR burma:ab,ti,kw OR vietnam:ab,ti,kw OR 'viet nam':ab,ti,kw OR ghana:ab,ti,kw OR nicaragua:ab,ti,kw OR 'west bank':ab,ti,kw OR gaza:ab,ti,kw OR guatemala:ab,ti,kw OR nigeria:ab,ti,kw OR yemen:ab,ti,kw OR honduras:ab,ti,kw OR pakistan:ab,ti,kw OR zambia:ab,ti,kw OR india:ab,ti,kw OR 'papua new guinea':ab,ti,kw | **791,899** |
| Nutrition sensitive | #29 | 'nutrition-sensitiv*':ab,ti,kw | **220** |
| Agriculture AND Nutrition AND multisector | #28 | #20 AND #27 | **24,664** |
| Multisector all | #27 | #21 OR #22 OR #23 OR #24 OR #25 OR #26 | **2,286,330** |
| Social protection | #26 | 'social inclusion':ab,ti,kw OR 'social protection':ab,ti,kw OR 'social assistance':ab,ti,kw OR voucher:ab,ti,kw OR vouchers:ab,ti,kw OR transfer:ab,ti,kw OR transfers:ab,ti,kw OR 'social safety net':ab,ti,kw OR 'social safety nets':ab,ti,kw | **470,383** |
| Education | #25 | education:ab,ti,kw OR school*:ab,ti,kw OR 'early child development':ab,ti,kw | **821,032** |
| Natural resource | #24 | 'natural resource*':ab,ti,kw | **5,635** |
| Health | #23 | 'health service*':ab,ti,kw OR 'maternal health':ab,ti,kw OR 'child health':ab,ti,kw OR 'adolescent health':ab,ti,kw OR 'mental health':ab,ti,kw OR 'family planning':ab,ti,kw | **317,654** |
| Water, Sanitation and Hygiene (WASH) | #22 | sanitation:ab,ti,kw OR hygien*:ab,ti,kw OR 'drinking water':ab,ti,kw OR 'safe water':ab,ti,kw OR handwash*:ab,ti,kw OR 'hand wash*':ab,ti,kw OR 'open-defecation':ab,ti,kw | **160,844** |
| Multi sector general | #21 | sector*:ab,ti,kw OR multisector*:ab,ti,kw OR intersector*:ab,ti,kw OR integrat*:ab,ti,kw OR convergen*:ab,ti,kw | **685,833** |
| Agriculture AND Nutrition | #20 | #12 AND #19 | **192,630** |
| Nutrition total | #19 | #13 OR #14 OR #15 OR #16 OR #17 OR #18 | **2,788,649** |
| Food general | #18 | food*:ab,ti,kw OR fruit*:ab,ti,kw OR vegetable*:ab,ti,kw OR milk:ab,ti,kw OR meat:ab,ti,kw OR egg:ab,ti,kw OR eggs:ab,ti,kw OR nutrient*:ab,ti,kw | **988,490** |
| Diet general | #17 | diet:ab,ti,kw OR diets:ab,ti,kw OR dietar*:ab,ti,kw | **594,739** |
| Nutrition general | #16 | nutrition*:ab,ti,kw OR undernutrition*:ab,ti,kw OR malnutrition*:ab,ti,kw OR micronutri*:ab,ti,kw | **387,332** |
| Nutrition assessment expanded | #15 | 'nutritional assessment'/exp | **26,253** |
| Malnutrition expanded | #14 | 'malnutrition'/exp | **159,467** |
| Nutrition expanded | #13 | 'nutrition'/exp | **2,086,898** |
| Agriculture total | #12 | #1 OR #2 OR #3 OR #4 OR #5 OR #6 OR #7 OR #8 OR #9 OR #10 OR #11 | **437,908** |
| Aquaculture/fishery | #11 | aquacult*:ti,ab,kw OR 'aqua cult*':ti,ab,kw OR 'fish pond*':ti,ab,kw OR fishpond*:ti,ab,kw OR fishery:ti,ab,kw OR fisheries:ti,ab,kw OR fishfarm*:ti,ab,kw OR 'fish farm*':ti,ab,kw | **20,606** |
| Water/irrigation | #10 | 'water management':ti,ab,kw OR irrigation:ti,ab,kw | **33,234** |
| Livestock | #9 | livestock*:ti,ab,kw OR dair*:ti,ab,kw OR poultr*:ti,ab,kw | **104,067** |
| Farmer | #8 | farmer:ti,ab,kw OR farmers:ti,ab,kw | **22,258** |
| Harvest | #7 | 'harvest':ti,ab,kw | **28,388** |
| Homestead gardening | #6 | homestead:ti,ab,kw OR horticult*:ti,ab,kw OR garden*:ti,ab,kw | **16,027** |
| Fortification | #5 | fortif*:ti,ab,kw OR biofortif*:ti,ab,kw | **17,566** |
| Value chain | #4 | 'value chain*':ti,ab,kw OR 'value crop*':ti,ab,kw | **886** |
| Agriculture general | #3 | 'agri-cult*':ti,ab,kw | **4** |
| Agriculture general | #2 | agricult*:ti,ab,kw | **87,263** |
| Agriculture expanded | #1 | 'agriculture'/exp | **212,983** |

Database 2. Full electronic search syntax applied in PubMed on 25/03/2019, with 2789 results.

| **Theme** | **Query** **no.** | **Queries** | **Results** |
| --- | --- | --- | --- |
| Language Filter | 33 | # 32 Filters: English | 2,789 |
| Publication date filter | 32 | # 31 Filters: Publication date from 2000/01/01 to 2030/12/31 | 2,813 |
| Human subjects filter | 31 | (#30) NOT ("Animals"[Mesh] NOT "Humans"[Mesh])" | 3,815 |
| [Agriculture AND (Food OR Nutrition) AND multisector] AND LMICs | 30 | #28 AND #29 | 4,176 |
| Low and lower middle-income countries (LMICs) | 29 | (low income[tiab] OR lower-middle income[tiab] OR developing countr*[tiab] OR focus countr*[tiab] OR Sub-saharan Africa[tw] OR Subsaharan Africa[tw] OR South Asia[tw] OR South East Asia[tw] OR Oceania[tw] OR Pacific[tw]) OR Afghanistan[tw] OR Angola[tw] OR Armenia[tw] OR Bangladesh[tw] OR Benin[tw] OR Bolivia[tw] OR Burkina Faso[tw] OR Burma[tw] OR Burundi[tw] OR Bhutan[tw] OR Cabo Verde[tw] OR Cambodia[tw] OR Cameroon[tw] OR Central African Republic[tw] OR Chad[tw] OR Comoros[tw] OR Congo[tw] OR Côte d’Ivoire[tw] OR Djibouti[tw] OR Egypt[tw] OR El Salvador[tw] OR Eritrea[tw] OR Ethiopia[tw] OR Gambia[tw] OR Gaza[tw] OR Georgia[tw] OR Ghana[tw] OR Guatemala[tw] OR Guinea[tw] OR Guinea-Bissau[tw] OR Haiti[tw] OR Honduras[tw] OR India[tw] OR Indonesia[tw] OR Ivory Coast[tw] OR Jordan[tw] OR Kenya[tw] OR Kiribati[tw] OR Korea[tw] OR Kosovo[tw] OR Kyrgyz*[tw] OR Lao[tw] OR Laos[tw] OR Lesotho[tw] OR Liberia[tw] OR Madagascar[tw] OR Malawi[tw] OR Mali[tw] OR Mauritania[tw] OR Micronesia[tw] OR Moldova[tw] OR Mongolia[tw] OR Morocco[tw] OR Mozambique[tw] OR Myanmar[tw] OR Nepal[tw] OR Nicaragua[tw] OR Niger[tw] OR Nigeria[tw] OR Pakistan[tw] OR Papua New Guinea[tw] OR Philippines[tw] OR Rwanda[tw] OR Sao Tome[tw] OR Senegal[tw] OR Sierra Leone[tw] OR Solomon Islands[tw] OR Somalia[tw] OR Sudan[tw] OR South Sudan[tw] OR Sri Lanka[tw] OR Swaziland[tw] OR Syria*[tw] OR Tajikistan[tw] OR Tanzania[tw] OR Timor-Leste[tw] OR Togo[tw] OR Tunisia[tw] OR Vanuatu[tw] OR Vietnam[tw] OR Viet Nam[tw] OR Uganda[tw] OR Ukraine[tw] OR Uzbekistan[tw] OR West Bank[tw] OR Yemen[tw] OR Zambia[tw] OR Zimbabwe[tw])" | 867,920 |
| [Agriculture AND (Food OR Nutrition) AND multi-sectors] OR nutrition sensitive | 28 | #26 AND #27 | 21,651 |
| Nutrition sensitive | 27 | nutrition-sensitiv*[tiab]" | 142 |
| [Agriculture AND (Food OR Nutrition) AND multi-sectors] | 26 | #18 AND #25 | 21,555 |
| Multisector, total | 25 | ((((((((((sector*[Title/Abstract]) OR multisector*[Title/Abstract]) OR intersector*[Title/Abstract]) OR integrat*[Title/Abstract]) OR convergen*[Title/Abstract])) OR (((((((sanitation[Title/Abstract]) OR hygien*[Title/Abstract]) OR drinking water[Title/Abstract]) OR safe water[Title/Abstract]) OR handwash*[Title/Abstract]) OR hand wash*[Title/Abstract]) OR open-defecation[Title/Abstract])) OR ((((((health service*[Title/Abstract]) OR maternal health[Title/Abstract]) OR child health[Title/Abstract]) OR adolescent health[Title/Abstract]) OR mental health[Title/Abstract]) OR family planning[Title/Abstract])) OR (((education) OR school*) OR "early child development")) OR (((((((((social inclusion[Title/Abstract]) OR social protection[Title/Abstract]) OR social assistance[Title/Abstract]) OR voucher[Title/Abstract]) OR vouchers[Title/Abstract]) OR transfer[Title/Abstract]) OR transfers[Title/Abstract]) OR "social safety net"[Title/Abstract]) OR "social safety nets"[Title/Abstract])) OR natural resource*[Title/Abstract]" | 5,732,097 |
| Natural resource management | 24 | natural resource*[Title/Abstract]" | 6,093 |
| Social protection | 23 | ((((((((social inclusion [Title/Abstract]) OR social protection [Title/Abstract]) OR social assistance [Title/Abstract]) OR voucher[Title/Abstract]) OR vouchers[Title/Abstract]) OR transfer[Title/Abstract]) OR transfers[Title/Abstract]) OR "social safety net"[Title/Abstract]) OR "social safety nets"[Title/Abstract]" | 415,887 |
| Education | 22 | ((education) OR school*) OR "early child development"" | 4,740,350 |
| Health | 21 | (((((health service*[Title/Abstract]) OR maternal health [Title/Abstract]) OR child health[Title/Abstract]) OR adolescent health[Title/Abstract]) OR mental health[Title/Abstract]) OR family planning[Title/Abstract]" | 281,101 |
| Water, sanitation and hygiene (WASH) | 20 | ((((((sanitation [Title/Abstract]) OR hygien*[Title/Abstract]) OR drinking water[Title/Abstract]) OR safe water[Title/Abstract]) OR handwash*[Title/Abstract]) OR hand wash*[Title/Abstract]) OR open-defecation[Title/Abstract]" | 129,228 |
| Multisector general topic | 19 | ((((sector*[Title/Abstract]) OR multisector*[Title/Abstract]) OR intersector*[Title/Abstract]) OR integrat*[Title/Abstract]) OR convergen*[Title/Abstract]" | 574,808 |
| Agriculture AND Nutrition | 18 | #11 AND #17 | 73,271 |
| Nutrition total | 17 | (((((nutrition*[Title/Abstract]) OR undernutrition*[Title/Abstract]) OR malnutrition*[Title/Abstract]) OR micronutri*[Title/Abstract])) OR (((((food*[Title/Abstract]) OR diet [Title/Abstract]) OR dietar*[Title/Abstract]) OR diets [Title/Abstract]) OR nutrient*[Title/Abstract])" | 1,065,393 |
| Food, diet or nutrient | 16 | ((((food*[Title/Abstract]) OR diet [Title/Abstract]) OR dietar*[Title/Abstract]) OR diets [Title/Abstract]) OR nutrient*[Title/Abstract]" | 882,401 |
| Nutrition general topic | 15 | (((nutrition*[Title/Abstract]) OR undernutrition*[Title/Abstract]) OR malnutrition*[Title/Abstract]) OR micronutri*[Title/Abstract]" | 293,547 |
| Mesh heading on nutrition, food or diet | 14 | "Malnutrition"[Mesh]" | 116,748 |
|  | 13 | "Nutrition Assessment"[Mesh]" | 13,895 |
|  | 12 | ("Diet, Food, and Nutrition"[Mesh])" | 1,016,837 |
| Agriculture total | 11 | ((((((((("Agriculture"[Mesh]) OR ((agricult*[Title/Abstract]) OR agri-cult*[Title/Abstract])) OR ((farmer[Title/Abstract]) OR farmers[Title/Abstract])) OR harvest*[Title/Abstract]) OR ((((("homestead"[Title/Abstract]) OR horticult*[Title/Abstract]) OR garden*[Title/Abstract])))) OR (((livestock*[Title/Abstract]) OR dair*[Title/Abstract]) OR poultr*[Title/Abstract])) OR ((((((((aquacult*[Title/Abstract]) OR aqua cult*[Title/Abstract]) OR fishery[Title/Abstract]) OR fisheries[Title/Abstract]) OR fish pond*[Title/Abstract]) OR fishpond*[Title/Abstract]) OR fishfarm*[Title/Abstract]) OR fish farm*[Title/Abstract])) OR ((fortif*[Title/Abstract]) OR biofortif*[Title/Abstract])) OR ((value chain*[Title/Abstract]) OR value crop*[Title/Abstract])) OR ((water management[Title/Abstract]) OR irrigation[Title/Abstract])" | 373,348 |
| Irrigation/water | 10 | (water management [Title/Abstract]) OR irrigation [Title/Abstract]", | 26,175 |
| Value chain | 9 | (value chain*[Title/Abstract]) OR value crop*[Title/Abstract]" | 668 |
| Fortification | 8 | (fortif*[Title/Abstract]) OR biofortif*[Title/Abstract]" | 14,190 |
| Aquaculture /fishery | 7 | (((((((aquacult*[Title/Abstract]) OR aqua cult*[Title/Abstract]) OR fishery [Title/Abstract]) OR fisheries [Title/Abstract]) OR fish pond*[Title/Abstract]) OR fishpond*[Title/Abstract]) OR fishfarm*[Title/Abstract]) OR fish farm*[Title/Abstract]" | 17,321 |
| livestock | 6 | ((livestock*[Title/Abstract]) OR dair*[Title/Abstract]) OR poultr*[Title/Abstract]" | 92,551 |
| Homestead garden | 5 | (((("homestead"[Title/Abstract]) OR horticult*[Title/Abstract]) OR garden*[Title/Abstract]))" | 14,119 |
| Harvest | 4 | harvest*[Title/Abstract]" | 98,552 |
| farmer | 3 | (farmer [Title/Abstract]) OR farmers [Title/Abstract]" | 18,068 |
| Agriculture general search | 2 | (agricult*[Title/Abstract]) OR agri-cult*[Title/Abstract]" | 75,467 |
| Agriculture Mesh | 1 | "Agriculture"[Mesh]" | 87,652 |

Database 3. Full electronic search syntax applied in Scopus on 26/03/2019, with 7,112 results.

Used two syntax as follows:

Total search excluding Homestead Food Production (HFP)

Date of search: 26/03/ 2019, result: 6,715

| **Theme** | **Query no.** | **Query** | **Results** |
| --- | --- | --- | --- |
| Filter by document type  (Article, Book Chapter,  Reviews) | 32 | ( (  ( DOCTYPE ,  "ch" )  OR  LIMIT-TO ( DOCTYPE ,  "re" )  OR  LIMIT-TO ( DOCTYPE ,  "ar" ) ) OR  LIMIT-TO ( DOCTYPE ,  "ip" ) ) | 6,715 |
|  | 31 | ( ( TITLE-ABS-KEY ( "nutrition-sensitiv*" ) )  OR  ( ( ( ( ( TITLE-ABS-KEY ( agricult* )  OR  TITLE-ABS-KEY ( "agri-cult*" ) ) )  OR  #  2  OR  ( ( TITLE-ABS-KEY ( "value chain*" )  OR  TITLE-ABS-KEY ( "value crop*" ) ) )  OR  ( ( TITLE-ABS-KEY ( fortif* )  OR  TITLE-ABS-KEY ( biofortif* ) ) )  OR  ( ( TITLE-ABS-KEY ( "water management" )  OR  TITLE-ABS-KEY ( irrigation ) ) )  OR  ( ( TITLE-ABS-KEY ( aquacult* )  OR  TITLE-ABS-KEY ( "aqua cult*" )  OR  TITLE-ABS-KEY ( fishery )  OR  TITLE-ABS-KEY ( fisheries )  OR  TITLE-ABS-KEY ( "fish pond*" )  OR  TITLE-ABS-KEY ( fishpond* )  OR  TITLE-ABS-KEY ( "fish farm*" )  OR  TITLE-ABS-KEY ( fishfarm* ) ) )  OR  ( ( TITLE-ABS-KEY ( livestock* )  OR  TITLE-ABS-KEY ( dair* )  OR  TITLE-ABS-KEY ( poultr* ) ) ) )  AND  ( ( TITLE-ABS-KEY ( nutrition* )  OR  TITLE-ABS-KEY ( haiti  OR  {Sierra Leone}  OR  burundi  OR  korea  OR  ,  2003 )  OR  LIMIT-TO ( PUBYEAR ,  2002 )  OR  LIMIT-TO ( PUBYEAR ,  2001 )  OR  LIMIT-TO ( PUBYEAR ,  2000 ) )  AND  ( LIMIT-TO ( LANGUAGE ,  "English" ) )  AND  ( LIMIT-TO ( DOCTYPE ,  "ar" )  OR  LIMIT-TO ( DOCTYPE ,  "re" ) ) | 5,967 |
| Filter by language (English) | 30 | #29 AND ( (LIMIT-TO ( LANGUAGE ,  "English" ) ) | 7,526 |
| Filter by publication years 2000-2019 | 29 | #28 AND  ( ( LIMIT-TO ( PUBYEAR ,  2019 )  OR  LIMIT-TO ( PUBYEAR ,  2018 )  OR  LIMIT-TO ( PUBYEAR ,  2017 )  OR  LIMIT-TO ( PUBYEAR ,  2016 )  OR  LIMIT-TO ( PUBYEAR ,  2015 )  OR  LIMIT-TO ( PUBYEAR ,  2014 )  OR  LIMIT-TO ( PUBYEAR ,  2013 )  OR  LIMIT-TO ( PUBYEAR ,  2012 )  OR  LIMIT-TO ( PUBYEAR ,  2011 )  OR  LIMIT-TO ( PUBYEAR ,  2010 )  OR  LIMIT-TO ( PUBYEAR ,  2009 )  OR  LIMIT-TO ( PUBYEAR ,  2008 )  OR  LIMIT-TO ( PUBYEAR ,  2007 )  OR  LIMIT-TO ( PUBYEAR ,  2006 )  OR  LIMIT-TO ( PUBYEAR ,  2005 )  OR  LIMIT-TO ( PUBYEAR ,  2004 )  OR  LIMIT-TO ( PUBYEAR ,  2003 )  OR  LIMIT-TO ( PUBYEAR ,  2002 )  OR  LIMIT-TO ( PUBYEAR ,  2001 )  OR  LIMIT-TO ( PUBYEAR ,  2000 ) ) | 7,733 |
| [(Agriculture AND nutrition AND multisector) OR Nutrition sensitive] AND LMICs | 28 | #22 AND #27 | 8,692 |
| Low and Lower Middle-Income countries (LMICs) | 27 | ( TITLE-ABS ( "focus countr*"  OR  "low income"  OR  "lower-middle income"  OR  "developing countr*"  OR  {South Asia}  OR  {South East Asia}  OR  {Sub-saharan Africa}  OR  {Subsaharan Africa}  OR  oceania  OR  pacific  OR  afghanistan  OR  guinea  OR  rwanda  OR  benin  OR  {Guinea-Bissau}  OR  senegal  OR  {Burkina Faso}  OR  haiti  OR  {Sierra Leone}  OR  burundi  OR  korea  OR  somalia  OR  {Central African Republic}  OR  liberia  OR  {South Sudan}  OR  chad  OR  madagascar  OR  tanzania  OR  comoros  OR  malawi  OR  togo  OR  congo  OR  mali  OR  uganda  OR  eritrea  OR  mozambique  OR  zimbabwe  OR  ethiopia  OR  nepal  OR  gambia  OR  niger  OR  angola  OR  indonesia  OR  philippines  OR  armenia  OR  jordan  OR  {Sao Tome}  OR  bangladesh  OR  kenya  OR  {Solomon Islands}  OR  bhutan  OR  kiribati  OR  {Sri Lanka}  OR  bolivia  OR  kosovo  OR  sudan  OR  {Cabo Verde}  OR  kyrgyz*  OR  swaziland  OR  cambodia  OR  lao  OR  laos  OR  syria*  OR  cameroon  OR  lesotho  OR  tajikistan  OR  mauritania  OR  {Timor-Leste}  OR  {Cote d'Ivoire}  OR  {Ivory Coast}  OR  micronesia  OR  tunisia  OR  djibouti  OR  moldova  OR  ukraine  OR  egypt  OR  mongolia  OR  uzbekistan  OR  {El Salvador}  OR  morocco  OR  vanuatu  OR  georgia  OR  myanmar  OR  burma  OR  vietnam  OR  "viet nam"  OR  ghana  OR  nicaragua  OR  {West Bank}  OR  gaza  OR  guatemala  OR  nigeria  OR  yemen  OR  honduras  OR  pakistan  OR  zambia  OR  india  OR  {Papua New Guinea} ) ) | 1,874,639 |
| (Agriculture AND nutrition AND multisector) OR Nutrition sensitive | 22 | #11 AND #18 OR #19 | 35, 465 |
| (Agriculture AND nutrition AND multisector | 21 | #11 AND #18 | 35, 286 |
| Nutrition sensitive | 19 | TITLE-ABS-KEY ( "nutrition-sensitiv*" ) | 258 |
| Multisector total | 18 | #11 AND #12 AND #13 AND #14 AND #15 AND #16 AND #17 | 8,670,369 |
| Social Protection | 17 | ( TITLE-ABS-KEY ( "social inclusion" )  OR  TITLE-ABS-KEY ( "social protection" )  OR  TITLE-ABS-KEY ( "social assistance" )  OR  TITLE-ABS-KEY ( voucher )  OR  TITLE-ABS-KEY ( vouchers )  OR  TITLE-ABS-KEY ( transfer )  OR  TITLE-ABS-KEY ( transfers )  OR  TITLE-ABS-KEY ( "social safety net" )  OR  TITLE-ABS-KEY ( "social safety nets" ) ) | 1,903,197 |
| Natural Resource Management | 16 | TITLE-ABS-KEY ( "natural resource*" ) | 113,440 |
| Education | 15 | ( TITLE-ABS-KEY ( education )  OR  TITLE-ABS-KEY ( school* )  OR  TITLE-ABS-KEY ( "early child development" ) ) | 2,658,113 |
| Health | 14 | ( TITLE-ABS-KEY ( "health service*" )  OR  TITLE-ABS-KEY ( "maternal health" )  OR  TITLE-ABS-KEY ( "child health" )  OR  TITLE-ABS-KEY ( "adolescent health" )  OR  TITLE-ABS-KEY ( "mental health" )  OR  TITLE-ABS-KEY ( "family planning" ) ) | 1,026,381 |
| Water, Sanitation and Hygiene (WASH) | 13 | ( TITLE-ABS-KEY ( sanitation )  OR  TITLE-ABS-KEY ( hygien* )  OR  TITLE-ABS-KEY ( "drinking water" )  OR  TITLE-ABS-KEY ( "safe water" )  OR  TITLE-ABS-KEY ( handwash* )  OR  TITLE-ABS-KEY ( "hand wash*" )  OR  TITLE-ABS-KEY ( "open-defecation" ) ) | 318,363 |
| Multisector general term | 12 | ( TITLE-ABS-KEY ( sector* )  OR  TITLE-ABS-KEY ( multisector* )  OR  TITLE-ABS-KEY ( intersector* )  OR  TITLE-ABS-KEY ( integrat* )  OR  TITLE-ABS-KEY ( convergen* ) ) | 3,368,040 |
| Agriculture AND Nutrition | 11 | #8 AND #9 | 153,641 |
| Nutrition total | 9 | ( TITLE-ABS-KEY ( nutrition* )  OR  TITLE-ABS-KEY ( undernutrition* )  OR  TITLE-ABS-KEY ( malnutrition* )  OR  TITLE-ABS-KEY ( micronutri* )  OR  TITLE-ABS-KEY ( nutrient* )  OR  TITLE-ABS-KEY ( diet )  OR  TITLE-ABS-KEY ( diets )  OR  TITLE-ABS-KEY ( dietar* )  OR  TITLE-ABS-KEY ( food* )  OR  TITLE-ABS-KEY ( fruit* )  OR  TITLE-ABS-KEY ( vegetable* )  OR  TITLE-ABS-KEY ( milk )  OR  TITLE-ABS-KEY ( meat )  OR  TITLE-ABS-KEY ( egg )  OR  TITLE-ABS-KEY ( eggs ) ) | 3,266,488 |
| Agriculture total | 8 | #1 OR #2 OR #3 OR #4 OR #5 OR #6 OR #7 | 533,831 |
| Livestock | 7 | (TITLE-ABS-KEY (livestock* )  OR  TITLE-ABS-KEY ( dair* )  OR  TITLE-ABS-KEY ( poultr* ) ) | 268,297 |
| Aquaculture/fishery | 6 | (TITLE-ABS-KEY ( aquacult* )  OR  TITLE-ABS-KEY ( "aqua cult*" )  OR  TITLE-ABS-KEY ( fishery )  OR  TITLE-ABS-KEY ( fisheries )  OR  TITLE-ABS-KEY ( "fish pond*" )  OR  TITLE-ABS-KEY ( fishpond* )  OR  TITLE-ABS-KEY ( "fish farm*" )  OR  TITLE-ABS-KEY ( fishfarm* ) ) | 150, 804 |
| Water/irrigation | 5 | (TITLE-ABS-KEY (“water management" )  OR  TITLE-ABS-KEY ( irrigation ) ) | 249, 997 |
| Fortification | 4 | (TITLE-ABS-KEY ( fortif* )  OR  TITLE-ABS-KEY ( biofortif* ) ) | 34, 788 |
| Value chain | 3 | (TITLE-ABS-KEY ( "value chain*" )  OR  TITLE-ABS-KEY ( "value crop*" ) ) | 15, 494 |
| Farmer | 2 | (TITLE-ABS-KEY ( farmer )  OR  TITLE-ABS-KEY ( farmers ) ) | 110, 609 |
| Agriculture general | 1 | (TITLE-ABS-KEY ( agricult* )  OR  TITLE-ABS-KEY ( "agri-cult*" ) ) | 604, 992 |

Homestead Food Production (HFP) only, not covered by total search

Date: 16/10/2019, result of difference: 397

| **Theme** | **Query no** | **Query** | **Results** |
| --- | --- | --- | --- |
| Difference in  HFP and total syntax on Scopus (as above) | 3 | # 2 NOT #1 | 397 |
| (Homestead Food Production intervention AND Nutrition AND multisector) OR nutrition sensitive  AND LMIC  Filtered by date, language and article type | 2 | ( ( TITLE-ABS-KEY ( homestead )  OR  TITLE-ABS-KEY ( horticult* )  OR  TITLE-ABS-KEY ( garden* ) ) )  AND  ( ( TITLE-ABS-KEY ( nutrition* )  OR  TITLE-ABS-KEY ( undernutrition* )  OR  TITLE-ABS-KEY ( malnutrition* )  OR  TITLE-ABS-KEY ( micronutri* )  OR  TITLE-ABS-KEY ( nutrient* )  OR  TITLE-ABS-KEY ( diet )  OR  TITLE-ABS-KEY ( diets )  OR  TITLE-ABS-KEY ( dietar* )  OR  TITLE-ABS-KEY ( food* )  OR  TITLE-ABS-KEY ( fruit* )  OR  TITLE-ABS-KEY ( vegetable* )  OR  TITLE-ABS-KEY ( milk )  OR  TITLE-ABS-KEY ( meat )  OR  TITLE-ABS-KEY ( egg )  OR  TITLE-ABS-KEY ( eggs ) ) )  AND  ( ( ( TITLE-ABS-KEY ( sector* )  OR  TITLE-ABS-KEY ( multisector* )  OR  TITLE-ABS-KEY ( intersector* )  OR  TITLE-ABS-KEY ( integrat* )  OR  TITLE-ABS-KEY ( convergen* ) ) )  OR  ( ( TITLE-ABS-KEY ( sanitation )  OR  TITLE-ABS-KEY ( hygien* )  OR  TITLE-ABS-KEY ( "drinking water" )  OR  TITLE-ABS-KEY ( "safe water" )  OR  TITLE-ABS-KEY ( handwash* )  OR  TITLE-ABS-KEY ( "hand wash*" )  OR  TITLE-ABS-KEY ( "open-defecation" ) ) )  OR  ( ( TITLE-ABS-KEY ( "health service*" )  OR  TITLE-ABS-KEY ( "maternal health" )  OR  TITLE-ABS-KEY ( "child health" )  OR  TITLE-ABS-KEY ( "adolescent health" )  OR  TITLE-ABS-KEY ( "mental health" )  OR  TITLE-ABS-KEY ( "family planning" ) ) )  OR  ( ( TITLE-ABS-KEY ( education )  OR  TITLE-ABS-KEY ( school* )  OR  TITLE-ABS-KEY ( "early child development" ) ) )  OR  ( TITLE-ABS-KEY ( "natural resource*" ) )  OR  ( ( TITLE-ABS-KEY ( "social inclusion" )  OR  TITLE-ABS-KEY ( "social protection" )  OR  TITLE-ABS-KEY ( "social assistance" )  OR  TITLE-ABS-KEY ( voucher )  OR  TITLE-ABS-KEY ( vouchers )  OR  TITLE-ABS-KEY ( transfer )  OR  TITLE-ABS-KEY ( transfers )  OR  TITLE-ABS-KEY ( "social safety net" )  OR  TITLE-ABS-KEY ( "social safety nets" ) ) ) )  AND  ( ( TITLE-ABS ( "focus countr*"  OR  "low income"  OR  "lower-middle income"  OR  "developing countr*"  OR  {South Asia}  OR  {South East Asia}  OR  {Sub-saharan Africa}  OR  {Subsaharan Africa}  OR  oceania  OR  pacific  OR  afghanistan  OR  guinea  OR  rwanda  OR  benin  OR  {Guinea-Bissau}  OR  senegal  OR  {Burkina Faso}  OR  haiti  OR  {Sierra Leone}  OR  burundi  OR  korea  OR  somalia  OR  {Central African Republic}  OR  liberia  OR  {South Sudan}  OR  chad  OR  madagascar  OR  tanzania  OR  comoros  OR  malawi  OR  togo  OR  congo  OR  mali  OR  uganda  OR  eritrea  OR  mozambique  OR  zimbabwe  OR  ethiopia  OR  nepal  OR  gambia  OR  niger  OR  angola  OR  indonesia  OR  philippines  OR  armenia  OR  jordan  OR  {Sao Tome}  OR  bangladesh  OR  kenya  OR  {Solomon Islands}  OR  bhutan  OR  kiribati  OR  {Sri Lanka}  OR  bolivia  OR  kosovo  OR  sudan  OR  {Cabo Verde}  OR  kyrgyz*  OR  swaziland  OR  cambodia  OR  lao  OR  laos  OR  syria*  OR  cameroon  OR  lesotho  OR  tajikistan  OR  mauritania  OR  {Timor-Leste}  OR  {Cote d'Ivoire}  OR  {Ivory Coast}  OR  micronesia  OR  tunisia  OR  djibouti  OR  moldova  OR  ukraine  OR  egypt  OR  mongolia  OR  uzbekistan  OR  {El Salvador}  OR  morocco  OR  vanuatu  OR  georgia  OR  myanmar  OR  burma  OR  vietnam  OR  "viet nam"  OR  ghana  OR  nicaragua  OR  {West Bank}  OR  gaza  OR  guatemala  OR  nigeria  OR  yemen  OR  honduras  OR  pakistan  OR  zambia  OR  india  OR  {Papua New Guinea} ) ) )  AND  ( LIMIT-TO ( DOCTYPE ,  "ar" )  OR  LIMIT-TO ( DOCTYPE ,  "ch" )  OR  LIMIT-TO ( DOCTYPE ,  "re" )  OR  LIMIT-TO ( DOCTYPE ,  "ip" ) )  AND  ( LIMIT-TO ( PUBYEAR ,  2019 )  OR  LIMIT-TO ( PUBYEAR ,  2018 )  OR  LIMIT-TO ( PUBYEAR ,  2017 )  OR  LIMIT-TO ( PUBYEAR ,  2016 )  OR  LIMIT-TO ( PUBYEAR ,  2015 )  OR  LIMIT-TO ( PUBYEAR ,  2014 )  OR  LIMIT-TO ( PUBYEAR ,  2013 )  OR  LIMIT-TO ( PUBYEAR ,  2012 )  OR  LIMIT-TO ( PUBYEAR ,  2011 )  OR  LIMIT-TO ( PUBYEAR ,  2010 )  OR  LIMIT-TO ( PUBYEAR ,  2009 )  OR  LIMIT-TO ( PUBYEAR ,  2008 )  OR  LIMIT-TO ( PUBYEAR ,  2007 )  OR  LIMIT-TO ( PUBYEAR ,  2006 )  OR  LIMIT-TO ( PUBYEAR ,  2005 )  OR  LIMIT-TO ( PUBYEAR ,  2004 )  OR  LIMIT-TO ( PUBYEAR ,  2003 )  OR  LIMIT-TO ( PUBYEAR ,  2002 )  OR  LIMIT-TO ( PUBYEAR ,  2001 ) )  AND  ( LIMIT-TO ( LANGUAGE ,  "English" ) ) | 729 |
| Total syntax on Scopus carried out on 25^th^ March, 2019, refreshed on October 16, 2019 | 1 | ( ( TITLE-ABS-KEY ( "nutrition-sensitiv*" ) )  OR  ( ( ( ( ( TITLE-ABS-KEY ( agricult* )  OR  TITLE-ABS-KEY ( "agri-cult*" ) ) )  OR  #  2  OR  ( ( TITLE-ABS-KEY ( "value chain*" )  OR  TITLE-ABS-KEY ( "value crop*" ) ) )  OR  ( ( TITLE-ABS-KEY ( fortif* )  OR  TITLE-ABS-KEY ( biofortif* ) ) )  OR  ( ( TITLE-ABS-KEY ( "water management" )  OR  TITLE-ABS-KEY ( irrigation ) ) )  OR  ( ( TITLE-ABS-KEY ( aquacult* )  OR  TITLE-ABS-KEY ( "aqua cult*" )  OR  TITLE-ABS-KEY ( fishery )  OR  TITLE-ABS-KEY ( fisheries )  OR  TITLE-ABS-KEY ( "fish pond*" )  OR  TITLE-ABS-KEY ( fishpond* )  OR  TITLE-ABS-KEY ( "fish farm*" )  OR  TITLE-ABS-KEY ( fishfarm* ) ) )  OR  ( ( TITLE-ABS-KEY ( livestock* )  OR  TITLE-ABS-KEY ( dair* )  OR  TITLE-ABS-KEY ( poultr* ) ) ) )  AND  ( ( TITLE-ABS-KEY ( nutrition* )  OR  TITLE-ABS-KEY ( undernutrition* )  OR  TITLE-ABS-KEY ( malnutrition* )  OR  TITLE-ABS-KEY ( micronutri* )  OR  TITLE-ABS-KEY ( nutrient* )  OR  TITLE-ABS-KEY ( diet )  OR  TITLE-ABS-KEY ( diets )  OR  TITLE-ABS-KEY ( dietar* )  OR  TITLE-ABS-KEY ( food* )  OR  TITLE-ABS-KEY ( fruit* )  OR  TITLE-ABS-KEY ( vegetable* )  OR  TITLE-ABS-KEY ( milk )  OR  TITLE-ABS-KEY ( meat )  OR  TITLE-ABS-KEY ( egg )  OR  TITLE-ABS-KEY ( eggs ) ) ) )  AND  ( ( ( TITLE-ABS-KEY ( sector* )  OR  TITLE-ABS-KEY ( multisector* )  OR  TITLE-ABS-KEY ( intersector* )  OR  TITLE-ABS-KEY ( integrat* )  OR  TITLE-ABS-KEY ( convergen* ) ) )  OR  ( ( TITLE-ABS-KEY ( sanitation )  OR  TITLE-ABS-KEY ( hygien* )  OR  TITLE-ABS-KEY ( "drinking water" )  OR  TITLE-ABS-KEY ( "safe water" )  OR  TITLE-ABS-KEY ( handwash* )  OR  TITLE-ABS-KEY ( "hand wash*" )  OR  TITLE-ABS-KEY ( "open-defecation" ) ) )  OR  ( ( TITLE-ABS-KEY ( "health service*" )  OR  TITLE-ABS-KEY ( "maternal health" )  OR  TITLE-ABS-KEY ( "child health" )  OR  TITLE-ABS-KEY ( "adolescent health" )  OR  TITLE-ABS-KEY ( "mental health" )  OR  TITLE-ABS-KEY ( "family planning" ) ) )  OR  ( ( TITLE-ABS-KEY ( education )  OR  TITLE-ABS-KEY ( school* )  OR  TITLE-ABS-KEY ( "early child development" ) ) )  OR  ( TITLE-ABS-KEY ( "natural resource*" ) )  OR  ( ( TITLE-ABS-KEY ( "social inclusion" )  OR  TITLE-ABS-KEY ( "social protection" )  OR  TITLE-ABS-KEY ( "social assistance" )  OR  TITLE-ABS-KEY ( voucher )  OR  TITLE-ABS-KEY ( vouchers )  OR  TITLE-ABS-KEY ( transfer )  OR  TITLE-ABS-KEY ( transfers )  OR  TITLE-ABS-KEY ( "social safety net" )  OR  TITLE-ABS-KEY ( "social safety nets" ) ) ) ) ) )  AND  ( ( TITLE-ABS ( "focus countr*"  OR  "low income"  OR  "lower-middle income"  OR  "developing countr*"  OR  {South Asia}  OR  {South East Asia}  OR  {Sub-saharan Africa}  OR  {Subsaharan Africa}  OR  oceania  OR  pacific  OR  afghanistan  OR  guinea  OR  rwanda  OR  benin  OR  {Guinea-Bissau}  OR  senegal  OR  {Burkina Faso}  OR  haiti  OR  {Sierra Leone}  OR  burundi  OR  korea  OR  somalia  OR  {Central African Republic}  OR  liberia  OR  {South Sudan}  OR  chad  OR  madagascar  OR  tanzania  OR  comoros  OR  malawi  OR  togo  OR  congo  OR  mali  OR  uganda  OR  eritrea  OR  mozambique  OR  zimbabwe  OR  ethiopia  OR  nepal  OR  gambia  OR  niger  OR  angola  OR  indonesia  OR  philippines  OR  armenia  OR  jordan  OR  {Sao Tome}  OR  bangladesh  OR  kenya  OR  {Solomon Islands}  OR  bhutan  OR  kiribati  OR  {Sri Lanka}  OR  bolivia  OR  kosovo  OR  sudan  OR  {Cabo Verde}  OR  kyrgyz*  OR  swaziland  OR  cambodia  OR  lao  OR  laos  OR  syria*  OR  cameroon  OR  lesotho  OR  tajikistan  OR  mauritania  OR  {Timor-Leste}  OR  {Cote d'Ivoire}  OR  {Ivory Coast}  OR  micronesia  OR  tunisia  OR  djibouti  OR  moldova  OR  ukraine  OR  egypt  OR  mongolia  OR  uzbekistan  OR  {El Salvador}  OR  morocco  OR  vanuatu  OR  georgia  OR  myanmar  OR  burma  OR  vietnam  OR  "viet nam"  OR  ghana  OR  nicaragua  OR  {West Bank}  OR  gaza  OR  guatemala  OR  nigeria  OR  yemen  OR  honduras  OR  pakistan  OR  zambia  OR  india  OR  {Papua New Guinea} ) ) )  AND  ( LIMIT-TO ( PUBYEAR ,  2019 )  OR  LIMIT-TO ( PUBYEAR ,  2018 )  OR  LIMIT-TO ( PUBYEAR ,  2017 )  OR  LIMIT-TO ( PUBYEAR ,  2016 )  OR  LIMIT-TO ( PUBYEAR ,  2015 )  OR  LIMIT-TO ( PUBYEAR ,  2014 )  OR  LIMIT-TO ( PUBYEAR ,  2013 )  OR  LIMIT-TO ( PUBYEAR ,  2012 )  OR  LIMIT-TO ( PUBYEAR ,  2011 )  OR  LIMIT-TO ( PUBYEAR ,  2010 )  OR  LIMIT-TO ( PUBYEAR ,  2009 )  OR  LIMIT-TO ( PUBYEAR ,  2008 )  OR  LIMIT-TO ( PUBYEAR ,  2007 )  OR  LIMIT-TO ( PUBYEAR ,  2006 )  OR  LIMIT-TO ( PUBYEAR ,  2005 )  OR  LIMIT-TO ( PUBYEAR ,  2004 )  OR  LIMIT-TO ( PUBYEAR ,  2003 )  OR  LIMIT-TO ( PUBYEAR ,  2002 )  OR  LIMIT-TO ( PUBYEAR ,  2001 )  OR  LIMIT-TO ( PUBYEAR ,  2000 ) )  AND  ( LIMIT-TO ( LANGUAGE ,  "English" ) )  AND  ( LIMIT-TO ( DOCTYPE ,  "ar" )  OR  LIMIT-TO ( DOCTYPE ,  "ch" )  OR  LIMIT-TO ( DOCTYPE ,  "re" )  OR  LIMIT-TO ( DOCTYPE ,  "ip" ) ) | 7,193 |

Database 4 Full electronic search syntax applied in web of science on 25/03/2019, with 8,399 results.

| **Theme** | **Query no.** | **Query** | **Results** |
| --- | --- | --- | --- |
| Filter by document type (article or book chapter or review) | # 28 | #27  Refined by: DOCUMENT TYPES: (ARTICLE OR BOOK CHAPTER OR REVIEW) | [8,399](http://apps.webofknowledge.com.vu-nl.idm.oclc.org/summary.do?product=WOS&doc=1&qid=30&SID=D4XX2rrmSYCsV3JMghl&search_mode=AdvancedSearch&update_back2search_link_param=yes) |
| Filter by language (English) | # 27 | #26  Refined by: LANGUAGES: (ENGLISH) | [8,491](http://apps.webofknowledge.com.vu-nl.idm.oclc.org/summary.do?product=WOS&doc=1&qid=29&SID=D4XX2rrmSYCsV3JMghl&search_mode=AdvancedSearch&update_back2search_link_param=yes) |
| Filter by publication years | # 26 | #25  Refined by: PUBLICATION YEARS: (2019 OR 2010 OR 2001 OR 2018 OR 2009 OR 2000 OR 2017 OR 2008 OR 2016 OR 2007 OR 2015 OR 2006 OR 2014 OR 2005 OR 2013 OR 2004 OR 2012 OR 2003 OR 2011 OR 2002) | [8,659](http://apps.webofknowledge.com.vu-nl.idm.oclc.org/summary.do?product=WOS&doc=1&qid=28&SID=D4XX2rrmSYCsV3JMghl&search_mode=AdvancedSearch&update_back2search_link_param=yes) |
| [(Agriculture AND Nutrition AND Multisector) OR Nutrition Sensitive] AND LMICs | # 25 | #23 AND #24 | [9,164](http://apps.webofknowledge.com.vu-nl.idm.oclc.org/summary.do?product=WOS&doc=1&qid=27&SID=D4XX2rrmSYCsV3JMghl&search_mode=AdvancedSearch&update_back2search_link_param=yes) |
| Low and lower-middle income countries (LMICs) | # 24 | TS=("low income" OR "lower-middle income" OR "developing countr*" OR "focus countr*" OR "Sub-saharan Africa" OR "Subsaharan Africa" OR "South Asia" OR "South East Asia" OR Oceania OR Pacific OR Afghanistan OR Angola OR Armenia OR Bangladesh OR Benin OR Bolivia OR "Burkina Faso" OR Burma OR Burundi OR Bhutan OR "Cabo Verde" OR Cambodia OR Cameroon OR "Central African Republic" OR Chad OR Comoros OR Congo OR "Côte d’Ivoire" OR Djibouti OR Egypt OR "El Salvador" OR Eritrea OR Ethiopia OR Gambia OR Gaza OR Georgia OR Ghana OR Guatemala OR Guinea OR "Guinea-Bissau" OR Haiti OR Honduras OR India OR Indonesia OR "Ivory Coast" OR Jordan OR Kenya OR Kiribati OR Korea OR Kosovo OR Kyrgyz* OR Lao OR Laos OR Lesotho OR Liberia OR Madagascar OR Malawi OR Mali OR Mauritania OR Micronesia OR Moldova OR Mongolia OR Morocco OR Mozambique OR Myanmar OR Nepal OR Nicaragua OR Niger OR Nigeria OR Pakistan OR "Papua New Guinea" OR Philippines OR Rwanda OR "Sao Tome" OR Senegal OR "Sierra Leone" OR "Solomon Islands" OR Somalia OR Sudan OR "South Sudan" OR "Sri Lanka" OR Swaziland OR Syria* OR Tajikistan OR Tanzania OR "Timor-Leste" OR Togo OR Tunisia OR Vanuatu OR Vietnam OR "Viet Nam" OR Uganda OR Ukraine OR Uzbekistan OR "West Bank" OR Yemen OR Zambia OR Zimbabwe) | [1,473,320](http://apps.webofknowledge.com.vu-nl.idm.oclc.org/summary.do?product=WOS&doc=1&qid=26&SID=D4XX2rrmSYCsV3JMghl&search_mode=AdvancedSearch&update_back2search_link_param=yes) |
| (Agriculture AND Nutrition AND Multisector) OR Nutrition Sensitive | # 23 | #21 OR #22 | [40,653](http://apps.webofknowledge.com.vu-nl.idm.oclc.org/summary.do?product=WOS&doc=1&qid=25&SID=D4XX2rrmSYCsV3JMghl&search_mode=AdvancedSearch&update_back2search_link_param=yes) |
| Agriculture AND Nutrition AND Multisector | # 22 | #13 AND #20 | [40,487](http://apps.webofknowledge.com.vu-nl.idm.oclc.org/summary.do?product=WOS&doc=1&qid=24&SID=D4XX2rrmSYCsV3JMghl&search_mode=AdvancedSearch&update_back2search_link_param=yes) |
| Nutrition sensitive | # 21 | TS=("nutrition-sensitiv*") | [254](http://apps.webofknowledge.com.vu-nl.idm.oclc.org/summary.do?product=WOS&doc=1&qid=23&SID=D4XX2rrmSYCsV3JMghl&search_mode=AdvancedSearch&update_back2search_link_param=yes) |
| Multisector all | # 20 | #14 OR #15 OR #16 OR #17 OR #18 OR #19 | [4,319,293](http://apps.webofknowledge.com.vu-nl.idm.oclc.org/summary.do?product=WOS&doc=1&qid=22&SID=D4XX2rrmSYCsV3JMghl&search_mode=AdvancedSearch&update_back2search_link_param=yes) |
| Natural Resource Management | # 19 | TS=("natural resource*") | [31,874](http://apps.webofknowledge.com.vu-nl.idm.oclc.org/summary.do?product=WOS&doc=1&qid=21&SID=D4XX2rrmSYCsV3JMghl&search_mode=AdvancedSearch&update_back2search_link_param=yes) |
| Social Protection | # 18 | TS=("social inclusion" OR "social protection" OR "social assistance" OR voucher OR vouchers OR transfer OR transfers OR "social safety net*") | [1,352,161](http://apps.webofknowledge.com.vu-nl.idm.oclc.org/summary.do?product=WOS&doc=1&qid=20&SID=D4XX2rrmSYCsV3JMghl&search_mode=AdvancedSearch&update_back2search_link_param=yes) |
| Education | # 17 | TS=(education OR school* OR "early child development") | [1,087,211](http://apps.webofknowledge.com.vu-nl.idm.oclc.org/summary.do?product=WOS&doc=1&qid=19&SID=D4XX2rrmSYCsV3JMghl&search_mode=AdvancedSearch&update_back2search_link_param=yes) |
| Health | # 16 | TS=("health service*" OR "maternal health" OR "child health" OR "adolescent health" OR "mental health" OR "family planning") | [285,703](http://apps.webofknowledge.com.vu-nl.idm.oclc.org/summary.do?product=WOS&doc=1&qid=18&SID=D4XX2rrmSYCsV3JMghl&search_mode=AdvancedSearch&update_back2search_link_param=yes) |
| Water, Sanitation and Hygiene (WASH) | # 15 | TS=(sanitation OR hygien* OR "drinking water" OR "safe water" OR handwash* OR "hand wash*" OR "open-defecation") | [142,714](http://apps.webofknowledge.com.vu-nl.idm.oclc.org/summary.do?product=WOS&doc=1&qid=17&SID=D4XX2rrmSYCsV3JMghl&search_mode=AdvancedSearch&update_back2search_link_param=yes) |
| Multisector general | # 14 | TS=(sector* OR multisector* OR intersector* OR integrat* OR convergen*) | [1,672,277](http://apps.webofknowledge.com.vu-nl.idm.oclc.org/summary.do?product=WOS&doc=1&qid=16&SID=D4XX2rrmSYCsV3JMghl&search_mode=AdvancedSearch&update_back2search_link_param=yes) |
| Agriculture AND Nutrition | # 13 | #9 AND #12 | [275,995](http://apps.webofknowledge.com.vu-nl.idm.oclc.org/summary.do?product=WOS&doc=1&qid=15&SID=D4XX2rrmSYCsV3JMghl&search_mode=AdvancedSearch&update_back2search_link_param=yes) |
| Nutrition total | # 12 | #10 OR #11 | [2,265,243](http://apps.webofknowledge.com.vu-nl.idm.oclc.org/summary.do?product=WOS&doc=1&qid=14&SID=D4XX2rrmSYCsV3JMghl&search_mode=AdvancedSearch&update_back2search_link_param=yes) |
| Nutrition term | # 11 | TS=(nutrition* OR undernutrition* OR malnutrition* OR micronutri*) | [398,272](http://apps.webofknowledge.com.vu-nl.idm.oclc.org/summary.do?product=WOS&doc=1&qid=13&SID=D4XX2rrmSYCsV3JMghl&search_mode=AdvancedSearch&update_back2search_link_param=yes) |
| Food or diet | # 10 | TS=(food* OR fruit* OR vegetable* OR milk OR meat OR egg OR eggs OR diet OR diets OR dietar* OR nutrient*) | [2,050,474](http://apps.webofknowledge.com.vu-nl.idm.oclc.org/summary.do?product=WOS&doc=1&qid=12&SID=D4XX2rrmSYCsV3JMghl&search_mode=AdvancedSearch&update_back2search_link_param=yes) |
| Agriculture total | # 9 | #1 OR #2 OR #3 OR #4 OR #5 OR #6 OR #7 OR #8 | [946,483](http://apps.webofknowledge.com.vu-nl.idm.oclc.org/summary.do?product=WOS&doc=1&qid=11&SID=D4XX2rrmSYCsV3JMghl&search_mode=AdvancedSearch&update_back2search_link_param=yes) |
| Farmer | # 8 | TS=(farmer OR farmers) | [72,302](http://apps.webofknowledge.com.vu-nl.idm.oclc.org/summary.do?product=WOS&doc=1&qid=10&SID=D4XX2rrmSYCsV3JMghl&search_mode=AdvancedSearch&update_back2search_link_param=yes) |
| Aquaculture/fishery | # 7 | TS=(aquacult* OR "aqua cult*" OR fishery OR fisheries OR "fish pond*" OR fishpond* OR fishfarm* OR "fish farm*") | [89,871](http://apps.webofknowledge.com.vu-nl.idm.oclc.org/summary.do?product=WOS&doc=1&qid=9&SID=D4XX2rrmSYCsV3JMghl&search_mode=AdvancedSearch&update_back2search_link_param=yes) |
| Water/irrigation | # 6 | TS=("water management" OR irrigation) | [94,597](http://apps.webofknowledge.com.vu-nl.idm.oclc.org/summary.do?product=WOS&doc=1&qid=8&SID=D4XX2rrmSYCsV3JMghl&search_mode=AdvancedSearch&update_back2search_link_param=yes) |
| Livestock | # 5 | TS=(livestock* OR dair* OR poultr*) | [209,101](http://apps.webofknowledge.com.vu-nl.idm.oclc.org/summary.do?product=WOS&doc=1&qid=7&SID=D4XX2rrmSYCsV3JMghl&search_mode=AdvancedSearch&update_back2search_link_param=yes) |
| Homestead production | # 4 | TS=(homestead OR horticult* OR garden* OR harvest*) | [268,206](http://apps.webofknowledge.com.vu-nl.idm.oclc.org/summary.do?product=WOS&doc=1&qid=6&SID=D4XX2rrmSYCsV3JMghl&search_mode=AdvancedSearch&update_back2search_link_param=yes) |
| Fortification | # 3 | TS=(fortif* OR biofortif*) | [23,920](http://apps.webofknowledge.com.vu-nl.idm.oclc.org/summary.do?product=WOS&doc=1&qid=5&SID=D4XX2rrmSYCsV3JMghl&search_mode=AdvancedSearch&update_back2search_link_param=yes) |
| Value chain | # 2 | TS=("value chain*" OR "value crop*") | [7,255](http://apps.webofknowledge.com.vu-nl.idm.oclc.org/summary.do?product=WOS&doc=1&qid=4&SID=D4XX2rrmSYCsV3JMghl&search_mode=AdvancedSearch&update_back2search_link_param=yes) |
| Agriculture general | # 1 | TS=(agricult* OR "agri-cult*") | [298,260](http://apps.webofknowledge.com.vu-nl.idm.oclc.org/summary.do?product=WOS&doc=1&qid=3&SID=D4XX2rrmSYCsV3JMghl&search_mode=AdvancedSearch&update_back2search_link_param=yes) |
