# Supplementary material for: Nutrition-Sensitive Agriculture: A Systematic Review of Impact Pathways to Nutrition Outcomes
Source: Adv Nutr. 2020 Sep 24;12(1):251–75. doi: 10.1093/advances/nmaa103 (PMC7850060; doi:10.1093/advances/nmaa103)
Supplement: nmaa103_Supplemental_Files [file nmaa103_supplemental_files.zip › Supplemental_table_2_Detailed_characteristics_and_findings_Aug_31.docx]

***Supplemental Table 2.* Detailed characteristics and findings of individual studies, NSA impact pathways to nutrition outcomes (n=43)^[[1]](#footnote-1)^**

| **Source, Country** | **Study design** | **Agriculture interventions** | **Nutrition specific and nutrition sensitive interventions** | **Data collection methods,**  **study population & size** | **Key outcome variable** | **Results on pathways and impact** | **Risk of bias** |
| --- | --- | --- | --- | --- | --- | --- | --- |
| **HFP: Poultry & Nutrition Education/specific components** | | | | | |  | |
| Berti & Cossio,  2017 (59)  Bolivia | Non-randomized intervention | Inputs: Corrugated zinc (shelter); Vaccination against Newcastle disease, as a starter flock; 8 Pirocas and 2 locally adapted breed (sometimes 3-4 male)  Training: Chicken rearing workshops  Training: chicken feeding, breeding, & disease, & pest management | Nutrition education Nutrition workshops Topics: importance of a good diet, egg consumption, breastfeeding (early initiation, exclusive breastfeeding, and continued breastfeeding) | - Surveys at baseline (2013), follow-up (2014) & end line (2015) - Children < 6 y, adult men & women at HHs (baseline: 22 follow-ups:33 endline:66) | Consumption of food groups in children, men and women | **Pathway** ↑ Egg production (established in 80% of intervention HH^[[2]](#footnote-2)^s)  **Impact**  *men, women and children*  ↑ Egg consumption (↑ by 0.5 egg /p/day, p<0.0001), no change on dairy, fruits, legumes, meat, sweets ↑ Iron intake mg/day (children: p=0.03, women: p=0.02, men: p=0.00), no impact on mg/1000 kcal No impact on intake of energy, protein, calcium, zinc, thiamin, riboflavin or niacin | High |
| **HFP: Vegetables and /or fruits; & Nutrition Education/specific components** | | | | | |  | |
| Birdi & Shah,  2015  (60)  India | Longitudinal study, followed up for 2 years | Inputs  Kitchen Garden Plant Distribution: one tree (perennial) and one GLV package | Nutrition education Community interactions, IEC and home visits on nutrition (importance of nutrition and role of KG, importance of incorporating the intervention plants in diet, childcare practices: complementary feeding) | - Surveys at baseline (winter 2011), 3 follow ups (summer 2012, monsoon 2012, winter 2012) & endline (summer 2013) - Adult member of HH with at least one child aged <6 (baseline: 396; 1^st^ follow up: 203, 2^nd^ follow up: 198; 3^rd^ follow up: 259; endline: 362) | Micronutrient deficiency (signs and symptoms), kitchen garden practices, dietary intake patterns & food habits of HHs | **Pathways**  *Production* ↑kitchen garden practice in all season (endline:100% from 79%) ↑KG maintenance in summer (95% from 4%)  **Impact**  *Household* ↑ GLV consumption in winter (100% in 2012 from 80% in 2011) & summer (90% in 2013 from 49% in 2012) ↑ Variety of GLV consumption > 3 varieties (49% from 22%) No change in quantity of GLV consumption in year, ↑ in winter only  ↑ Egg consumption (winter: 3-16%; summer:8-30%)  No change in variety of cereals & pulses consumption  *Children*: No impact: including intervention plants in weaning | High |
| Bushamuka et al.  2005 (40)  Bangladesh | One time cross-sectional  (ben vs control) | Homestead gardening | Nutrition education sessions Gender interventions | - Survey (Feb-March, 2002) (project launched in 1993) - 2,160 HHs: 711 active beneficiaries., 683 former beneficiary& 603 controls | Homestead gardening practices, production & consumption  income & expenditure | **Pathways**  *Production* ↑production of veg (P<0.05) & fruits (P<0.05) ↑ diversification of vegetable & fruits (P<0.05) *Income*  ↑Income (active: 490; former:347; & control: 200 taka) ↑spending on food, health, education, clothing, productive assets, housing & social activities (P≤0.05) *Women empowerment*  **↑**Participation in group meetings, determining women's daily workload, visiting stores/large markets (p≤0.05) & economic contribution to HH  ↑ Decision making: use HH land, type & quantity of veg & fruits consumed in HH, & making HH purchases (p≤0.05 for each attributes)  **Impact** (*household)*  ↑Consumption of veg (P<0.05) and fruits (P<0.05) | High |
| Diana et al.**,** (47)  2014  Indonesia | Quasi experimental study  2 groups | - Inputs: a package of home garden vegetable plants - Socialization, observation, determination of the type of plants and implementation | Nutrition extension program | - Baseline and endline surveys,  Study duration: December 2011 until June 2013 - Mothers (n=61), intervention: 30, & control: 31 | Vegetable consumption, Vitamin A intake | **Pathways**  *Production*: no impact on size of home garden  ↑ *Knowledge*: Nutrition knowledge increased by 12.3 point for home garden plus nutrition extension, versus, home garden only (P<0.001) Reduced food expenditure due to increased production (P<0.01)  **Impact** (*household*) No impact on vegetable consumption, or, vitamin A intake | High |
| Doocy et al.  2019  (61)  Democratic Republic of Congo | Quasi experimental study  (4 groups) | Intervention: HG inputs & nutrition extension  2) Control: no interventions  FFS group   - Training on production, farming education, post-harvest, and business skills - Inputs: seeds & tools - Transition of FFS into business associations   F2F group   - Less resource-intensive FFS participants trained farmers   PM2A group   - HG promotion, monthly rations (corn–soya blend and vitamin A-fortified oil) - Training on child health & nutrition, group meetings and during home visits;   WEG group   - Weekly group meeting - Literacy and numeracy mechanism, business and marketing training, and income-generating activities; Starter kit for income-generating, goats & energy-efficient stoves | - Nutrition in PM2A   -Supplementation: monthly rations (corn–soya blend and vitamin A-fortified oil) -Nutrition education: training on child health & nutrition, care group meetings and during home visits  Women empowerment: WEG Meeting, training and inputs for women empowerment and income generation | - Surveys at baseline (Aug & Oct 2012) & eight semi-annual surveys (Aug/Sept & Feb/Mar) - 1312 children from 1113 HHs | Children's dietary diversity, meal frequency, MAD, HAZ & WAZ scores | Pathways not reported  **Impact** (*children*)  ↑ Mean DDS: PM2A (β=0·55; 95% CI 0·20, 0·91; P=0·002); FFS (β=0·46; 95% CI −0·01, 0·93; P=0·049), no impact on other groups  ↑ Minimum DDS: PM2A (16·9%; 95% CI 4·0, 29·7%; P=0·009); FFS(14·4%; 95% CI 0·2, 28·5%; P =0·042), no impact on other groups ↑ Mean Meal Frequency: WEG(0·25; 95% CI 0·02, 0·47; P=0·027); FFS (0·30; CI 0·05, 0·55; P=0·015); & PM2A(0·32; 95% CI 0·15, 0·49; P<0·001) ↑ Children achieving MMF (P<0·001) or MAD (P=0·009) in PM2A) No impact on stunting, or underweight | Medium |
| Doocy  et al.  2018 (62)  Democratic Republic of Congo |  |  |  | - Surveys at baseline (2011) & endline (2016) surveys - 1625 beneficiary HHs | HDDS  HFIAS | Pathways not reported  **Impact** (*household*) ↑Mean HDDS: WEG (ß: 0.69, p < 0.001), PM2A (ß: 0.75, CI: 0.32–1.18, p = 0.001), & FFS (ß: 0.88, CI: 0.46–1.3, p < 0.001), not on F2F  ↑ Food security (based on HFIAS):  PM2A (Diff: 26.7%, CI: 16.2– 37.3%, p < 0.001), WEG (Diff: 25.8%, CI: 14.6–36.9%, p < 0.001) & FFS (Diff: 22.9%, CI: 12.7–33.1%, p < 0.001); F2F (Diff: 15.0%, CI: 3.8–26.3%, p = 0.002) | Medium |
| **HFP: Vegetables and / or fruits; Poultry; & Nutrition Education/specific components** | | | | | |  | |
| Boedecker et al.  2018 (63)  Kenya | Pair matched study | Training: kitchen gardening, and poultry keeping, from the local Ministry of Agriculture, Livestock, and Fisheries | Nutrition education   - Training (local health ministry) - theoretical classes, cooking sessions, and individual HH or “door‐to‐door” nutrition education. - Topics: diverse diet, CF, nutrition during pregnancy and lactation. | - Baseline (Nov 2015) & endline (Nov 2016) surveys - Women (15-49 y) & children (12-23 mo) pairs - Baseline: 330 HHs (165 intervention, 165 control) & endline: 498 HHs (168 intervention, 165 indirect bens. & 165 control) | Mean DDS, Percentage reaching MDD & micronutrient adequacy (MAR) | Pathways not reported  **Impact**  *Children* ↑ Consumption of legumes & nuts (TE = 0.2, p = 0.002), dairy (TE = 0.2, p = 0.001) & flesh foods (TE = 0.2, p = 0.016), no impact on eggs, fruits& veg ↑ Mean DDS (TE = 0.7, p < 0.001) & share of children reaching MDD (TE = 0.2, p < 0.001), no impact on MAR *Women* No impact on food group consumption except for dairy  No impact on DDS, MDD, MAR | High |
| Dulal  et al,  2017 (64)  Nepal | Repeated cross-sectional design | - Training/technical support: optimal techniques on vegetable gardening & poultry production; creation and facilitation of EHFP beneficiary) groups. - Inputs: 5 chicks per household, seasonal distribution of vegetable seeds | - Nutrition and health BCC   Promotion of ENAs and EHAs | - 3 monitoring surveys: June/July 2014; Nov/Dec, 2014 & April/May 2015 - Mothers of children aged 6–23 mo (survey 1: 821; survey 2: 1094, & survey 3: 1014) | Maternal and child dietary diversity | Pathways not reported  **Impact**  *Children:* Participation in EHFP associated with ↑ CDD in Terai region during winter (B-coeff=0.35, P < .001), no impact for mountain region or rainy season *Mothers:* Participation in EHFP associated with ↑maternal dietary diversity in winter in mountains (B-coef= 0.12, P < .01) & Terai (B-coef=0.24, P < .001), no impact in rainy | High |
| Heckert, Olney & Ruel  2019  (54)  Burkina Faso | RCT  3 groups  Data from two treatment arms (BCC provided by OWL and BCC provided by HC) pooled for analysis | - Training and inputs (e.g., tools, seeds, chickens) - Promote small-scale agriculture and production of nutrient-rich foods (e.g., eggs, vegetables) for sale and consumption | BCC  Promote optimal health and nutrition practices, delivered by either OWL or HC members Women empowerment Community-focused activities to develop land-use agreements | - Household baseline (Feb/May 2010) & endline (Feb-/June 2012) surveys - Mothers of children 3-12 mo (for analysis purpose) - 1265 to 1058 for the cross-sectional analysis & 1035 for mediation analysis | Wasting & Haemoglobin  Measures of women empowerment | **Pathways**  *Women empowerment* Mediation:  ↑ WE score (1.9 of the overall 7.5 pp ↓ in wasting)  ↑Spousal communication affects ↓wasting (β=−0.010; CI=-0.022, -0.001). ↑Purchasing decision & ↓in wasting (β=-0.003) ↑Health care decision & ↓in wasting (β=-0.003) ↑ Family planning decision & ↓ wasting (β=-0.003)  Increase purchasing  decisions (β=0.093; CI=(0.044, 0.145) and spousal communication(β=0.149; CI=(0.084, 0.214), no effect on healthcare (β=0.042; CI=(-0.016, 0.100)) or family planning decisions (β=0.047; CI=(-0.009, 0.113)).    **Impact***: Children*  No effect on hb (0.104)  Marginal effect on wasting (0.060) | Medium |
| Jones  et al,  2005  (48)  Nepal | One-time cross-sectional survey comparing interventions vs control | - Nutrient dense vegetables & fruits identification - Seeds/sapling distribution of veg. plants and mango - Establish model kitchen garden - Kitchen-garden training, recipe demonstration - Household kitchen gardens visit: provide seeds & instructions - Training & mobilizing local “agrovet” businesses to enhance supply | Nutrition and WASH education   - Material on production & nutrition facts of a specific crop - Education on VA and iron-deficiency, improved hygiene & sanitation practices, & modern food-preservation techniques. - Training: nutrition during pregnancy; BF; CF practices; VA; and iron (incl. anaemia) - HH visit & nutrition education to the communities. | - Cross-sectional endline survey after 36 mo of implementation, comparing project HHs & control HHs - Self-identified primary caregivers of children (ben HHs: 430; non- ben HHs: 389) | Nutrition knowledge Production & consumption  Food preservation & storage  Maternal nutrition practices  Hygiene & sanitation | **Pathways**  ↑*Production* for consumption (papaya: P < .05; other crops: p < .001) Expenditure: ↑ Buying vegetables for consumption by controls *Knowledge* ↑Nutrition knowledge (P<0.0001) (cause of night blindness, sources of VA & iron rich foods, anaemia, IYCF)  **Impact**  *Household*  ↑Food preservation last year (86.9% ben; 60.1% non-ben, p < .005); & preserving enough for lean period (49.1% ben; 38.3%non-ben, p <.05). ↑Consumption variety (13veg) & frequency of veg; and mango (P<0.001)  No difference on consumption of ASF *Child care & WASH practices* No impact on feeding colostrum, feeding milk other than BF or length of EBF, CF↑ (P<0.05)  ↑Hand-washing caregivers & children (P<0.05) ↑Keeping flies away when cooking & from children’s food (P<0.005) No impact on access to sanitation facility (latrine) | High |
| Marquis  et al. 2018  (65)  Ghana | Cluster Randomized Control Trial (intervention & control) | Poultry for egg production   - Training on coop construction, feeding and caring of poultry, use of poultry manure, and handling, and marketing of eggs - Weekly technical assistance on poultry production & poultry health management - Inputs: chicken, initial feed for 1 month, & no-cost vaccinations - Facilitate egg sales for women who could not access markets   Home gardens   - Training on HG ( site selection, fencing, seedbed preparation, compost preparation and use, organic weed, insect, and pest control. - Weekly technical assistance - Encourage container gardening - Inputs: seeds & vines of nutrient‐rich vegetables such as kontomire, tomatoes, and OFSP | Nutrition and gender education  *Group education*   - Young child diet and health, with special emphasis on diet diversity and consumption of eggs, GLVs, and OFSP. - Eight lessons on psychosocial stimulation of young children   *Community‐wide education*   - Training on food demonstration sessions, mother‐to‐mother support to encouraged optimal child‐feeding practices, enhanced community‐based growth monitoring and promotion - Community‐wide discussions on gender and diversity | - Surveys at phase 1 (2014-2015) & phase 2 (2016-2017) - Phase 1: 135 HHs (women with <12 mo children); phase 2: 114 HHs (women with children <18 mo) | MDD;  consumption of eggs; and stunting, wasting & underweight | Pathways not reported  Impact (children) ↑MDD (aOR = 1.65, 95% CI [1.02, 2.69]) No change in consumption of egg (aOR = 1.35, 95% CI [0.83, 2.20]) ↑ LAZ/HAZ score: adjusted: (β = 0.22, 95% CI [0.09, 0.34]); random‐effects model: β = 0.21, 95% CI [0.09, 0.34]; mixed‐effects model β = 0.22, 95% CI [0.07, 0.36]; as‐treated model β=0.25, 95% CI [0.10, 0.41]).  ↑ WAZ score adjusted model: (β = 0.15, 95% CI [0.00, 0.30]); as-treated analysis: (β = 0.17, 95% CI [0.03, 0.31]).  No treatment group difference in WLZ/WHZ in the ITT analysis | Low |
| Murty, Rao, & Bamji  2016  (45)  India | Repeated cross-sectional design | Home gardens  Distribution of vegetables seeds/fruits sapling - green leafy vegetables (GLV); and fruits, to families with a pregnant woman or 6- to 24-month-old children  Backyard Poultry purchased by experienced local farmer, raised them and sold to the interested families, one male bird was given free as incentive | Health and Nutrition Education   - Training for teachers and Health activists - Transfer of farm technologies, cooking demo, showing preparation of recipes incorporating vegetables GLV - Open-air slide, sound & video shows - Distribution of educational pamphlets | - Surveys at baseline & endline (project period: July 2011 to July 2014 - 142 mothers with 6- to 24-month-old children | Knowledge, attitude & practice on food consumption; & infant-feeding practices | **Pathways**  *Production*: ↑Vegetable & fruits garden (30% vs. 70%) & poultry rearing (150 HHs at endline) *Knowledge*: ↑ Knowledge on eating more food by pregnant women(P<0.0001) and cause of diseases ↑*Income* from veg (25-30%), sell fertilized egg  **Impact**  *Household* ↑Weekly mean frequency: GLV cooked (P<0.0001)  ↑HH cooking GLV > 3 times (P<0.0001) ↑Egg consumption frequency & quantity (P<0.001) *Children* ↑ Practices on EBF (P<0.0001) & CF (P<0.0001), & ↓pre-lacteal feeding(P<0.0001) ↑Mother's hand washing with soap before child feeding (44%vs 94%) No impact on access to bathroom & latrine No impact on birth weight  ↓ Underweight (P<01), ↑ in monsoon and summer | High |
| Olney  et al.  2009  (41)  Cambodia | Repeated cross-sectional design | - Village model farms to distribute seeds, seedlings, saplings, poultry, and animal, and to train on HFP and nutrition education - Women received homestead food production inputs, training in homestead food production activities | - Nutrition education (mentioned) | - Surveys at baseline (October 2005) & endline (May 2007) - 500 HHs with at least one child < 5 years, or a person living with HIV or AIDS, a disabled person, or an orphan (300 intervention & 200 control in both baseline & endline. | Production, consumption & intake of micronutrient-rich foods  Maternal & child related morbidity symptoms & nutritional status | **Pathways**  *Production*: vegetable production, no impact on animal production or ownership *Income***:** HFP income increase in both groups  **Impact**  *Household:* ↑ Consumption of dark GLVs, no impact on non-dark leafy veg or ASF, ↑ HDDS *Children* ↑ Consumption of egg (diff: 0.7 days/week) no impact on CDDS No impact on Hb or anaemia ↓ Fever prevalence(P<0.05), diarrhoea: no impact No impact on stunting, wasting or underweight, or HAZ, WHZ, WAZ *Women* No difference on micronutrient rich foods consumption , no impact on DDS, Hb, or diarrhoea No impact on weight or BMI | High |
| Olney et al., 2013  (35)  Cambodia | Qualitative design | - VMF to demonstrate the best practices, and ensure inputs availability (seeds, seedlings, and saplings), raise livestock such as chickens and ducks to distribute to beneficiary household, commercially - Agriculture training - Inputs provision | - Village health volunteers linked to local health services, health and nutrition education sessions - Health &nutrition training by health volunteers on: micronutrient-rich foods consumption (including eggs) by women and young children, through recipes and, cooking demonstrations | - Semi structured interviews, FGDs and Observation (Nov,2009 & March 2010) - Program ben. (n = 48), non-ben. (n = 12), & implementers (n = 19),  observation of village model farms (n = 6), & household gardens (n = 36 ben & (n = 12 non-ben) | Themes: income, women empowerment HH consumption,  Child care & feeding practices,  MCHN outcomes | **Pathways**  ↑ *Production* of vegetable, some ↑ production of fruits & poultry  *Knowledge*   Knowledge on BF, food to add in porridge,  safe food preparation, but limited knowledge of complementary feeding, need of more fluids to sick children  ↑Knowledge on micronutrient, limited knowledge on anaemia ↑Income from vegetable, less income from fruits & poultry  **Impact**  *Household/ not specified*: ↑consumption of veg, poultry meat, but limited consumption of fruit and eggs purchased at the market  *Children*: ↑consumption of rich porridge of for children, marginal ↑ in handwashing practices | Medium |
| Olney et al.,  2015  (42)  Burkina Faso | Cluster-Randomized Control Trial  Control (without interventions), Intervention 1(HFP and nutrition BCC implemented by older women leader (OWL) & Intervention 2  (HFP and nutrition BCC implemented by health committee member (HC) | - Input distribution: seeds, saplings, chicks, and small gardening tools - Training on production at demonstration farms | - BCC on essential nutrition actions (women's nutrition, anaemia, iodine intake, prevention of vitamin A deficiency, breastfeeding, complementary feeding, and, care for sick and severely malnourished children) | - Surveys at baseline (Feb-May 2010) & endline (Feb-June 2012) - Mothers of children 3-12 mo (baseline: 1767 HHs,  endline: 1481 HHs) | HB status, anaemia, stunting, underweight, wasting, diarrhoea prevalence, knowledge on IYCF and hand washing, IYCF practices | **Pathways**  (data on production extracted from manuscript & supplementary table 1)  ↑ *Production* of Vitamin A rich fruits & veg (DID: 50.2 kg for OWL, P=0.00, DID: 60.5 kg for HC, p=0.02) and other fruits & veg (DID: 25.1 kg, P=0.02 for OWL, DID: 49.0, P=0.05 for HC) ↑*Knowledge* on need of liquid, and semisolid at 6 mo (DID: 28.2, p=0.00, & DID: 18.1,p=0.00, respectively for OWL, DID: 27.3, p=0.00 and DID: 15.9, p=0.04, respectively for HC) ↑*Knowledge* on washing hands before feeding child (DID: 13.9, p=0.04 for OWL, DID: 22.2, P=0.01 for HC), no change on other attributes of WASH (handwashing before eating, after using toilet, after cleaning child)  **Impact**  *Household*  Marginal ↑HDDS in HC (DID: 0.8; P = 0.07), no impact in group 2 *Children* Marginal ↑Minimum DD among 6-12.9 mo child (DID: 12.6, P=0.08 for HC), no impact on intake of iron rich foods ↑Hb for 3-5.9 mo child (DID: 0.76 +/0.33 g/dL; P = 0.02) & marginal ↑Hb for 3-12.9-month child (DID: 0.51 g/dL; P = 0.06): in HC  ↓Anaemia in HC for 3-5.9 mo child (DID:-14.6 pp, P =0.02), no impact for 3-12 mo child in HC, or, in OWL ↓ Diarrhoea on 3-12.9 mo child in HC (DID: -15.9pp, P=0.00) and OWL (DID:-9.8 pp, P=0.05), no impact on 3-5.9 mo child Marginal ↓wasting in HC for 3-12.9 mo (DID: -8.8 pp, P = 0.08), no impact on stunting or underweight for any groups | Medium |
| Olney et al.,  2016  (55)  Burkina Faso | Cluster-Randomized Control Trial  Control, intervention 1 & intervention 2  Analysis: intervention 1 and 2 versus control | - Dedicating land to women's production - Distribution of inputs and training for production and consumption of nutrient-rich foods and, income generation, including women’s control over income - Inputs: saplings, cuttings, and seeds of nutrient-rich fruits (mango, papaya), vegetables (e.g., orange-flesh sweet potatoes, dark green leafy vegetables, and carrots), gardening tools (e.g., hoes, shovels, and watering cans), and chicks - Training on optimal agriculture and poultry-raising practices | Nutrition   - BCC on nutrition and health by either older women leaders (OWLs) or health committee (HC) members - Topic: Essential Nutrition Actions - Beneficiary visit by community volunteers, i.e., either OWL or HC members   Women empowerment   - Dedicating land for access and control over resources | - Surveys at baseline (Feb-May 2010) & endline (Feb-June 2012) - Mothers of children 3-12 mo (baseline: 1767 HHs; endline: 1481 HHs) | Dietary intake  Dietary diversity  BMI  Underweight  Women empowerment | **Pathways**  *Women empowerment* ↑ total WE score (DID: 3.13+-0.99, P<0.01) ↑Meeting with other women (DID: 1.21+-0.27, P<0.01) ↑Purchasing decision (DID: 0.86+-0.30, P=0.01) Marginal ↑ health care decision (DID: 0.24+-0.12, P=0.05) No impact on spousal communication, social support, decision on family planning or IYCF  Food expenditure: fish or seafood, fruit, meat, or poultry  **Impact**  *Household* ↓ roots & tubers consumption (DID: -4.5, P=0.03) ↑Consumption of fish and seafoods (DID: 18.9, P=0.03) , fruits (DID:22.2, p=0.04), marginal ↑ consumption of meat & poultry (DID: 10.3, P=0.08), no impact on veg, milk & dairy, legumes, nuts & pulses, oils & fats consumption, no impact on HDDS *Mothers* Marginal ↑ DDS (DID: 0.3+-0.18, P=0.08) ↑ Consumption of fruit (DID = 15.8 pp; (P=0.02), marginal ↑ intake of meat/poultry (P=0.08), ↑consumption of cereals & grains (DID: 4.5, P=0.01) No impact on consumption of veg, milk & dairy products, eggs, fish & seafoods, oil & fats, legumes, nuts & seeds and roots & tubers  ↓Underweight [DID = -8.7 pp; P<0.01]  ↑ BMI for underweight women in baseline (coefficient +- SE: b = 0.70 +-0.31), but not in others (coefficient+- SE: b = 0.01+- 0.22) | Medium |
| Osei et al.,  2015  (66)  Nepal | Prospective cluster-randomized control  (2 interventions  & control) | - Inputs One-time distribution of seeds, saplings and chicks, to women receiving nutrition education - Establishment of demonstration farm Demonstration farm establishment to provide ongoing supply of inputs, disseminate agriculture techniques, and share knowledge and skills | - Promote ENA: dietary intake, optimal breastfeeding, complementary feeding), and consumption of iodized salt and foods rich in vitamin A and iron. - Encourage participation in routine public health activities - Children in intervention with EHFP + MNP, also received MNP (60 sachets) | - Surveys at baseline (Sept- Oct, 2010) & post -MNP distribution (Feb/ 2012) - Mothers of children aged 6–9 mo, 335 children at entry | Haemoglobin & anaemia; stunting, wasting and underweight; prevalence of diarrhoea & fever | Pathways not reported  **Impact** (*children*)  No significant ↓on Hb, but difference was higher for EHFP+MNP vs control (4.1 g/L) compared to EHFP vs control (3.6 g/L)  No significant reduction in anaemia (slightly higher magnitude in the EHFP + MNP [-51.5 pp PP] than the EHFP (-48.6 PP) or control (-39.6 PP), with aOR (95% CI) at post-supplementation of 0.52 (0.25–1.12) for EHFP + MNP & 0.69 (0.35–1.36) for EHFP, compared with control. ↓ Diarrhoea for EHFP vs control (2.43pp,95% CI: 2.13-2.73, P=0.010), no impact for EHFP+MNP vs control No impact on fever  No impact on stunting, wasting or underweight | Low |
| Osei et al.,  2017  (43)  Nepal | Unblinded Cluster-Randomized Control Trial  Intervention & Control | - Establish VMF - Training: home garden & poultry production - Inputs provision: seeds, saplings, & locally bred chicks - Women's monthly meeting at Village Model Farm to discuss on agriculture techniques and nutrition | - Social and behaviour change communication (SBCC) on IYCF, cooking demonstrations, use of the EHFP produce, and participation in routine public health services | - Surveys at baseline (Aug,2009) & follow-up (Aug/Sept,2012) - Children 12-48 mo & their mother pairs, 2106 at baseline (treatment: 1055; control: 1051) & 2614 at follow-up (treatment: 1307; control: 1307) | Stunting, underweight, wasting, anaemia among children anaemia and underweight among women | **Pathways**  *Production*: ↑EHFP practice-veg garden & o/r poultry (P<0.05)  **Impact** (*household*): ↓ food insecure HHs (P<0.05) *Children* ↑ colostrum (P<0.05) & EBF (P<0.05)  ↑ MDD, MMF & MAD (P<0.05)  ↓Anaemia among children 0-23 mo (OR [95% CI]: 0.76[0.59-0.98]) No impact on stunting, wasting or underweight *Women* ↓anaemia (OR [95% CI]: 0.62 [0.48-0.82] ↓ Underweight (OR [95% CI]: 0.61 [0.4-0.82] | Medium |
| **HFP: Vegetables and / or fruits; Fish; & Nutrition Education/specific components** | | | | | |  | |
| Michaux et al.,  2019  (67)  Cambodia | Randomized control trial  (2 intervention groups and Control) | - Inputs on EHFP: seeds, seedlings, farming tools, and irrigation equipment - Agricultural training and support - Fish: training on building new ponds or refurbishing existing ponds, technical assistance for polyculture, and inputs (fish nets, fish fry, and fingerlings) | **Nutrition, hygiene and gender equality**   - Interpersonal BCC to on nutrition, hygiene and gender inequality - ENA: nutrition for pregnant and lactating women; micronutrient deficiencies; BF; CF; nutritional care of sick and malnourished children - EHA - Gender transformation | - Surveys at baseline (June 2012) & endline (May/June 2014) - 900 HHs: 300 each for three groups (EHFP & BCC (plant only); EHFP+BCC + Fish; & controls) | Anaemia in women, women's micronutrient status & women and children's anthropometry | Pathways not reported  **Impact**  *Children* Marginal ↑Hb for EHFP+F (DID2.54 (1.43), P=0.076) or EHFP (DID:2.43 (1.42), P=0.088) ↓Anaemia for EHFP (DID: -14 pp, P=0.023), no impact for EHFP +F No impact on stunting, wasting or underweight *Women* No impact on Hb levels or anaemia (for any groups)  ↑RBP concentration in intervention 2 only (DID: 0.34 (0.14), P=0.016) No impact on biochemical & inflammation biomarkers, ferritin, sTfR, serum zinc, AGP, or CRP between groups (for any groups) No impact on serum zinc  No impact on women's underweight based on BMI | Medium |
| **HFP: Vegetables and / or fruits (poultry), Livestock; & Nutrition Education/specific components** | | | | | |  | |
| Kuchenbecker et al., 2017  (56)  Malawi | Randomized Control Trial  (control & intervention groups) | - Approach: Farmer Field Schools, Junior Farmer Field and Life Schools, and farmer field days - Inputs provision: seeds, fertilizer, fruit tree seedlings and livestock - Training and supervision by agriculture extension staffs | - Field testing and application of culturally acceptable and feasible IYCF practices - Nutrition education: 10 sessions weekly, or biweekly on age appropriate food, nutrients, diet, feeding children, food preparation (participatory cooking sessions), WASH | - Surveys at baseline (Aug/Sept 2011) & endline (Aug/Sept. 2014) - HHs with at least one child >23 mo (baseline: 832 HHs -413 control & 419 intervention; & endline: 959 HHs-466 control & 493 intervention) | HAZ   MDD, MAD, and single food groups consumption | **Pathways**  *Production*: No diff: HG garden or arable land  **Impact:** *Children* ↑ Consumption of eggs (P<0.01, IE=9.92%) & groundnuts (P<0.01, IE=14.96%), no impact on consumption of flesh food, fruits & vegetables, dairy products, legumes & nuts other than groundnuts ↑ Minimum Dietary diversity (P=0.01, IE=12.70%) ↑ MAD (P=0.02, IE=11.86%), no impact on MMF  DiD model: CDD IE (B (SE) =0.39 (0.15), p = 0.01; 95%CI 0.09±0.68) Fewer episodes of diarrhoea than control (P=0.02, at endline) but no reduction in prevalence ↑ Access to improved sanitation (P=0.01, control vs intervention at endline), no impact on the access to drinking water source No impact on HAZ, WHZ or WAZ *Mothers*: no impact on BMI | Medium |
| Kumar et al.,  2018  (52)  Zambia | Cluster Randomized Control Trial  (2 intervention groups  Agriculture and gender equity, women empowerment;  Agriculture and gender equity, women empowerment plus nutrition BCC  g& control-standard government services-agriculture (crop) and health | Training, tools and inputs   - Inputs for home gardening (nutrient-rich vegetable), legume and tuber seeds - Inputs on goat and chicken, along with animal husbandry Agricultural tools and training | Nutrition, health and gender  Nutrition BCC interventions   - Mobilization of government's CHV, for IYCF promotion, knowledge and practices, hygiene and preventive health-seeking behaviours - BCC sessions by CHV - Standard government services: (incl. maize focused agricultural extension, ANC visits by CHVs and growth monitoring)   Gender activities   - Group activities, spousal discussion, community sensitizations on gender equality and its importance on nutrition - Use of drama, posters, brochures & radio broadcast - Trial of fuel-saving stoves and provision of fuel trees | - Surveys at baseline (2011) & endline (2015) - Children 24–48 mo of age (2243 children at baseline & 2346 at endline) | Women empowerment; Maternal knowledge on IYCF;  Child morbidity symptoms (fever, cough/cold and diarrhoea); & Child anthropometry: wasting & stunting | **Pathways**  *WE* (for both interventions vs. control) ↑Social capital(DID: 17pp, P<0.001), asset access score(DID: 6 pp, P<0.05) , financial empowerment( DID: 4pp, P<0.05), agriculture empowerment (DID: 6pp, P<0.05)  No impact on decision making power, spousal relationship, perception of gender equality, asset selling, buying power (DID: -0.08, P<0.05) ↑time for agri (P<0.01), reduced time for domestic work & child care (P<0.05) ***Knowledge*** ↑Knowledge on expressing breastmilk (AG-G-BCC: 12.7 pp, Ag-G: 12.1 pp), & BF up to 24 mo (Ag-G-BCC: 14.8pp; AG-G:16.3 pp) ↑Knowledge on timing of introduction of complementary foods for Ag-G-BCC vs control, but not for Ag-G-BCC vs Ag-G; Knowledge on colostrum feeding(-6.4 pp for AG-G-BCC and -5.30 pp for Ag-G), giving others liquids or food during first 6 mo (-13.4 pp for Ag-G-BCC, -12.4 pp for Ag-G) & continuation of BF when mother is ill (Ag-BCC-G vs control by -9.9)  Knowledge on complementary feeding (score) higher for Ag-G-BCC vs control (DID: 0.68 pp)  **Impact** (*children*) ↑MMF: Ag-G-BCC vs. Ag-G(DID: 11.60), no impact on MDD, MAD No differential impact on consumption of iron rich foods, No impact on BF (score)  No impact on fever for any groups ↓Diarrhoea prevalence for Ag-G-BCC vs Ag-G (DID: -7.7, P<0.05) ↓Cold/Cough prevalence: AG-G-BCC vs Ag-G (DID: -10.8, P<0.01) ↑ WHZ score: Ag-G vs control (DID: 0.38, P<0.05) No impact on HAZ, wasting or stunting | High |
| Rosenberg  et al.,  2018  (39)  Zambia | Randomized Control Trial (intervention & control) | Homestead gardening, food crop production and animal rearing | - Nutrition BCC: IYCF, health seeking behaviour and hygiene | - Surveys at baseline (2011) & endline (2015) - HHs Baseline: 3044 (2003 intervention & 1041 control); endline: 3536 (2456 intervention & 1080 control). - Children (6-24 mo) Baseline: 1524 (938 treatment & 586 control); endline: 1343 (841 treatment & 502 control) | Household economic well-being Household agriculture Individual dietary diversity, household food access and food security | **Pathways**  *Production*  agricultural production diversity (extensive margin on types of crops grown per year: and no. of food groups, intensive margin, e.g., the no. of mo in which a food group is harvested)  ↑ Production groundnuts, rape (GLV), tomatoes (20pp), no apparent increase in meat and fish ↑Cotton seeds production (not targeted by project) (P<0.01)  *Income* ↑ Selling from non-food agri -cotton & crops (groundnuts, rape, tomatoes, sweet potatoes)  **Impact**  *Household* ↑ Total no. of food groups consumption ↑Consumption of pulses, legumes & nuts, meat & fish, no effect: egg or vegetables/fruits No impact on hunger scale & food insecurity *Children* ↑Consumption of pulses, legumes & nuts, no effect: egg or vegetables/fruits No impact on total foods groups consumption, or dietary diversity *Women* No impact: DDS or total food group consumption | High |
| Reinbott  et al.,  2016  (57)  Cambodia | Randomized Control Trial  (intervention & control) | - Mobilize existing farmer groups, in a FFS, on either rice or chicken or vegetables or cash-crop - The FFS curriculum: field days and sessions on family nutrition.  Farmer business schools: link farmers to each other & to local markets - Agricultural fairs - Input: voucher to farmer for related purchases (fertilizer, seeds, tools, etc.) or kitchen equipment, obliged to pay back 60% of the value after receiving income from the harvest | - Nutrition education sessions: continued BF, CF, dietary diversity, feeding a sick child, responsive feeding, family nutrition and hygiene practices - Use of national nutrition education materials - Cooking demonstrations: educational posters, soap and kitchen to the participants. | - Surveys at baseline (Aug 2012) & impact (Sept/Oct 2014) - HHs with at least one child < 23 mo Baseline: 1028 HHs Impact: 1076 HHs | Children's dietary diversity;  Children’s nutritional status | **Pathways**  *Production*: No significant improvement on ownership of animals access to fruit or HG  **Impact** (*household***):** no impact on HDDS  *Children* ↑ CDDS (B= 0·52, SE(B) =0·18; 95% CI 0·17, 0·87,P= 0·005) ↑ MAD (P=0.004), no impact on MMF ↑ consumption of VA rich foods (TE: 0·16(0·05), P=0·003, 95% CI: 0·06- 0·26); & other fruits & veg (TE: 0·17 (0·06), P= 0·003, 95% CI: 0·06,-0·28)  No impact: consumption of flesh foods, egg, dairy No difference on introduction of semi-solid foods at 6 mo at endline No difference on access to sanitation facility or drinking water source at endline No difference on fever, diarrhoea or ARI at endline  No difference on HAZ, WHZ or WAZ at endline | Medium |
| **Orange Fleshed Sweet Potato (OFSP); & Nutrition Education/specific components** | | | | | |  | |
| de Brauw  et al.,  2015  (68)  Mozambique | Randomized control trial Intervention and control | - Distribution of multiple OSP varieties of vines - Training or reinforcement of growing techniques to farmers - Participation in extension meetings on growing OSP - Agriculture extension - Volunteer promotor in each farmers group for OSP production support to farmers | - Train and inform nutritional benefits of OFSP & other VA sources - Dissemination through: group trainings, community theatre sessions radio spots, billboards and other advertising. - Nutrition extensions worked with nutritional promoters with groups of women | - Surveys at baseline (2006) & endline (2009) - HHs with women of child-bearing age & children < 5 y - Children 6-35 mo selected - Socioeconomic survey: baseline: 703, endline: 628; - dietary intake: baseline: 441 & endline: 409 | Mean Micronutrient Density Adequacy  (MMDA) Dietary diversity score anthropometry | Pathways not reported  **Impact** (*children*)  ↑ VA density in diet, more significant for ag, vines + extension ↑ dietary diversity, higher diversity for more intense participation in nutrition (coef is 0.4 or higher) ↑ MMDA for treatment children (P=0.010) ↑ MMDA for HHs & women with nutrition promoters (4.2 percent)  ↑ Dietary diversity score (P=0.020) higher for ones with nutrition, promoter (coef. 0.610, P<0.01) and ag+ vines +extension (coef. 0.402, P<0.01) No impact on WHZ score | Medium |
| de Brauw et al.,  2018  (37)  Mozambique Uganda | Randomized Field Experiments trial (2 interventions & controls) | - OFSP vine distribution - Training on growing | - Nutrition training | - Surveys at baseline & endline - Mozambique Baseline: 703 HHs, endline: 628 HHs - Uganda Baseline: 1176 farmers  Endline: 1,116 farmers | OSP adoption, Nutrition knowledge Vitamin A intakes | **Pathways**  *Production*  ↑ OFSP adoption, share of OFSP& total area for OFSP(P<0.01) for both, Mozambique & Uganda ↑ OFSP adoption in Mozambique (IT vs control: 65.7%, MT vs control: 69.2% more likely); Uganda: (IT vs control: 61.7%, MT vs control: 57.9% more likely) ↑ Share of OFSP: Mozambique: (IT vs control: 62.2%, MT vs control: 58.7% more likely);Uganda: (IT vs control: 11.0%, MT vs control: 42.8% more likely)  *Knowledge* ↑ Knowledge no. of facts on vitamin A & OFSP as a source of vitamin A ↑ No. of mothers naming OFSP as a source of vitamin A in Uganda (increase by 45.4%) & Mozambique (increase by 24.4%) (IT & MT) Knowledge has no significant effect on adoption (coeff. 0.049 in Mozambique, coeff 0.040 in Uganda)  **Impact** (*children*): ↑ dietary intake of vitamin A (IT vs control, & MT vs control), significant change (level of significant values not provided) | Medium |
| Girard et al.,  2017  (44)  Kenya | Longitudinal cohort study (intervention &control) | - Integrate agriculture and nutrition interventions into antenatal care (ANC) and postnatal care (PNC) health care services - Inputs: vouchers for OFSP planting materials Enhanced agricultural extension | - Enhanced nutrition education - PLW received enhanced nutrition counselling at health clinics, and were linked with community-based maternal support groups - Integration into ANC and PNC health services | - Follow-up of women from their first ANC visit to 9 mo of postpartum, - 3015 pregnant and lactating women | Nutrition and health-seeking knowledge, food security, dietary  patterns, & anthropometric measurements | **Pathways**  *Knowledge*: ↑ Knowledge on nutrition & health (DID: 1, 95% CI: 0.1-1.9P<0.05) & vitamin A (DID: 0.8, 95% CI: 0.4-1.2 P<0.01), no impact on knowledge on IYCF  **Impact** (*women*) ↑ Consumption of VA rich fruits & veg (DID: 0.4, 95%CI: 0.2-0.6, P<0.01) ↑ Intake of B-carotene (P=0.01) & vitamin A (P=0.01) No impact on energy intake, or WDDS ↑Vitamin A adequacy (P<0.001), met vitamin A DRI and EAR (P<0.001) ↓Odds of low RBP (P=0.01)but no impact on mean RBP concentration  ↓ Odds of anaemia in late pregnancy, no impact for the overall, no impact on Hb No significant impact on mean MUAC | Medium |
| Hotz et al.,  2011  (69)  Mozambique | Randomize, controlled effectiveness study  (2 intervention groups: low intensity; high-intensity; & control group) | - Inputs: distribution of OSP vines at first year (additional vines made available for purchase) - Training for improved production practices, marketing and product development for OSP traders, urban and rural market development | Demand creation/behaviour change component   - Education on maternal and child health and nutrition topics - Campaign for the general public to raise awareness of the benefits of OSP, using drama, field-day events, and radio spots and programs | - Surveys at baseline (Nov-Dec 2006) & follow-up (May-June 2009), - HH: child 6–35 mo - Baseline: intervention 1: children-149; women-149 Intervention 2: children 145; women-146; Control: children-146; women-146 - Endline: Intervention 1: children- 229; women-134; Intervention 2: children-223; women-129; Control: children-224; women-130 | Change in vitamin A intake  Change in OSP intake  Prevalence of inadequate vitamin A intake | Pathways not reported  **Impact (***children and women*) ↑ OFSP intake intervention1 vs control (P<0.01) & intervention2 vs control (P<0.01)  ↑ Vitamin A intake from OFSP for intervention 1 vs control, or intervention 2 vs control (P<0.01 in adjusted model)  Lower change in Niacin intake in grp 2 among 3-5.5 y children relative to control (-1.16mg/d, P<0.05) No impact on children's intake of protein, lipid, ca, fe, Zn, vitamin C, thiamin, riboflavin or vitamin B12 ↓Prevalence of inadequate vitamin. Vitamin A intake among children (12-35 mo) & women (P<0.05) | Medium |
| Hotz et al.,  2012  (70)  Uganda | Randomized, controlled effectiveness study (2 intervention groups: intensive; program (IP); reduced program (RP); & control group) | - OFSP vines distribution - Agriculture sessions | - Nutrition and health sessions | - Surveys at baseline (2007) & follow up (2009) - Size for analysis   -interventions 1& 2 vs control: OSP & vitamin A intakes: children (6–35 mo: n = 265; & 3–5 y: 578) & women (n = 573)  -intervention 1 vs. control: vitamin A status of 3-to 5-yrs old children (n=891) & women (n=939) | Changes in OSP intake & vitamin A intake and serum retinol | Pathways not reported  **Impact** *(children and women)* ↑ OFSP intake IP-control, RP-control (P<0.01)  ↑ Vitamin A intake IP-control, RP-control (adjusted, P<0.01), OSP (P<0.01)  ↓Prevalence of inadequate vitamin A for children 6-35 mo (> 30 pp, P<0.01), no difference by intervention groups, or for children 3-5 y No impact on prevalence of infection based on elevated CRP for children 3-5 y or women (IP vs control) ↓ Prevalence of low serum retinol for children No impact on serum retinol for women ↓Prevalence of inadequate vitamin A for women (> 25pp, P<0.01) with no difference in treatment groups | Medium |
| Jones & De Braw  2015  (71)  Mozambique | Cluster Randomized Control Trial  (intervention & control | - Distribution of multiple OSP varieties of vines - Training or reinforcement of growing techniques to farmers - Participation in extension meetings on growing OSP - Agriculture extension (volunteer promotor in each farmers group for OSP production support to farmers) | - Train and inform people about the nutritional benefits of consuming OFSP and VA sources - Sources of dissemination: group trainings, community theatre sessions, radio spots, billboards and other advertising. - The nutrition extension workers worked with several nutritional promoters per village, with groups of women | - Surveys at baseline (2006) 7 endline (2009) - 1,321 children < 5 y (540 at baseline & 781 at endline) | Diarrheal disease prevalence & duration | Pathways not reported  **Impact** (*children*) ↓Prevalence of diarrhoea among children under 5 y of age (-11.4 pp, 95% CI: 2.0-20.8), children under 3 (-18.9pp, 95% CI: 6.6-68.3) Children eating OSP were less likely to experience diarrhoea (15.9pp, P<0.01, (95% CI 5.9–25.9)  ↓Duration of diarrhoea among children < 3 y of age reduced diarrhoea duration by 1.3 days (27%), P<0.01, 95% CI: 0.4-2.2 days) | Medium |
| Low et al.,  2007  (36)  Mozambique | Quasi-experimental prospective longitudinal  (Intervention & control) | - Integrated farmer extension on production methods, storage, and commercialization of OFSP | - Nutrition extension activities - Group education sessions (9-12 sessions) on infant and young child feeding and hygiene practices through visual aids, recipe demonstrations, and role playing that conveyed messages regarding. | - Surveys at baseline (May-June 2003), follow up (Nov 2003-Jan 2004) & endline (Nov 2004-Jan 2005) - Households with children 4-38 mo followed over 2 agriculture cycles (498 intervention & 243 control) | Nutrition knowledge  Production & sale Child dietary intake Serum retinol concentration Child anthropometry & morbidity | **Pathways**  *Production*: ↑Production of sweet potato (median:73 kg to 127 kg)(P<0.001)  *Income*: ↑Sweet potato selling (13% to 30%, P<0.001) Perceived change in food price: OFSP was cheapest source of vitamin A in market (1 cent for 700 RAE) *Knowledge* on nutrition: ↑Scores (12 point) for intervention women (8.1) than control women (4.3) , P<0.001; and ↑ scores (12 point) for intervention men (6.3) than control men (4.7) , P<0.001  **Impact** (*children*) ↑Consumption of OFSP (P<0.001) ↑Intake of vitamin A (P<0.001, 426 mg RAE in intervention vs 56 in controls), energy (P<0.001), protein (p=0.04), B-carotene (P<0.001), thiamin (P<0.01), riboflavin (P<0.001), vitamin B 6(P<0.001) vitamin C (P<0.00), folate (P<0.01), iron (P=0.02), zinc (p=0.05), not change in the intake of calcium or zinc  ↑ Serum retinol concentration (P<0.01, double diff 0.076 mmol.l)) ↓ Prevalence of low serum retinol(↓by 10% in intervention, P<0.01) No impact on child morbidity based on symptoms or elevated CRP  ↓ Prevalence of wasting (higher prevalence in controls (6 vs 3, P=0.03) & underweight (higher prevalence in controls, 34 vs 24, P<0.01) No impact on prevalence of stunting | Low |
| Low et al.,  2007  (46)  Mozambique | Quasi-experimental intervention design (intervention & control) | - OFSP inputs- vines distribution - OFSP training: agronomics, bio multiplication, storage techniques - OFSP market development: training to bakers & doughnut makers on processing, market price incentives in year 2 including the concept of quality grades promoting higher price for first quality OFSP | - Demand creation on vitamin A rich foods and OFSP - Knowledge on nutrition & hygiene: IYCF, diversified diet, hygiene practices, BCC/mass awareness on nutrition | - Baseline survey (Jan 2003–Mar 2003) & follow up surveys, F1 (Nov 2003-Jan 2004), F2(Aug 2004-Oct 2004), F3 (Nov 2004-Jan 2005) - Households with children 4-38 mo followed over 2 agriculture cycles (498 intervention & 243 control) - Diet diversification was limited by difficult agroecological conditions and low purchasing power. | Food Frequency, Dietary Intake,  Dietary Diversity | **Pathways** *Production*: ↑plot size & agronomic practices, yields, substitution of WFSP, OFSP production, preservation, storage & processing Income: ↑OFSP sell (30% from 13%)  ↑ OFSP purchase, ↑expenditure on vitamin. A rich food in harvest season(P<0.05) but no impact on mean monthly expenditure  ↑ job opportunities, highest net returns to labour on bread & doughnuts Food price(perceived): OFSP cheapest source of vitamin A in 2004 *Knowledge* ↑ Nutritional knowledge score for women (8.1 vs 4.3, P<0.001), and men (6.3 vs 4.7, P<0.001) ↑ Knowledge on Vitamin A (84%) incl. fighting against disease (59%) & protecting eyes (34%), nutrition &/ or health (88%), sources of vitamin A (66% women vs 55% men)  **Impact** (*children***)** ↑Consumption of OFSP (55% vs 4%, P<0.001dark green leaves (60% vs 46%, P<0.001), groundnuts (12% vs 3%, P<0.001), beans (42% vs 31%, p<0.01) & fruits; no effect: chicken, fresh fish; ↑ Intake of vitamin A. A(P<0.001), energy (P<0.001), B carotene(P<0.001), riboflavin(P<0.001), vitamin B6(P<0.001), vitamin C (P<0.001), thiamin(P<0.01), niacin(P<0.01), folate (P<0.01), iron (P=0.02),  No significant impact on intake of vitamin B12, calcium, and zinc ↑ Dietary diversity at 2^nd^ year (32% vs 9%, P=0.001) | High |
| **Livestock & Nutrition Education/specific components** | | | | | |  | |
| Kassa et al.,  2003  (72)  Ethiopia | Cross sectional surveys (2 times) and qualitative methods | - Identification of women's groups - Training to women's groups on husbandry of dairy goats - Provision of first local and crossbred dairy goats through revolving credit schemes | - Nutrition education (mentioned) | - Baseline (803 HHs) and  formative (228 HHs) surveys - Nutritional status study: 1,338 children < 5 y of age - Focus group discussions | Anthropometric measures, dietary assessment,  Vitamin A intake | **Pathways**  *Production***:** ↑ownership of goat(P<0.05), no impact on sheep, chicken  **Impact**  *Children* ↑ Milk consumption by adults as hoja, resulting in less consumption among children No impact on mean consumption frequency of animal origin vitamin A rich foods (for all three groups)  ↓ Stunting based on BMI (group 1: 38.7%, 2: 32.4%, 3: 47.9%) ↓ Underweight based on BMI (group 1: 41.9%, 2: 31.0%, 3: 50.7%) No impact on wasting based on BMI | High |
| Le Port et al.,  2017  (50)  Senegal | Cluster Randomized Control Trial  (intervention & control) | - Dairy value chain - Milk collection by factory from producer - Micronutrient fortification (iron) of yoghurt: 80 g sachet of yoghurt mixed with millet grains and fortified with 2.1 mg of iron-EDTA - Households fulfilling contract in received one sachet of MNFY per child 2-5 y | - BCC intervention: group sessions, home visits for remote households, social mobilization (theatre plays), and nine radio spots. - Messaging focused on essential nutrition actions (ENA): IYCF, micronutrients, dietary diversity, the importance of consuming iron-rich, or fortified foods, and anaemia | - Surveys at baseline (Jan 2013), follow ups: F1 (April, 2013), F2 (Sept 2013) & endline (Jan 2014) - HHs with children (24 to 59 mo of age at baseline:  Intervention: 204 children  Control: 245 children | Household food security  Maternal knowledge Hb status Anaemia | **Pathways**  *Knowledge*: ↑Knowledge on health consequences of anaemia & dietary sources of iron (P<0.001)  **Impact** *(children)* ↑Hb concentration, more in intervention group (0.55 g/dL, 95%CI:0.27; 0.84), greater impact for boys (0.72 g/dL, 95%CI: 0.34; 1.12), compared to girls (0.38 g/dL, 95%CI (-0.03; 0.80) No impact on prevalence of anaemia or severe anaemia | Medium |
| **Farm crop diversification & Nutrition Education/specific components** | | | | | |  | |
| Kerr, Berti & Shumba  (73)  2011  Malawi | Quasi-experimental design  (intervention & control) | - Legume intercrops: peanut and pigeon pea; soybean and pigeon pea; pigeon pea intercropped with maize; velvet bean rotated with maize; and Tephrosia intercropped with maize. | - Gender and nutrition education: intergenerational activities through monthly group discussions on early child feeding and sharing of household resources | - Baseline survey & 9 follow-up surveys - Date (no.): Nov 2001(276), March 2002 (401), Aug 2002 (493), Jan 2003 (644), March 2004 (562), Oct, 2005(348), March, 2006 (304), May 2006(308), Oct 2006 (128) & March 2007(374) | Child growth | Pathways not reported  **Impact** (*children*) ↑Weight for age z score; longest involvement villages (increase over initial conditions of 0.6 from -0.4 (SD 0.5) to 0.3 (SD 0.4)  ↑Weight for age z score for most intense involvement: (increase over initial conditions of 0.8 from -0.6 (SD 0.4) to 0.2 (SD 0.4) | High |
| Kerr & Chilanga  2016  (74)  Malawi | Quasi-experimental design with qualitative methods  (Participatory, recipe day activity) | - Based on Eco health model (interrelationship of ecological, social and economic factors to improve human health) - Testing of organic methods - Legume intercropping (such as pigeon pea and groundnuts) rotated with maize to improve soil fertility and dietary diversity, added tubers such as cassava and sweet potatoes. | Nutrition and gender   - Community-based participatory nutritional education to promote healthy complementary foods' consumption, share skills on preparing diverse recipes, and encourage more equitable household gender roles - Men and women came together for recipe preparation and sharing, nutritional skills and equal gender role in child care and cooking, division of labour and decision-making | - Key information interviews (May & Aug2012) - 120 interviews (before, during & after intervention) - Size: 30 married couple HHs with children < 5 y; 7 key informant interviews with village heads & elders | Gender relations | **Pathways**  *Knowledge*: ↑ knowledge on nutrition and health *Women empowerment*: ↑resource access, gender norms  **Impact** (*children*) ↑ Consumption of variety of foods | Medium |
| **School garden & Nutrition Education/specific components** | | | | | |  | |
| Agdeppa  et al.,  2019  (49)  Philippines | Cluster randomized controlled design  (school 1 & school 2) | School Gardening   - Provision of inputs/garden implements to school - Training to teachers on gardening - Use of garden health and resilience approach - Daily produce from the gardens, were used in the supplementary feeding | **Nutrition education** Developed 10 modules on health and hygiene, nutrition, and gardening for use during nutrition education sessions.  **Educating parents**   - Lectures on nutrition education during parent–teacher meetings, card day - Mothers involved in meal planning, preparation, and cooking - Mothers encouraged to visit school garden and read materials on nutrition at school   **Supplementary feeding**   - Menu: indigenous vegetables from the school garden, complemented by normal rice in school 2 and iron fortified rice in school 1. - Feeding was done for 120 days | - Survey including KAP of mother - 80 underweight or anaemic school children aged 6 to 8 years in each of 2 schools - Mothers participated in KAP survey | Micronutrient status: Hb & anaemia  Growth outcomes: stunting, underweight & wasting | **Pathways**  *Production*:↑ types of veg used in feeding program (7 to 10, P=0.000) & amount of veg harvest for supplementary feeding for 120 days: 68-110.28 kg, PhP 3,282.90 to 5,568.63; no impact on backyard gardening *Income*: (perceived)↑school income from production, HHs purchase fortified foods  *Knowledge and Attitude/preference*↑ Knowledge on negative consequence of worm infestation, (33.3% to 60.6%, P = 0.035), no difference on knowledge on basic nutrition, personal hygiene, food fortification, good nutrition  ↑ Attitude on right way of preparing veg for cooking(P=0.002), preparation of nutritious food (18.2-42.4%, P = 0.039)  ↑ Convincing children to eat veg (36.4% to 69.7%, P=0.003) ↑ Post exam lessons on nutrition (P=0.000) (incl. complementary feeding, veg. preparation), food fortification, hygiene & health, no impact on the knowledge on food needed by school children ↑Attitude on meal preparation (18.2% to 42.4%, P = 0.039) & cooking veg. (63.6% to 93.9%, P = 0.002)  [The knowledge & practices of the mothers significantly related to the weight of the children (P < 0.05)]  **Impact**  *Children* ↑ Mean Hb level in school 1 higher (0.49 ± 0.99) than school 2 (0.12 ± 0.79), (P = 0.032) ↓ Prevalence of anaemia in school 1 from baseline to endpoint(65.8% to 47.9%, P = 0.000)↓Prevalence of underweight in school 2 (56.2% to 34.2%, P = 0.002)  ↓ Prevalence of stunting in school 1 (43.8% to 26%, P = 0.004)  No impact on wasting ↑ Weight gain in School 1 (1.33 ± 0.72, P = 0.0134)  *Mothers* ↑ Purchase of fortified foods (51.5% to 93.9%, P = 0.000) ↑Practice of washing veg before use (27.3% to 87.9%; P = 0.000) | Medium |
| Erismann  et al.,  2017  (58)  Burkina Faso | Cluster Randomized Control Trial  (intervention& control schools) | Gardening   - Provision of seeds and gardening tools to school - Agricultural trainings to 12 teachers and four school directors | - **Nutrition and WASH interventions** - Installation/rehabilitation of WASH facilities: latrines, water pumps, stations and toolkits to make soap, and safe drinking water stations in classrooms. - Training on nutrition and hygiene to teachers and school directors - Treatment of anaemia or intestinal parasites in children | - Surveys at baseline (Feb/March 2015) & endline (March 2016) - 8–15 years school children baseline: intervention- 193; control- 92; endline: intervention- 176; control- 184 | Helminths & intestinal protozoa, faecal contamination children’s status of Hb, anaemia & anthropometry (weight & height) | Pathways not reported **Impact** (*children*)  ↑Safe handwashing practices before eating and use of latrines at schools significantly higher in the intervention schools at end-line (OR = 6.9, 95% CI = 1.4–34.4, and OR = 14.9, 95% CI = 1.4–153.9, respectively).  No impact on HH water quality parameter  ↓Intestinal parasitic infections higher on intervention schools (odds ratio [OR] of the intervention effect = 0.2, 95% [CI] = 0.1–0.5) but no impact on helminth infections No impact on anaemia reduction, weight gain, height gain, underweight or stunting | Medium |
| Schreinemachers et all., 2017  (51)  Nepal | Cluster Randomized Control Trial  (intervention & control schools) | School garden   - Cultivation of nutrient-dense vegetables by the schoolchildren by parents' support - Training to teachers on school garden.  Provision of seeds of 14 different vegetables, gardening tools, fencing material and other equipment such as water tank for irrigation - Set up of a polyhouse to raise vegetable seedlings. - Distribution of packets of seed to children for replication at home - Teaching on gardening (along with nutrition and WASH) to school children | **Nutrition, health and WASH**   - Teaching on nutrition and WASH, to school children on health, environment and agriculture. - Behaviour change reinforcing activities: poster displays and the distribution of handouts about nutritious food and hand washing. - Parents received a briefing about the school garden project. | - Surveys in 2 school years (2014 & 2015) - School children of grades 6 & 7 (10-15 y old) from 30 schools - Year 1: intervention- 429; control- 846; & Year 2: intervention- 369; control- 416 | Knowledge on food, nutrition & agriculture; children's preference of; children's consumption; & HAZ score | **Pathways**  *Knowledge/attitude(children) (2^nd^ year)*  ↑ %. fruits & veg correctly named (TE(SE): 12.8(2.4), P<0.001 **↑** knowledge on sustainable agriculture (ATE(SE): 16.7(2.2), P<0.001), food, nutrition & WASH (ATE(SE): 14.6(3.0), P<0.001 ↑ preference of fruits & veg (ATE(SE): 19.1(3.1), P<0.001 **Impact** (*children*)  No impact: consumption of fruits & vegetables No impact on HAZ score | Medium |
| Schreinemachers et al., 2017  2017  (53)  Bhutan | Randomized control trial (intervention & control) | School garden   - Cultivation of nutrient-dense vegetables by the schoolchildren by parents' support - Training to teachers on school garden.  Provision of seeds of 14 different vegetables, gardening tools, fencing material and other equipment such as water tank for irrigation - Set up of a polyhouse to raise vegetable seedlings. - Distribution of packets of seed to children for replication at home - Teaching on gardening (along with nutrition and WASH) to school children - Teachers visited the parents at home and encouraged production | **Nutrition, health and WASH**   - Teaching on nutrition and WASH, to school children on health, environment and agriculture. - Behaviour change reinforcing activities: poster displays and the distribution of handouts about nutritious food and hand washing. - Parents received a briefing about the school garden project. | - Surveys at baseline (2014) & endline (2015) - 9-15 y old school children (grade 3-6)  Baseline: intervention-260; control-265  Endline: intervention-259; control-248 | Children's knowledge on production & food, nutrition & WASH; Vegetable consumption, HAZ score | **Pathways**  *Knowledge*/attitude: ↑ awareness on % of fruits & veg correctly named (ATE(SE): 17.87(6.44), P<0.01), **↑** knowledge on sustainable agriculture (ATE(SE): 15.20(6.86), P<0.05  No impact on knowledge on food, nutrition or WASH ↑ Preference of fruits & veg (ATE(SE): 9.48(3.99), P<0.05 *Income*: selling to school meal program at a low price, if no canteen, distributed to children & teachers for HH consumption, selling to community occasionally **Impact** (*children*) ↑ Consumption of veg (ATE(SE): 11.66(6.15) , P<0.05), no impact on consumption of fruit  ↑ Consumption of variety of veg & fruit for children with home garden No impact on HAZ score | Medium |
| **Vegetable production integrated with early Child Development (ECD) & Nutrition Education/specific components** | | | | | |  | |
| Gelli et al.,  2018  (38)  Malawi | Randomized Control Trial  (intervention & control) | Use of CBCC gardens as a demonstration site for communities   - Training for parents, CBCC Management Committee representatives, farmers, and community agents - Training topics: land preparation, selection of nutritious crops, agriculture production techniques, pest and disease management, manure-making and application, harvesting, storage, processing, and chicken rearing - Support to village savings and loans groups to start home gardens and help communities purchase supplies for CBCC meals. - Agriculture training on nutritious food production, including a traditional variety of orange maize, and biofortified orange-fleshed sweet potato, legumes and nuts, and green leafy vegetables training on care for chickens. - Inputs to participating households- seeds and 10 chicks | **BCC on nutrition**   - BCC and training in nutritional needs of infants and young children, year-round meal planning and preparation, food storage, hygiene, waste disposal, and monitoring of meal provision. - Nutrition training by government staffs to CBCC Management Committee members, CBCC caregivers (teachers) - Recipe development by parents at CBCC, and replication at home - Monthly follow-up visits by government staffs and project staffs | - Surveys at baseline (December 2015) & endline (December 2016) - 1248 preschool children aged 36-72 mo |  | **Pathways**  *Production*: ↑crop production diversity score(DID: 0.71, P<0.001), crop production variety score (DID: 2.14, P<0.001), production of OFSP (DID: 4.32, P<0.001), production of soya beans (impact: 1.45, P<0.001), reduced production of brown bean (DID: -0.90, P<0.05), ↑pigeon peas (DID: 4.86, p<0.05), groundnuts (DID: 3.38, P<0.05) ↑Production of eggs (DID: 3.44, P<0.05) & ownership of chickens (DID: 1.16, P<0.05) No effects on household expenditures *Knowledge* ↑Care giver's broad IYCF knowledge  ↑ Caregiver's knowledge on: early BF (DID: 7.9, P<0.05), colostrum feeding (DID: 10.6, P<0.001), avoiding water for first 6 mo (DID: 13.5, P<0.05) No impact on knowledge on exclusive BF, introduction of CF at 6 month ↑ Food groups knowledge (DID: 0.77, P<0.001) *System strengthening* ↑ No. of days CBCC open (DID: 0.29, P<0.05), ↑ no. of days meal provided on CBCC (DID: 10.6 pp., P<0.05), centre provided meals (DID: 10.6pp, P<0.05) marginal ↑ CBCC enrolment (DID: 4.6 pp., P<0.10), no impact on attendance  **Impact** (*children*) ↑Dietary intake of food quantity (DID: 153g, P<0.001), energy (DID: 294kcal, P<0.001), protein (DID: 8.12 g, P<0.05), iron (DID: 1.64 mg, P<0.05), zinc (DID: 1.09mg , P<0.05), vitamin C (DID: 19.72 mg, P<0.05), vitamin B 6(DID: 0.26 mg, P<0.05), vitamin B 12 (DID: 0.31 mg, P<0.05),  No impact on dietary intake of vitamin A /RAE  No differences between girls & boys on dietary intake of vitamin A/RAE ↑ Individual DDS (DID: 0.36, P<0.001), ↑ Mean DDS (DID: 0.31 pp , P < 0.05; DDS=3.24 in the intervention vs 2.93 in the control group) ↑ Achieving MDD by younger siblings (39% in the intervention vs.28% in control ; mean ± SE difference: 0.11 ± 0.05; P < 0.05) ↑ Individual food variety (DID: 0.55, P<0.001) ↓Stunting among 6-24 mo(DID: -17 pp, P<0.05)  but no impact in preschools (36-72 mo) ↑ HAZ score in 6-24 mo(DID: 0.44, P<0.05)  but no impact in preschools (36-72 mo) No impact on underweight or WAZ score or wasting or WHZ score | Medium |
| **Grain banks & Nutrition Education/specific components** | | | | | |  | |
| Roche et al.,  2017  (75)  Ethiopia | Qualitative design | - Distribution of milled flour at grain bank, using locally available ingredients. - Production of the complementary food at the grain bank through cleaning; roasting and dehulling of the cereals and legumes; mixing and milling of the cereals and legumes in 3:1 ratio; and addition of sugar. - Training and guidance on safe storage and processing of the ingredients, and flour at the grain bank | **Nutrition and WASH**   - Mothers made porridge with the grain bank flour to improved timely introduction of complementary foods- entry point for IYCN - HEW recommended mothers to add other available ingredients to the grain bank flour to cover all micronutrient - Training and guidance on safe and hygienic preparation at home | - Semi-structured interview & focus group discussions (Aug-Nov 2013) - 51 Key informant interviews & 33 FGDs -237 participants of FGD |  | **Pathways**  *Production*: ↑Production of processed grain banks flour (safe & quality flour-diversity of ingredients)  *Income:* Income, purchase of complementary food *Knowledge*: ↑Mother's knowledge on complementary feeding, change mother's perception on the benefit of grain bank food, & change grandmother's perception on the importance of IYCF  *Women empowerment*: ↑time saving  **Impact** *(women and children)* Flour consumption by pregnant & lactating women, elderly & sick person Complementary feeding practices Perceived decrease in prevalence of malnutrition | Medium |
| **Financial support/credit for agriculture & Nutrition Education/specific components** | | | | | |  | |
| Kalavathi et al.,  2010 (76)  India | Follow-up study  (before & after project) | - Disbursement of USD 10,000 to communities as microcredit, to be utilized for intercropping for nutrition gardening, nursery establishment, livestock rearing, production of high value products, mushroom production and azolla cultivation - Selection of suitable intercrops by community (cash and food crops - Training of community on the production of high value products, like baby food and other nutritional foods - Nutrition gardening, livestock integration and mushroom production - Effective recycling of coconut and other crop wastes through vermicomposting | - Nutrition education - Nutrition gardening | - Before and after project surveys Survey dates N/A Project duration: 2005-2008 - 100 active members- Not specified | Change in poverty status & income Change in food & nutrition security | **Pathways**  *Production*: homestead backyard gardening (41% to 87%), livestock/fish/ poultry rearing (32% to 64%) (sig. level not provided) *Income:* Income from intercrops (↑4 times), livestock (↑ 6 times) & processing (↑33 times), ↑average annual per capita income (USD 140 to USD 320) Poverty reduction (95% below 1USD/p/d to 56%), ↑ coping mechanisms on food security (↓in borrowing money for food: -55%) **Impact** HH  ↑Food processing (55% to 85%) (sig.., level not provided)  ↑Food security (67% pre-project vs 96% post-project) ↑ Perceived nutrition security  ↑Fruits consumption frequency (adults: 156%, children, 111%) ↑Egg consumption (children: 130%) Vegetable consumption: (38%)  Consumption of milk, milk products, meat and fish  ↑ Dietary diversity of adults ↑ Children's Dietary diversity | High |
| **Mixed interventions: integrated food and livelihood interventions** | | | | | |  | |
| Fanzo et al.,  2010 (77)  Kenya | Cohort | School-based interventions   - home-grown school meals, gardens and nutrition activities after school - deworming campaigns.   Household- and community-based interventions   - Subsidized seed & fertilizer for agricultural productivity - introduction of high-value crops; agro-processing initiatives; and microfinance programmes to stimulate small-business development homebased fortification and proper food storage techniques. | Nutrition and health   - Complemented by a CHW programme to promote EBF, CF - Clinical interventions: VA supplementation, treatment of SAM, growth monitoring, treatment of MAM through local or Instaflour feeding maternal health: ANC, institutional delivery - School-based: gardens and nutrition activities, deworming campaigns. | - 3 years follow up using household surveys in June 2005 (baseline) & June 2008 (follow-up) - Adults in the household aged 13–49 years Baseline: 300 households Follow up: 300 households | Food Insecurity, food consumption frequency & diet diversity, vitamin A deficiency anthropometry-stunting, wasting, & underweight | Pathways not reported  Impact  *Household/not specified* ↓Food insecurity (% change: -20.7, P<0.0001),  ↑ Daily consumption of vitamin. A rich animal product (% change: 22.5%, P<0.0001) no impact on daily consumption of vitamin A rich plant products, ↑Food variety score week (% change: 11.7%, P<0.0001)  ↑ No. of daily meals ↑ Mean DDS week (% change: 4.8%, P<0.0001) *Children* No impact on vitamin A. supplementation ↓VAD in children < 5 y (% change: -52.4, P=0.0073)  ↓ Stunting in children < 2 y (% change: - 38.5, P=0.014) ↓ Underweight in children < 2 y (% change: -85, P=0.002) No impact on wasting in children < 2 y *Women*: no impact on VAD in women 13-49 y | High |

AGP, acid glycoprotein, Ag-G-BCC, Ag-G, agriculture-gender; agriculture-gender-behaviour change communication; ARI, acute respiratory tract infection; aOR, adjusted odds ratio; ASF, animal source food; BCC, behaviour change communication; BF, breastfeeding; BMI, body mass index; CBCC, community-based childcare centre; CDD, children’s dietary diversity; CF, complementary feeding; CHV, community health volunteer; CMAM, childhood management of acute malnutrition; CRP, C-reactive protein; DDS, dietary diversity score; EBF, exclusive breastfeeding; EHA, essential hygiene action; EHFP, enhanced homestead food production; ENA, essential nutrition action; FCHV, female community health volunteer; FFS, farmer field school; GLV, green leafy vegetable; HAZ, height-for-age z-score; Hb, haemoglobin; HC, health centre; HDDS, household dietary diversity score; HEW, health extension worker; HFIAS, household food insecurity access scale; HFP, homestead food production; HH, household; IFA, iron folic acid; IP, intensive participation; IT, intensive treatment; ITT, intention-to-treat; IYCF, infant and young child feeding; IYCN, infant and young child nutrition; KAP, knowledge, attitude, practice; LAZ, length-for-age z-score; LMICs, low and lower-middle-income countries; MAD, minimum acceptable diet; MAM, moderate acute malnutrition; MAR, mean adequacy ratio; MCHN , maternal child health and nutrition; MDD, minimum dietary diversity; MMDA, minimum micronutrient density adequacy; MMF, minimum meal frequency; MUAC, mid-upper arm circumference; MNP, micronutrient powder; MT, minimum treatment; MUAC, mid upper arm circumference; NSA, nutrition-sensitive agriculture; OFSP, orange-fleshed sweet potato; OWL, old women leader; PM2A, preventing malnutrition in children under 2 approach; pp, percentage points; RAE, retinol activity equivalents; RBP, retinol blinding protein; RCT, randomized controlled trial; RDI, recommended daily intake; RP, reduced participation; SAM, severe acute malnutrition; SBCC, social behaviour change communication; SE, standard error; TE, treatment effect; VAD, vitamin A deficiency; WASH, water, sanitation and hygiene; WAZ, weight-for-age z-score; WDDS, women’s dietary diversity score; WE, women empowerment; WEG, women empowerment group; WHZ:, weight-for-height z-score; WLZ, weight-for-length s-score, ↑, increase, positive difference; ↓, decrease, positive difference

1. [↑](#footnote-ref-1)
2. [↑](#footnote-ref-2)
